# Discovery of nostatin A, an azole-containing proteusin with prominent cytostatic and pro-apoptotic activity†

**DOI:** 10.1039/d4ob01395f

**Published:** 2024-11-22

**Authors:** Kateřina Delawská, Jan Hájek, Kateřina Voráčová, Marek Kuzma, Jan Mareš, Kateřina Vicková, Alan Kádek, Dominika Tučková, Filip Gallob, Petra Divoká, Martin Moos, Stanislav Opekar, Lukas Koch, Kumar Saurav, David Sedlák, Petr Novák, Petra Urajová, Jason Dean, Radek Gažák, Timo J. H. Niedermeyer, Zdeněk Kameník, Petr Šimek, Andreas Villunger, Pavel Hrouzek

**Affiliations:** a Centre Algatech, Institute of Microbiology, Czech Academy of Sciences, Novohradká 237, Centre Algatech, Institute of Microbiology, Czech Academy of Sciences, 379 01 Třeboň Czech Republic hrouzek@alga.cz; b Department of Medical Biology, Faculty of Science, University of South Bohemia Branišovská 1645/31a 370 05 České Budějovice Czech Republic; c Laboratory of Molecular Structure Characterization, Institute of Microbiology, Czech Academy of Sciences Vídeňská 1083 142 00 Praha 4 Czech Republic; d Institute of Hydrobiology, Biology Centre of the Czech Academy of Sciences Na Sádkách 702/7 370 05 České Budějovice Czech Republic; e Laboratory of Structural Biology and Cell Signaling, Institute of Microbiology, Czech Academy of Sciences Vídeňská 1083 142 00 Praha 4 Czech Republic; f CeMM - Research Center for Molecular Medicine, Austrian Academy of Sciences Lazarettgasse 14 1090 Wien Austria; g Institute of Entomology, Laboratory of Analytical Biochemistry and Metabolomics, Biology Centre of the Czech Academy of Sciences, Branišovská 1160/31 370 05 České Budějovice Czech Republic; h Institute for Developmental Immunology Medical University of Innsbruck, Biocenter, Innsbruck Austria; i Institute of Pharmacy, Freie Universität Berlin Königin-Luise-Str. 2+4 14195 Berlin Germany; j Institute of Pharmacy, Martin Luther University Halle-Wittenberg Hoher Weg 8 06120 Halle (Saale) Germany; k Institute of Molecular Genetics, Czech Academy of Sciences Vídeňská 1083 142 20 Praha; l Laboratory of Antibiotic Resistance and Microbial Metabolomics, Institute of Microbiology, Czech Academy of Sciences Vídeňská 1083 142 00 Praha 4 Czech Republic

## Abstract

Ribosomally synthesized and post-translationally modified peptides (RiPPs) are intriguing compounds with potential pharmacological applications. While many RiPPs are known as antimicrobial agents, a limited number of RiPPs with anti-proliferative effects in cancer cells are available. Here we report the discovery of nostatin A (NosA), a highly modified RiPP belonging among nitrile hydratase-like leader peptide RiPPs (proteusins), isolated from a terrestrial cyanobacterium *Nostoc* sp. Its structure was established based on the core peptide sequence encoded in the biosynthetic gene cluster recovered from the producing strain and subsequent detailed nuclear magnetic resonance and high-resolution mass spectrometry analyses. NosA, composed of a 30 amino-acid peptide core, features a unique combination of moieties previously not reported in RiPPs: the simultaneous presence of oxazole/thiazole heterocycles, dehydrobutyrine/dehydroalanine residues, and a sactionine bond. NosA includes an isobutyl-modified proline residue, highly unusual in natural products. NosA inhibits proliferation of multiple cancer cell lines at low nanomolar concentration while showing no hemolysis. It induces cell cycle arrest in S-phase followed by mitochondrial apoptosis employing a mechanism different from known tubulin binding and DNA damaging compounds. NosA also inhibits *Staphylococcus* strains while it exhibits no effect in other tested bacteria or yeasts. Due to its novel structure and selective bioactivity, NosA represents an excellent candidate for combinatorial chemistry approaches leading to development of novel NosA-based lead compounds.

## Abstract 2

Nostatin A is highly modified ribosomally synthesized and post-translationally modified peptide belonging among proteusins. It inhibits multiple cancer cell lines in nanomolar concentrations and acts *via* cell cycle arrest and subsequent apoptosis.

## Graphical abstract

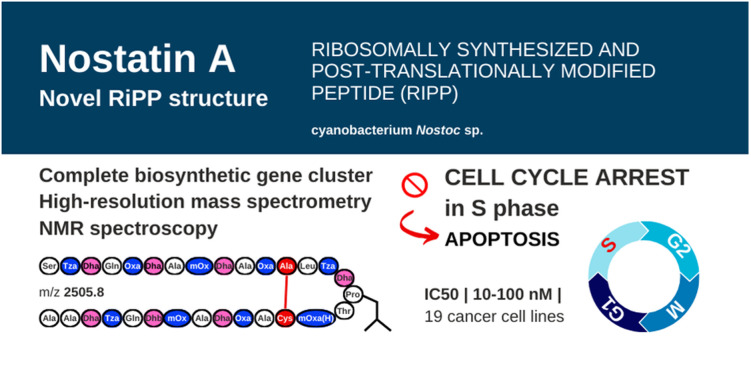

## Introduction

Ribosomally synthesized and post-translationally modified peptides (RiPPs) are striking molecules synthesized by bacteria and fungi, initially *via* proteosynthesis as linear peptides which are subsequently extensively modified by various post-translational mechanisms.^1^ Their specific structural and conformational properties enable enhanced target recognition while also improving resistance to both chemical and metabolic degradation that are common to unmodified linear peptides.^2^ This makes RiPPs particularly attractive as peptide drugs. The basic biosynthetic machinery of RiPPs always involves one or more precursor peptides composed of core and leader peptide sequences. While the core peptide represents the amino-acid sequence that forms the final peptide product, the leader peptide is important for recognition by the post-translational modification enzymes.^1^ Subsequent peptide modification can include macro-cyclization, amino acid heterocyclization, and dehydration. Usually, the post-translational tailoring enzymes are encoded together with the precursor peptide in a biosynthetic gene cluster (BGC), which facilitates bioinformatic prediction of the main structural features of the final product.^3^

One of the most notoriously known RiPP groups are lanthipeptides possessing a thioether linkage created by a cysteine residue and a dehydrated amino-acid residue (typically threonine or serine) which is catalyzed by different types of lanthipeptide synthetases.^4^ Another large groups of RiPPs are linear azole containing peptides (LAPs)^1^ or thiazole–oxazole modified microcins (TOMMs).^5^ These compounds are characterized by the presence of multiple thiazol(in)e and/or (methyl)oxazol(in)e heterocycles formed *via* heterocyclization of Cys, Ser, and Thr residues with an adjacent amino acid. This process is catalyzed by a YcaO cyclodehydratase^6^ and can be followed by the action of a facultative dehydrogenase oxidizing azolines to azole heterocycles, thereby introducing an additional double bond. LAPs and TOMMs are widely found across the bacterial domain including cyanobacteria (cyanobactins)^7^ and other bacterial groups (telomestatins,^8^ microcins,^9^ and goadsporin^10^).

It is noteworthy that particular structural motifs can be combined within one molecule, resulting in a complex final RiPP structure. On top of that, some authors have reported high similarity in the RiPP leader peptide sequences suggesting common evolutionary roots in some RiPPs.^11^ As a result, today's RiPP nomenclature is based on signature enzymes and leader peptide characteristics, however, different RiPP features are often combined in mosaic-like pathways. As an example, bioinformatics surveys have pointed out an abundant class of RiPP BGCs that contain a leader peptide with strong homology to nitrile hydratase. This class of RiPPs entitled proteusins was found to be widely encoded in bacterial genomes and their final products can be very variable.^11–13^ Examples of so far known proteusin members include polytheonamides^14,15^ isolated from a marine sponge *Theonella swinhoei* and landornamides for which the BGC was discovered in the genome of a cyanobacterium *Kamtonema* sp. and subsequently prepared by heterologous expression in *E. coli.*^12^ While polytheonamides exhibit extraordinary strong cytotoxic activity in human leukemia cells *in vitro*,^14^ landornamides exhibit antiviral effects.^12^ This suggest proteusins as a prolific source for development of novel peptide drugs.

Modulation of the cell division machinery still represents one of the main strategies for cancer therapy.^16^ Approximately 60% of the anti-cancer drugs used in medicine are of natural origin or derived from chemical structures found in nature.^17^ Natural products currently used in cancer treatment can be classified into three main groups based on their mechanism of action: DNA damaging agents, drugs inhibiting the key enzymes of DNA metabolism, and drugs altering microtubule dynamics.^18^ However, natural products with alternative modes of action are ready to be approved in the clinic.^18^ Regardless of the afore-mentioned cellular perturbations, tumor cell death is often preceded by a cell cycle arrest phenotype.^19^ The cell cycle can be arrested either in the G1 phase when growth signals are lacking, in S-phase when replication fidelity is compromised, or, in the case of DNA damage, in G1 or prior G2/M transition, while tubulin inhibitors promote the mitotic arrest. When genome integrity cannot be maintained or cells succumb to extended mitotic arrest, this typically culminates in the induction of apoptosis.^19^ In the case of natural cytostatic drugs, apoptosis usually proceeds *via* the “intrinsic pathway” regulated by the Bcl-2 protein family and is executed by family members Bcl-associated X protein (BAX) and Bcl-2 antagonist/killer1 (BAK). The BAX and BAK proteins activate effector caspases 3 and 7 *via* mitochondrial membrane permeabilization which is followed by the degradation phase of apoptosis.^20^

Here, we present the discovery of nostatin A (NosA) from the soil-dwelling cyanobacterium *Nostoc* sp., a novel and highly unusual RiPP with potent selective antimicrobial activity and antiproliferative and proapoptotic effect in cancer cells.

## Results and discussion

In a previous study, we found that a fraction of *Nostoc* sp. CCALA 1144 crude extract increases caspase 3/7 levels in the human cancer cell line PaTu 8902, prompting further investigation.^21^ Analysis of the pro-apoptotic fraction using high resolution mass spectrometry (HRMS) revealed the presence of NosA observed as single, double, and triple charged ions with *m*/*z* 2505.8540 [M + H]^+^, 1253.4360 [M + 2H]^2+^ and 835.9614 [M + 3H]^3+^, respectively. Extraction of 10 g of cyanobacterial biomass with 60% acetonitrile followed by solid phase extraction and two preparative reverse phase HPLC steps yielded 5 mg of the pure compound (Fig. S1†). To elucidate its molecular structure, 1D- and 2D-NMR experiments were performed (Fig. S2–S7†), but these alone could not provide sufficient data for *de novo* structure elucidation and resulted only in fragments. The combination of COSY and ^1^H–^13^C HSQC-TOCSY followed by subsequent ^1^H–^13^C HMBC cross-validation allowed to identify γ-isobutyl-proline (Pro(4-iBu), see supplementary results and Fig. S8–S10†), leucine, threonine, and NHCHCH_2_– and CH_3_CHCH– spin systems (Table S1†). The possible peptide origin of NosA was further suggested by a positive ninhydrin reaction. As multiple attempts to produce crystals suitable for macromolecular crystallography failed, an in-depth mass spectrometric study was initiated to elucidate NosA structure. The simplified scheme of the structure elucidation process is depicted in supplementary file (Fig. S11†).

The MS^2^ fragmentation did not show any fragment ions interpretable as naturally occurring amino acids. Moreover, corresponding neutral losses did not fit any common residues observed in peptides. However, the extended analyses of fragment ions at higher masses revealed multiple occurrences of prominent neutral losses, namely 207 Da, 221 Da, 250 Da, and 257 Da. The building blocks corresponding to these neutral losses are also detectable as charged fragment ions at *m*/*z* 208.07167, *m*/*z* 222.08732, *m*/*z* 251.07748, and *m*/*z* 258.0543, interpretable as C_9_H_10_N_3_O_3_^+^ (*Δ* −1.77 ppm), C_10_H_12_N_3_O_3_^+^ (*Δ* −0.98 ppm), C_10_H_11_N_4_O_4_^+^ (*Δ* 0.07 ppm) and C_9_H_12_N_3_O_4_S_1_^+^ (*Δ* 0.37 ppm), respectively (Table S2 and Fig. S12†). The ring and double bond equivalent (rdb) values of these fragments range between 6 and 8. Such values indicate an exceptionally high number of double bonds and/or multiple cyclization. Tentatively assuming a peptidic character of the compound, all these fragments might be interpreted as modified tripeptides, *e.g.*, the sum formula C_9_H_10_N_3_O_3_^+^ likely corresponds to a double dehydrated and oxidized Ser-Ser-Ala derivative. This infers the presence of dehydrated residues such as dehydroalanine (Dha) or dehydrobutyrine (Dhb) and/or formation of intramolecular heterocycles such as thiazole (Tza), oxazole (Oxa) and methyl-oxazole (mOxa) as frequently found in RiPPs such as TOMMs or LAPs.^1^ Consequently, the above-mentioned fragments can be interpreted as modified tripeptides originating from Cys-Thr-Thr, Ser-Ser-Ala, Thr-Ser-Ala/Ser-Thr-Ala, Ser-Ser-Asn, Ser-Cys-Ser and Ala-Thr-Thr, respectively. Additional targeted MS^3^ fragmentation experiments confirmed that the ion observed at *m*/*z* 936 consists solely of four of the previously mentioned fragments, namely those at *m*/*z* 258, 251, 222, and 208 in this particular order (Fig. S12 and Table S3†). As the fragment with *m*/*z* 222 might equally originate from Ser-Thr-Ala leading to Dha-mOxa-Ala as well as from Thr-Ser-Ala leading to Dhb-Oxa-Ala, two partial hypothetical precursor peptide sequences were proposed: SSAS̲T̲A̲SSNSCS or SSAT̲S̲A̲SSNSCS, differing only in the sequence of the second amino acid triplet.

**Fig. 1:**
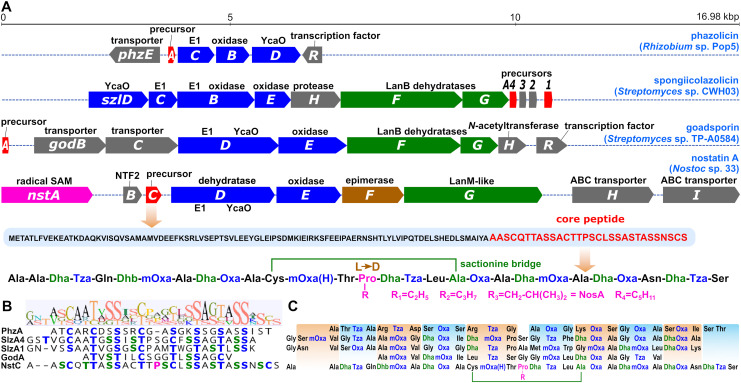
NosA is encoded by a gene cluster similar to thiazole/oxazole modified microcins (TOMMs). (A) Gene map and functional annotation of the biosynthetic gene cluster of NosA compared to known TOMMs. The precursor/core peptide sequence and a schematic representation of the final NosA product including the internal sactionine bridge and sequential methylation of the proline residue are shown below the gene map. Red – precursor peptides, blue – enzymes involved in thiazole/oxazole formation, green – Lan dehydratases, pink – putative SAM-dependent methyltransferase, brown – epimerase, grey – transporters and accessory enzymes. (B) Alignment of the core peptides of selected TOMMs with similar amino acid sequences. Blue – amino acid residues modified to thiazoles/oxazoles, green – dehydrated residues, pink – modified proline. (C) Primary sequences of the selected TOMMs are color-coded as above. Typical structural building blocks, each consisting of three amino acids, are highlighted in boxes.

These partial sequences were used as queries in a genome search in the producer strain, yielding a single hit within a putative BGC of an unknown RiPP. The gene cluster (16.98 kbp, GenBank accession no. OR609362) consisted of nine deduced genes exhibiting homology to known genes in TOMM BGCs (Fig. 1 and Table S4†) and featuring strong homology to nitrile-hydratase leader peptide RiPPs, qualifying the compound among proteusins, a class of RiPPs with only a few known products.^12^ The precursor peptide (NstC) harbored the core peptide sequence AASCQTTASSACTTPSCLSSASTASSNSCS, which includes one of the suggested precursor peptide fragments. Further bioinformatic analyses of proteins encoded in the NosA BGC predicted a thiazoline/oxazoline-forming cyclodehydratase (NstD), possibly catalyzing the cyclization of Cys and Ser/Thr residues, an oxidase involved in the oxidation of thiazolines/oxazolines to thiazoles/oxazoles (NstE), a PoyD-like amino acid epimerase (NstF), and a LanM-like enzyme (NstG) possibly responsible for the dehydration of Ser and Thr residues to Dha and Dhb, respectively.

Interestingly, in addition to the PoyD-like epimerase NstF, the NosA BGC also encodes another radical SAM protein (NstA). As suggested by NMR analysis (Table S1, Fig. S8–S10†), the proline residue present in the structure of NosA is modified to a γ-isobutyl-proline residue. NstA was annotated as a class B cobalamine-dependent *C*-methyltransferase (Table S4 and Fig. S13†). Similar enzymes are involved in iterative *C*-methylation of RiPP compounds such as polytheonamides (PoyB/C)^22^ and non-ribosomal peptide synthetase products such as lapcin (LapC)^23^ forming simple or branched aliphatic side chains. Therefore, we hypothesize that NstA could be responsible for the sequential methylation of the Pro residue in NosA. This prediction was supported by the detection of NosA variants containing a proline residue with side chains shorter by one/two methylene groups in the original *Nostoc* sp. CCALA 1144 extract (see below for further details).

Taking into consideration the primary peptide sequence predicted based on the genome search and the posttranslational modifications including dehydration of Ser/Thr residues and heterocyclization of Ser/Thr/Cys residues, the structure of NosA could be interpreted as ^1^Ala-^2^Ala-^3^Dha-^4^Tza-^5^Gln-^6^Dhb-^7^mOxa-^8^Ala-^9^Dha-^10^Oxa-^11^Ala-^12^Cys-^13^mOxa(H)-^14^Thr-^15^Pro(4-iBu)-^16^Dha-^17^Tza-^18^Leu-^19^Dha-^20^Oxa-^21^Ala-^22^Dha-^23^mOxa-^24^Ala-^25^Dha-^26^Oxa-^27^Asn-^28^Dha-^29^Tza-^30^Ser. The presence of a PoyD-like epimerase (NstF) in the gene cluster suggests that some of the amino acid residues might be present in their d-form. We subjected the NosA peptide to acidic hydrolysis and performed a chiral amino acid analysis. Apart from d-*trans*-4-(Pro(4-iBu), all other amino acids were present in their l-configuration according to our analyses^24,25^ (see supplementary results and Fig. S14 and S15†). This suggests considerable substrate specificity a of the NstF epimerase, which is rather uncommon among RiPP epimerases.^26^

The proposed structure yields the sum formula C_107_H_132_N_32_O_32_S_4_ that fits well to experimentally obtained *m*/*z* 2505.8540 [M + H]^+^ (*Δ* 0.01 ppm), 1253.4356 [M + 2H]^2+^ (*Δ* −0.07 ppm) and 835.9594 [M + 3H]^3+^ (*Δ* −0.12 ppm). The sum formula was additionally corroborated using ^15^N labeled NosA with an observed corresponding increase of the molecular weight by 32 Da resulting in *m*/*z* at 2537.7699 (*Δ* 0.05 ppm) and 1269.3884 (*Δ* 0.12 ppm) for [M + H]^+^ and [M + 2H]^2+^, respectively (Table S5†).

**Fig. 2:**
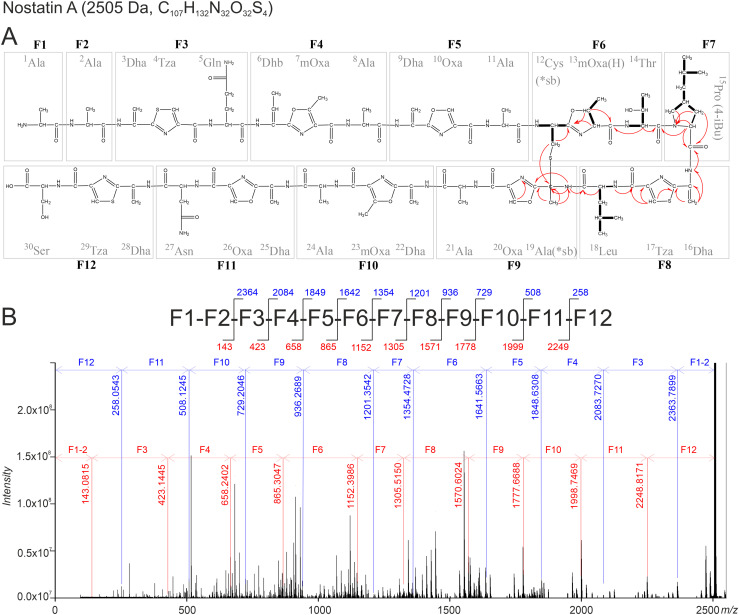
Schematic representation of the structure of NosA showing the integrated results obtained from MS and NMR spectroscopic analyses. (A) Structure of Nos A – individual residues are labeled consecutively from the N-terminus. Main HRMS fragments F1–F12 are indicated with grey boxes. Arrows show the most relevant ^1^H-/^13^C and ^1^H-/^15^N HMBC correlations; COSY correlations are depicted as bolded bonds. (B) Schematic representation of the most informative fragments of NosA (top) and mass spectrum (bottom). Individual y- and b-ions are labeled in red and blue, respectively. The interpretation of the most intense MS fragments which were not used for establishing of the peptide sequence are given in Fig. S16.†

With the results of the gene cluster analysis in hand to guide the assignments, MS^2^ fragmentation identified fragments (F1–F12) confirming the predicted sequence (Fig. 2) with a precision of <0.5 ppm (Table S2†). Due to the presence of Oxa/Tza heterocycles, tripeptide fragments (F3–F6, F8–F12) were highly stable in the performed MS^2^ experiments. In the case of F6 and F12, loss of H_2_S and/or H_2_O was observed, but the peptide bonds remained intact (Table S2†). Except for the fragments F6 and F12 (Fig. 2), all fragments were identified unambiguously, as no other structural solution could be found when taking into consideration the primary peptide sequence. The fragment F12, originating from Ser-Cys-Ser, might theoretically be post-translationally modified into ^28^Dha-^29^Cys-^30^Oxa as well as ^28^Dha-^29^Tza-^30^Ser. The correct interpretation was confirmed by selective MS^3^ fragmentation showing a clear H_2_O loss for the F12 fragment (258.0543 → 240.0438, *Δ* 0.17 ppm), proving the presence of an unmodified free hydroxyl-containing residue (*i.e.*, ^30^Ser), strongly suggesting ^28^Dha-^29^Tza-^30^Ser as the correct substructure. The F6 fragment might be post-translationally modified into ^12^Cys-^13^Thr-^14^mOxa(H) or ^12^Cys-^13^mOxa(H)-^14^Thr. The MS^2^ fragmentation suggested the presence of free thiol and hydroxyl groups supported by a combined loss of H_2_O and H_2_S, leading to *m*/*z* 254.1135 (C_11_H_15_N_3_O_4_ + H^+^, *Δ* −0.13 ppm). The distinct structure of fragment F6 (^12^Cys-^13^mOxa(H)-^14^Thr) was elucidated using NMR (see below). However, a free thiol group in a molecule containing reactive double bonds is highly unlikely, because immediate alkene hydrothiolation is expected to occur. Hence, the presence of a thioether- or thioether-like bond is likely and was confirmed by NMR as well (see below).

The final tripeptide building blocks formed by posttranslational modification share a unified structure containing Dha/Dhb at position 1, heterocycle (Tza/Oxa/mOxa) at position 2 and a natural unmodified amino acid at position 3 (Fig. 2). A very similar modification pattern has been reported in the structures of several bacterial TOMMs such as phazolicin,^27^ spongiicolazolicins A and B,^28^ and goadsporin,^10^ in which, however, the occurrence of dehydrated residues (Dha/Dhb) is less common (Fig. 1). In addition, the published MS^2^ data of spongiicolazolicin B, a compound structurally similar to NosA, reveals an almost identical pattern of fragmentation based on tripeptide building blocks. The F6 fragment of NosA (^12^Cys-^13^mOxa(H)-^14^Thr) is the only tripeptide possessing a residue other than Dha/Dhb at position 1.

The collision-induced dissociation did not provide appropriate information on the overall NosA sequence due to the breakdown of the molecule to fragments below *m*/*z* 1500 only. Thus, infrared-multiple photon dissociation (IRMPD) of selected single- and double-charged ions directly inside the ICR cell was used to gain additional information on the complete fragmentation pathway including the initial losses. The fragmentation of the single charged NosA ion shows interpretable b- and y-ions, including neutral losses of H_2_O, H_2_S, and CO corresponding to subsequent losses of F1 to F12 with sufficient precision (Fig. 2, and Table S5†). The c-ions are also detectable for the majority of the fragments (Table S5†), however, this series is observed only after the initial cleavage of ^1^Ala-^2^Ala.

Due to the high number of aromatic heterocycles and Dha/Dhb residues, NosA might form a tautomeric structure, leading to the reorganization of hydrogens present on the main backbone (Fig. S17†), making the interpetation of NMR complicated. The fragments F6 (^12^Cys-^13^mOxa(H)-^14^Thr), F7 (^15^Pro(4-iBu)) and F8 (^16^Dha-^17^Tza-^18^Leu), however, do not allow higher resonance and corresponding NMR correlations were detected (Fig. S18–S31†). NMR signals interpreted as Leu, Thr, and γ-isobutyl-proline might be assigned as ^18^Leu, ^14^Thr, and ^15^Pro(4-iBu). The previously suggested spin system –NHCHCH_2_– corresponds to ^12^Cys. The CH_3_CHCH– spin system is a part of the methyl-oxazoline ring of ^13^mOxa(H). The substructure containing ^16^Dha and ^17^Tza was supported by ^1^H–^13^C HMBC and ^1^H–^15^N HMBC as depicted in Fig. 2. An HMBC correlation was detected between the methylene of ^12^Cys and a quaternary carbon at 61.5 ppm, which is further coupled with an amide NH at 9.595 ppm and a methyl singlet. Additionally, the NH is coupled to a previously unassigned oxazole/thiazole ring and the carbonyl group of ^18^Leu. This specific correlation indicates the existence of a thioether bridge between ^12^Cys and ^19^Dha.

The Michael addition of a thiol to a dehydroalanine residue would be expected to occur on the exomethylene carbon, resulting in a tertiary alpha- and a secondary beta-carbon atom.^29^ The correlation of the ^12^Cys β-H to a quaternary carbon attached to a methyl group, however, indicates that the thiol group is linked directly to the amino acid alpha-carbon as described for sactipeptides^30^ and not the beta-carbon as observed in lanthipeptides. In the case of sactipeptides, a radical SAM enzyme is required for the initial activation (oxidation) of the alpha-carbon.^30^ As discussed earlier, the radical SAM enzyme encoded by *nstA* is more likely to be linked to sequential *C*-methylation of the ^15^Pro residue (Table S4 and Fig. S13†). NstA exhibited very weak homology and could not be aligned to known radical SAM enzymes involved in sactionine bond formation in compounds such as subtilosin A (AlbA), thuricin (ThnB), thurincin (TrnC/D) or sporulation killing factor (SkfB).^31^ Moreover, in NosA, the ^19^Ser is initially dehydrated to ^19^Dha likely allowing spontaneous formation of sactipeptide bridge due to the existence of a tautomeric structure (Fig. S17,† for detailed explanation, see supplementary results) and finally resulting in ^19^Ala (labeled as ^19^Ala(sb*) because of its inclusion in the sactipeptide bridge). The direct linkage between ^19^Ala(*sb) and ^12^Cys(*sb) is further substantiated by the targeted MS^2^ analyses showing a fragment at *m*/*z* 495.16573 corresponding to F6 + F9 connected *via* a thioether bridge (Table S3†).

The ^5^Pro(4-iBu) residue, proposed to be installed by NstA, is one of the most notable features of NosA, as such a proline functionalization is highly unusual in natural products. In currently described compounds, mostly single methylation on the γ-position is observed.^32–34^ More complex proline side chains are rare, unbranched aliphatic chains are found only in lincomycins, pyrrolobenzodiazepines, and hormaomycin.^35^ However, none of these functionalizations is achieved by (sequential) methylation of proline residue, as they occur *via* cyclization and/or modification of another amino acid.^36,37^ Thus, the installation of an isobutyl moiety directly onto a proline residue represents a so far undescribed posttranslational modification. The hypothesis of sequential methylation of ^15^Pro is supported by the detection of a series of NosA (*m*/*z* 2505.8) analogs, namely compounds with molecular ions at *m*/*z* 2477.8, 2491.8, and 2519.8. MS^2^ experiments suggest that these congeners are identical except for the ^15^Pro residue (Table S6†). The observed 14 Da differences correspond to consecutive methylations leading to C_2_H_5_–, C_3_H_7_–, C_4_H_9_– (NosA) or C_5_H_11_ extensions of the ^15^Pro. Although the discovered NosA BGC was unique in comparison to known RiPP clusters, homologous BGCs were identified in several cyanobacterial and proteobacterial genomes (Fig. S32†). Notably, while variation was found in the majority of amino acid positions of the core peptide sequence, the N- and C-terminal amino acids and the ^15^Pro-^16^Ser-^17^Cys region located roughly in the middle of the peptide were invariant. This may indicate the importance of the ^15^Pro residue for the bioactivity of the compound.

### Cytotoxicity of NosA

**Fig. 3:**
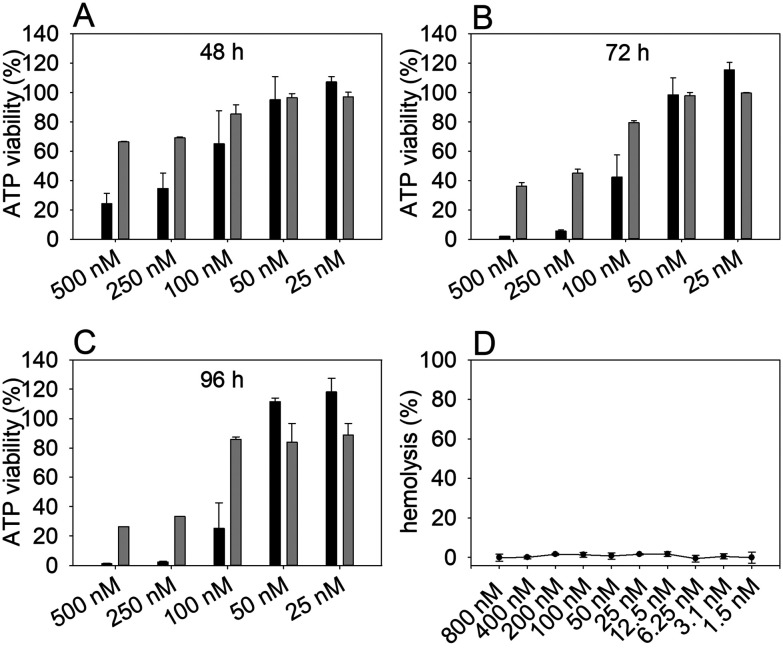
ATP-based viability assay on human cervical cancer cell line HeLa WT (black bars) and primary GK cells (grey bars) and hemolytic assay on sheep red blood cells. The cells were treated with graded doses of NosA and cell viability was evaluated after (A) 48 h, (B) 72 h, and (C) 96 h. Hemolytic activity was evaluated after 2 h exposure (D).

An ATP-based cell viability assay using the human cervical cancer cell line HeLa WT and primary cells isolated from the guinea pig kidney (GK), as well as a hemolytic assay were performed to evaluate the general cytotoxic properties of NosA. The results demonstrated a stronger dose-dependent cytotoxic effect of NosA on HeLa WT cells when compared with milder effect on GK cells over time (Fig. 3A–C). The lowest concentrations (50, 25 nM) tested for NosA did not show significant inhibitory effects in any of the cell lines.

The most prominent difference between HeLa WT and GK viability was recorded using 100 nM NosA even after long-term exposure (96 h) (Fig. 3C). Thus, the concentration of 100 nM was used in subsequent experiments. The calculated IC_50_ values for HeLa WT and GK cell lines at 72 h are shown in Table 1. The hemolytic assay detecting the release of hemoglobin after red blood cell damage^38^ demonstrated that NosA does not induce hemolysis, even at concentrations up to 800 nM (Fig. 3D). Based on these results, NosA seemed suitable for further characterization, as it may not cause general cytotoxicity.

**Table 1:** NosA potency in different cell lines. IC_50_ obtained in ATP-based cell viability assay sorted according to the maximal effect are expressed in nM as mean ± SD from three to six independent measurements. Effect (%) corresponds to the maximum inhibition of NosA obtained (residual viability) at saturating concentrations. Normal/immortalized cells are highlighted in bold

Cell line (origin/classification)	IC_50_ (nM)	Effect (%)
HeLa S3 (cervix/adenocarcinoma)	91 ± 15	2.6 ± 1.1
SJRH-30 (muscle/rhabdomyosarcoma)	28 ± 1.7	5.4 ± 0.1
U-937 (pleural effusion/lymphoma)	17 ± 2.7	5.7 ± 0.6
SW480 (colon/adenocarcinoma)	22 ± 5.4	9.2 ± 0.7
HL-60 (blood/leukemia)	54 ± 2.3	9.3 ± 0.2
HT-29 (colon/adenocarcinoma)	22 ± 2.8	14 ± 4.7
D283 (brain/medulloblastoma)	32 ± 1.7	14 ± 0.4
HCT-116 (colon/carcinoma)	40 ± 19	14 ± 2.9
HEK-293 (kidney/immortalized)	27 ± 2.4	23 ± 0.8
AsPC-1 (pancreas/adenocarcinoma)	41 ± 3.6	24 ± 0.9
RKO (colon/carcinoma)	17 ± 1.9	30 ± 0.4
MDA-MB-231 (breast/adenocarcinoma)	20 ± 4.7	32 ± 20
**Guinea-pig kidney (kidney/normal)**	143 ± 16	33 ± 9.6
**RPE-1 (retina/normal-immortalized)**	19 ± 10	40 ± 6.2
BxPC-3 (pancreas/adenocarcinoma)	56 ± 6.7	41 ± 1.6
K-562 (bone marrow/leukemia)	53 ± 5.6	52 ± 1.2
Capan-2 (pancreas/adenocarcinoma)	37 ± 10	53 ± 3.4
MCF-7 (breast/adenocarcinoma)	10 ± 4.6	56 ± 0.9
**BJ (foreskin/normal)**	69 ± 6.9	62 ± 1.2
Hep G2 (liver/carcinoma)	30 ± 9.9	62 ± 2.6
DU 145 (prostate/carcinoma)	35 ± 11	73 ± 0.2
U-2 OS (bone/osteosarcoma)	22 ± 22	80 ± 9.7
Caov-3 (ovary/adenocarcinoma)	29 ± 10	82 ± 1

### Evaluation of NosA potential against human cell lines and selected microorganisms

NosA was subsequently tested against a broad selection of human cancer and primary/immortalized cell lines as well as a panel of bacteria and fungi. In total, 18 cancer cell lines, two immortalized cell lines, and one primary cell culture of human origin were treated with NosA in a concentration range of 0.2 nM to 7 μM, and the ATP-based viability was determined after 72 h of exposure. The dose–response curves substantially differ among particular cell lines especially in the lowest viability achieved (Table 1, effect in %).

The obtained IC_50_ values vary from 17 to 91 nM, indicating a strong cytotoxic potency of the compound (Table 1 and Fig. S33†). Lymphoma cells U-937, sarcoma cell line SJCHR-30, colon carcinoma cells HT-29, and SW480 were among the most sensitive cell lines. These cell lines exhibited low IC_50_ values and almost full inhibition at high NosA concentrations. On the other hand, most of the tested cancer and control immortalized cell lines or primary cells displayed only partial response with an efficacy of NosA between 25–70% (Table 1, effect in %). The least sensitive cell lines in our screening panel were prostate carcinoma DU-145, ovarian carcinoma CaOV-3, and osteosarcoma U2OS with very low efficacy resulting in viabilities over 70% in a broad concentration range.

The observed resistance of certain cells to NosA, characterized by variable efficacy in cell viability accompanied by minor changes in the potency (IC_50_) can be explained by different effects of NosA on cellular proliferation, delayed induction of cell death, or a shift of the cells toward a senescent phenotype. To shed a light on this observation, we performed a long-term experiment in RPE-1 cells. The cells were treated with NosA, and a washout/retreatment was performed at 72 h, followed by an additional 48 h of recovery/exposure. Low viability obtained at 120 h indicates that these cells responded to NosA with a substantial delay, rather than being resistant to the effect (Fig. S34†).

Concerning antimicrobial activity, NosA exhibited no effect against any of the tested fungal isolates in contrast to the potent effect in human cells, which suggests that NosA does not broadly act on all eukaryotic cells. Notably, NosA exhibited prominent activity against some gram-positive facultative pathogens, such as *Staphylococcus aureus* (MIC of 0.5ug/mL) and *Streptococcus sanguinis* (MIC of 1ug/mL) but not against *Bacillus subtilis* (gram-positive) and gram-negative bacteria (Table S7†). This indicates a specific antimicrobial effect of NosA that agrees with recent reports on RiPPs with the future potential of filling the gap in antibiotic discovery programs.^1,39^

### NosA triggers cell cycle arrest in the S-phase and subsequent apoptosis

**Fig. 4:**
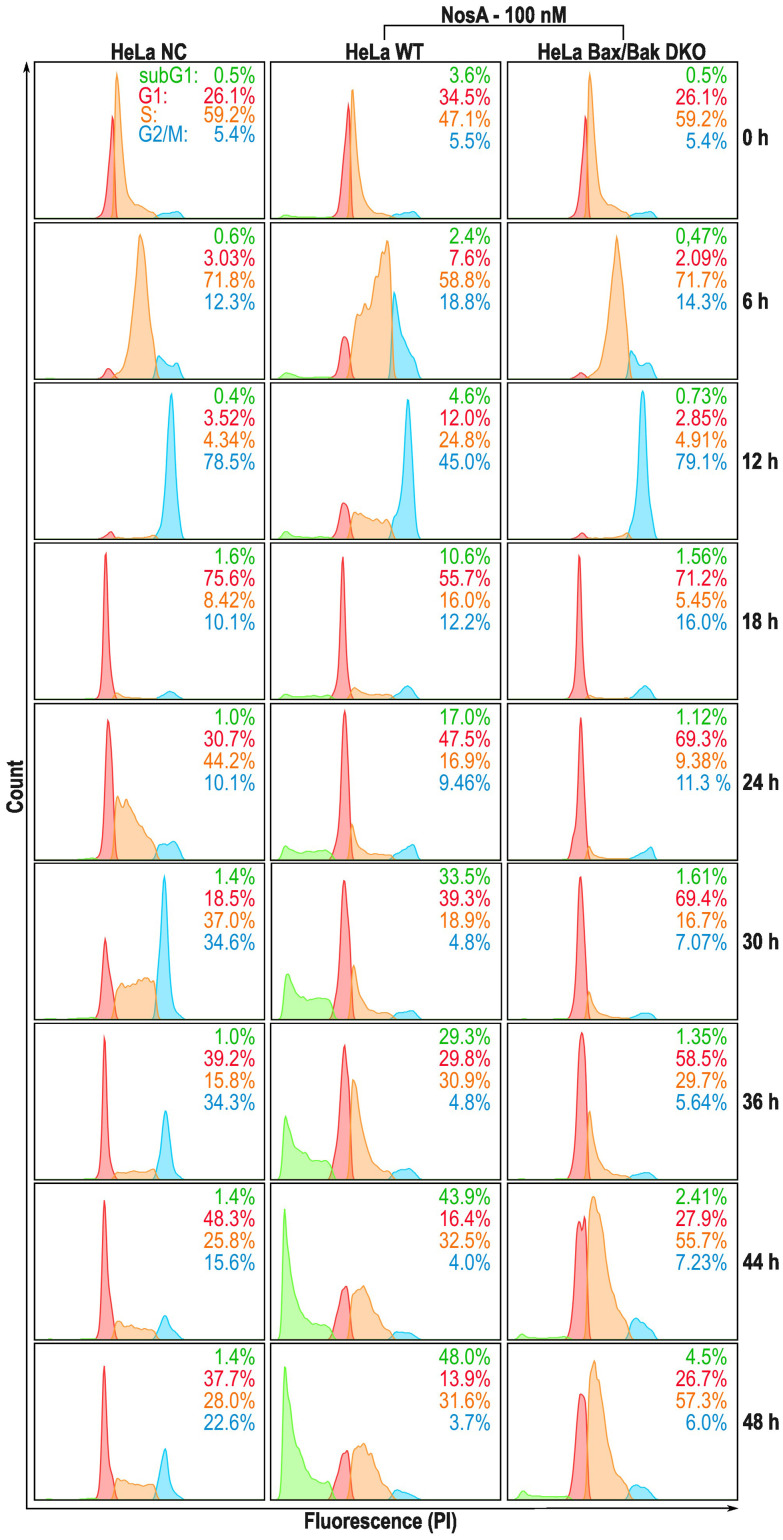
DNA content analysis of NosA-treated HeLa cells by flow cytometry. HeLa WT and Bax/Bak DKO cells were synchronized by a double thymidine block, released into the media with NosA (100 nM) or to control media, stained by propidium iodide (PI), and cell cycle profiles (subG1, G1, S, G2/M) were analyzed.

**Fig. 5:**
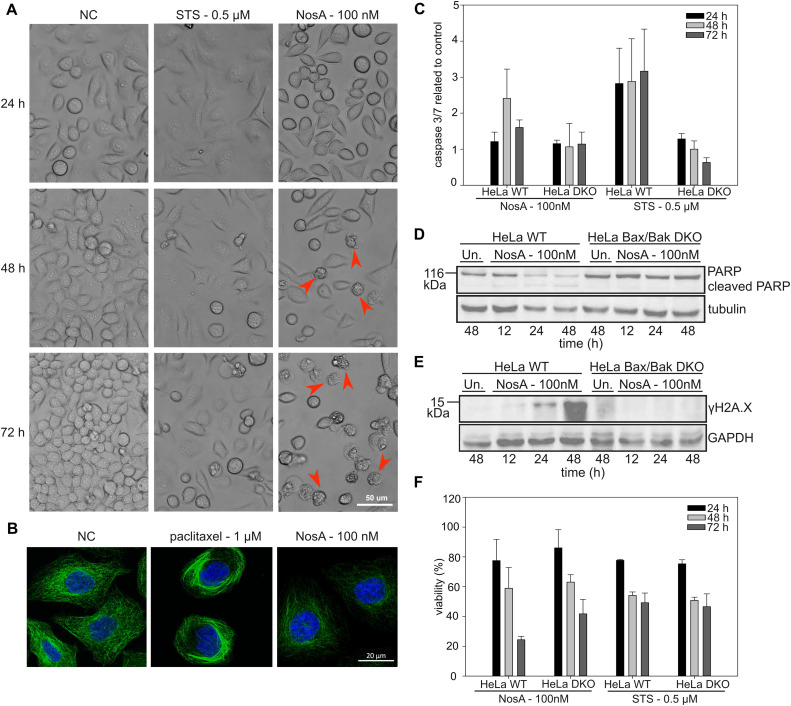
NosA induces mitochondrial apoptosis, while not causing DNA damage or tubulin network alteration. (A) The cytostatic effect shown in HeLa WT cells treated by 100 nM NosA. Red arrows show apoptotic cell blebbing. (STS – stauroporine positive control, NC – negative control). (B) The immunolabeling of tubulin (green) in combination with nucleus staining (blue) in HeLa WT cells. (C) The caspase 3/7 activity of HeLa WT and Bax/Bak DKO cells treated by NosA and STS normalized to control. (D and E) PARP and γH2AX immunoblot of HeLa WT and Bax/Bak DKO cells after treatment with DMOS (Un.) or 100 nM NosA. (F) The viability of HeLa WT and Bax/Bak DKO after NosA treatment. For panels (C and F), the values are expressed as mean ± SD, *n* = 3. The full images of immunoblots are depicted in Fig. S38.†

Our initial microscopic observation pointed towards a cytostatic effect of NosA (Fig. 5A). To bring solid evidence for this mode of action, HeLa wild type (WT) and HeLa Bax/Bak double knock-out (DKO) cells unable to trigger the intrinsic apoptotic pathway, were synchronized with a double thymidine block at the G1/S boundary, released into NosA (100 nM) or DMSO-containing medium (solvent control), and analyzed at particular time points for DNA content (Fig. 4). In the control medium, cells progressed normally through G1-, S-, and G2/M-phase within 12 h and subsequently underwent an additional cell division leading to cell cycle desynchronization and normal cell cycle distribution over time. NosA-treated cells progressed through first cell division normally, while the second division was obstructed. Their cell cycle profile started to differ from the control starting from 24 h, mainly in appearance of a population of cells displaying a lower DNA content (subG1), indicating induction of cell death. Arrest of the cells in the S-phase started at 36 h. In HeLa Bax/Bak DKO cells, the accumulation in S-phase was more profound, as these cells did not transit to the subG1 population. This indicates that the loss of DNA content is probably caused by Bax/Bak-dependent apoptotic cell death. The cytostatic effect of NosA was also observed in asynchronous HeLa cells (WT, Bax/Bak, WT + Q-VD-OPh caspase inhibitor) when analyzed over time in response to 10 and 100 nM NosA (Fig. S35†). All tested cell lines remained arrested in the S-phase of the cell cycle after exposure to 100 nM and, surprisingly, even after 10 nM NosA treatment. Accumulation of cells in the S-phase was most prominent in HeLa Bax/Bak DKO and HeLa WT treated with Q-VD-OPh, which further points to apoptotic cell death. A similar response was observed in HCT-116 colon cancer cells treated with Q-VD-OPh and HCT-116 Bax/Bak DKO variant (Fig. S36†).

All these data clearly show that NosA induces cell cycle arrest before the induction of cell death. The progression of NosA-treated cells through the first mitotic division, as evidenced in the synchronized HeLa cells, also demonstrates that NosA does not belong to the group of tubulin-targeting compounds as many natural cytostatic metabolites such as vinca alkaloids, halicondrins, dolastatins, and taxanes.^40,41^ This is confirmed by the unchanged tubulin network after NosA treatment which is comparable to the negative control (Fig. 5B, and S16†), while tubulin-binding natural products cause typical morphological alteration such as bundle-like tubulin structures (taxol) or vast tubulin network depolymerization (monomethyl auristatin F) (Fig. S37†).

To verify if NosA triggers apoptosis, HeLa WT cells were treated with 100 nM of NosA or staurosporine (STS) as a potent apoptosis inducer, followed by microscopic analysis. Both STS and NosA induced cell shrinkage and blebbing (Fig. 5A). Activation of effector caspases 3/7 was monitored using a luminescence-based assay, in parallel to ATP production. Increased caspase 3/7 activity was observed in HeLa WT but not in HeLa Bax/Bak DKO (Fig. 5C), implying that apoptosis progresses through the mitochondrial pathway. An ATP assay performed on HeLa Bax/Bak DKO (40% viability after 72 h) revealed their higher resistance to cell death compared to the WT (20% viability after 72 h, Fig. 5F).

Moreover, cell lysates were analyzed by western blotting for the appearance of the cleaved variant of PARP1, a *bona fide* effector caspase substrate, along with a marker for DNA damage, γH2A.X. While PARP1 was cleaved and disappeared in HeLa WT cells (Fig. 5D), coinciding with an increase in γH2A.X (Fig. 5E), this was not seen in cells lacking BAX/BAK. Together, this documents the induction of mitochondrial apoptosis. The absence of γH2A.X in Bax/Bak DKO cells also indicates that NosA does not primarily cause DNA damage, as in such case γH2A.X activation would be Bax/Bak-independent.

### Metabolomic study: NosA perturbs purine metabolism

**Fig. 6:**
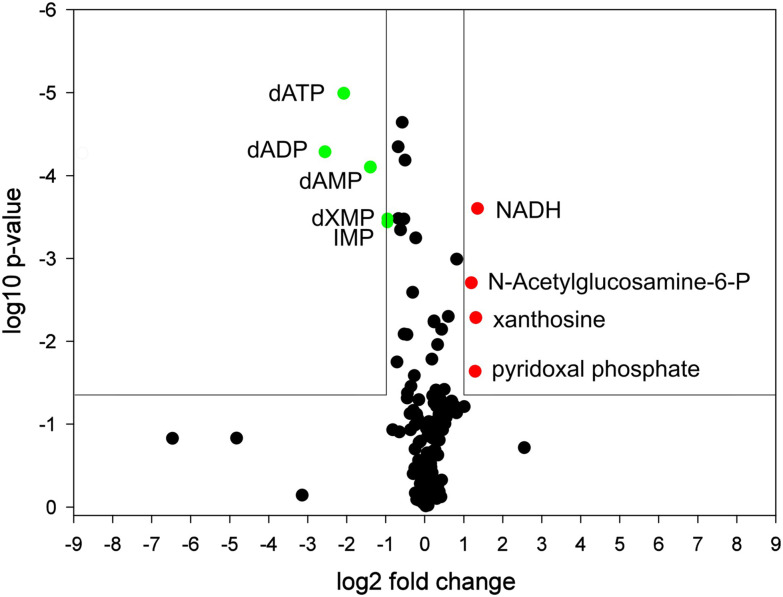
Metabolomic analysis of HeLa WT cells treated with NosA for 24 h visualized with a volcano plot. Individual metabolites are presented by dots referring to their log2 fold change and significance value (log10 *p*-value). Upregulated/downregulated metabolites are highlighted by red/green color, respectively.

Because NosA does not act as a DNA damaging agent or *via* tubulin inhibition, we conducted metabolomic studies^42,43^ to formulate relevant hypotheses on its mechanism of action. Synchronized HeLa WT cells (double thymidine block) were analyzed 6 and 24 h after the release into NosA or DMSO containing medium. The time points were chosen as they were preceding the cell cycle arrest and induction of apoptosis. None of the metabolites was altered after 6 h treatment indicating slow compound effect kinetics. However, severe impairment of purine metabolism was observed at 24 h of treatment (see ESI†), namely in the general precursor of the purine base inosine monophosphate and the deoxynucleotides adenosine-mono-(di-)-(tri-) phosphates (Fig. 6). This result is of great relevance, as deoxynucleotides are direct substrates of DNA synthesis, and their reduced cellular content may be directly related to NosA-induced cell cycle arrest in S-phase. A similar phenotype is caused by ribonucleotide reductase (RNR) inhibitors. RNR is the enzyme responsible for maintaining a balanced supply of deoxyribonucleotides (dNTPs) required for DNA synthesis and repair. The need of dNTPs for proliferation is higher in tumor cells than in normal cells.^44^ RNR is considered a great target for cancer therapy as a small reduction in the dNTPs intracellular levels causes large decreases in DNA synthesis and cell proliferation^45^ and thus several synthetic RNR inhibitors are currently in clinical use. To determine whether NosA is a RNR inhibitor requires further experimental studies.

## Conclusion

NosA is characterized by the simultaneous presence of oxazole/thiazole heterocycles, dehydrobutyrine/dehydroalanine residues, a sactionine bond, and a highly unusual Pro(4-iBu) residue, and belongs into understudied proteusin and sactipeptide RiPP classes. Proteusins are widely spread in cyanobacterial genomes. However, as their BGCs are usually silent, only few compounds have been obtained by heterologous expression. Nostatin thus offers an excellent tool to study the biochemistry and genetics of this compound class.

NosA shows potent anti-proliferative activity in human cancer cells and selective anti-bacterial activity. Its selectivity and high potency predestine NosA for further preclinical studies to test its anti-cancer potential. Finally, the highly unusual structure of NosA offers a wide range of possibilities for combinatorial chemistry aimed at the development of new peptide drugs.

## Author contributions

JH, AV, JM and PH conceptualize the study; PH and KVi discovered the compound; KD, KVo, KVi, PD, FG, KS, DS, DT, JD and PH performed the biological assays; JH, KVi, PU and PH isolated the compound; JH, MK, PN and AK determined the chemical structure of the compound; JM performed the bioinformatic study; MM, SO, PŠ, RG, LK, TJHN and ZK contributed to the stereochemistry of the compound and synthesized the necessary analytical standard; KD, JH, KVo, TJHN, LK, JM, AV and PH wrote the manuscript. All authors contributed to manuscript corrections.

## Ethical statement

All manipulations with animals were performed in accordance with Animal Protection Law of the Czech Republic (No. 246/1992 Coll.) and the institutional ethical committee (University of South Bohemia, Faculty of Science).

## Conflicts of interest

There are no conflicts to declare.

## Supplementary Material

OB-023-D4OB01395F-s001

## Data Availability

The whole-genome assembly (Whole Genome Shotgun project) of the strain *Nostoc* sp. CCALA 1144 (CALU 546) has been deposited at DDBJ/ENA/GenBank under the accession JAWJTF000000000. The version described in this paper is JAWJTF010000000. The sequence of the putative NosA biosynthetic gene cluster can be found at DDBJ/ENA/GenBank under the accession OR609362. The FTICR MS dataset is available in ZENODO under doi: https://doi.org/10.5281/zenodo.10948397. The remaining data that support the findings of this study are available from the corresponding author upon reasonable request.
