# Supplementary material for: Discovery of nostatin A, an azole-containing proteusin with prominent cytostatic and pro-apoptotic activity
Source: Org Biomol Chem. 2024 Nov 22;23(2):449–60. doi: 10.1039/d4ob01395f (PMC11583998; doi:10.1039/d4ob01395f)
Supplement: OB-023-D4OB01395F-s001 [file OB-023-D4OB01395F-s001.pdf]

Supplementary Information (SI) for Organic & Biomolecular Chemistry.
This journal is © The Royal Society of Chemistry 2024

Supporting information to the article

Discovery of nostatin A, an azole-containing proteusin with prominent cytostatic and pro-apoptotic activity

Kateřina Delawská[a,b]†, Jan Hájek[a]†, Kateřina Voráčová[a]†, Marek Kuzma[c], Jan Mareš[a,d], Kateřina Vicková[a], Alan Kádek[e], Dominika Tučková[a,b], Filip Gallob[f], Petra Divoká[a], Martin Moos[g], Stanislav, Opekar[g], Lukas Koch[i,j], Kumar Saurav[a], David Sedlák[k], Petr Novák[e], Petra Urajová[a], Jason Dean[a], Radek Gažák[l], Timo J.H. Niedermeyer[i,j], Zdeněk Kameník[l], Petr Šimek[g], Andreas Villunger[f,h] and Pavel Hrouzek[a]*

[a.] *Centre Algatech, Institute of Microbiology, Czech Academy of Sciences Novohradká 237, 379 01 Třeboň, Czech Republic, hrouzek@alga.cz*

[b.] *Department of Medical Biology, Faculty of Science, University of South Bohemia, Branišovská 1645/31a, 370 05 České Budějovice, Czech Republic*

[c.] *Laboratory of Molecular Structure Characterization, Institute of Microbiology, Czech Academy of Sciences, Vídeňská 1083,142 00 Praha 4, Czech Republic*

[d.] *Institute of Hydrobiology, Biology Centre of the Czech Academy of Sciences, Na Sádkách 702/7, 370 05 České Budějovice, Czech Republic*

[e.] *Laboratory of Structural Biology and Cell Signaling, Institute of Microbiology, Czech Academy of Sciences, Vídeňská 1083,142 00 Praha 4, Czech Republic*

[f.] *CeMM - Research Center for Molecular Medicine, Austrian Academy of Sciences, Lazarettgasse 14,1090 Wien, Austria*

[g.] *Institute of Entomology, Laboratory of Analytical Biochemistry and Metabolomics , Biology Centre of the Czech Academy of Sciences, Branišovská 1160/31, 370 05, České Budějovice, Czech Republic*

[h.] *Division for Developmental Immunology, Medical University of Innsbruck, Biocenter, Innsbruck, Austria*

[i.] *Institute of Pharmacy, Freie Universität Berlin, Königin-Luise-Str. 2+4, 14195 Berlin, Germany*

[j.] *Institute of Pharmacy, Martin Luther University Halle-Wittenberg, Hoher Weg 8, 06120 Halle (Saale), Germany*

[k.] *Institute of Molecular Genetics, Czech Academy of Sciences, Vídeňská 1083, 142 20 Praha*

[l.] *Laboratory of Antibiotic Resistance and Microbial Metabolomics, Institute of Microbiology, Czech Academy of Sciences Vídeňská 1083,142 00 Praha 4, Czech Republic*

†Authors contributed equally to the manuscript

*corresponding author

Materials and methods

Strain cultivation and isolation of NosA

The cyanobacterial strain *Nostoc* sp. CCALA 1144 (CALU 546, Algatech No. 33) is part of the strain collection of the Institute of Microbiology (Centre Algatech) in Třeboň. For inoculum preparation, the cyanobacterium was cultivated in 300 mL glass tubes on Allen Arnon[1] medium under constant conditions: bubbled with 2% CO_2-enriched air at a constant temperature of 28 °C and under a continuous illumination of 50 W/m^2. Large-scale cultivation for compound purification was performed in a 100 L flat-panel bioreactor under identical conditions as mentioned above. For isolation of ^{15}N isotopically substituted nostatin A (NosA), the standard $NaNO_3$ component of the cultivation medium was replaced by $Na^{15}NO_3$ (Sigma Aldrich, PN 364606). The biomass was harvested at the early stationary phase by mesh filtration, concentrated by centrifugation (5000 *g*, 20 min), stored at -80°C, and freeze-dried (CoolSafe, ScanVac) before extraction.
Freeze-dried biomass was subjected to extraction using a pestle and a mortar with sea sand in a ratio of 1g of biomass per 50 mL of 70% acetonitrile (ACN). The suspension was subsequently sonicated for 15 min in a water bath which was followed by a 45 min extraction and additional 15 min sonication step. The supernatant generated by centrifugation (1520 *g*) was collected and the resulting pellet was resuspended in 100% methanol, extracted for 30 minutes, and subjected to the same centrifugation process. The presence of NosA in both fractions was monitored using high-performance liquid chromatography-high-resolution mass spectrometry (HPLC-HRMS) as described below. Obtained extracts were diluted with distilled water to achieve organic content <5% and loaded on separate solid-phase extraction cartridges (Discovery DSC-18, 10g) and eluted with 30%, 50%, 70%, 90%, and 100% methanol in water. The fraction containing NosA (90% MeOH) was evaporated to dryness and resuspended in 60% acetonitrile. The initial purification step was performed on semi-preparative C18 column (YMC ODS-AQ, 250x10 mm, S-5 µm, 20 nm) eluted with water(A)/acetonitrile(B) gradient (0 min - 40%, 10 min - 45%, 46 min - 54%, 58 min - 100%, 65 min - 100%, 66 min - 40%, 74 - 40% of B) at a constant flow rate of 3 mL/min. The compound was detected using wavelengths of 220 nm and 235 nm based on previous confirmation using HPLC-HRMS. Peaks containing NosA were collected manually and their content was further verified using HPLC-HRMS. To prevent column contamination, the column was periodically cleaned with 100% methanol and re-equilibrated using multiple blank injections. The purified fractions were diluted with distilled water to contain approximately 30% of acetonitrile and freeze-dried. The second purification step was performed on a semi-preparative phenyl column (Reprosil 100 phenyl, 250x8 mm, 5 µm) eluted with water (A)/acetonitrile (B) gradient (0 min 30%, 2min 30%, 10 min 45%, 35 min 52%, 36 min 100%. 40 min 100%, 41 min 30%, 46min 30% of B) at a constant flow rate of 3 mL/min. Fractions containing NosA were manually collected, diluted with distilled water, and freeze-dried. The content of the compound varied significantly in the individual biomass batches ranging from 0.5 to 3 mg per 20 g of freeze-dried biomass. The purity used for NMR measurements as well as the bioactivity was >95%.

High-resolution mass spectrometry analyses

HPLC-HRMS analyses were performed using a Dionex UltiMate 3000 HPLC system (Thermo Scientific, Sunnyvale, CA, USA) coupled with a diode array detector (DAD) connected to a high-resolution mass

spectrometer equipped with an electrospray ionization (ESI) source (ESI-HRMS; Impact HD Mass Spectrometer, Bruker, Billerica, MA, USA). The separations were performed on a C18 column (Phenomenex Kinetex C18, 150 × 4.6 mm, 2.6 µm) eluted with water (A)/acetonitrile (B) gradient (0 min 15%, 1 min 15%, 20 min 100%, 25 min 100%, 30 min 15%, 33min 15% of B) at a constant flow rate of 0.6 mL/min. Both solvents were acidified with 0.1% formic acid. The ESI source parameters were as follows: the spray needle voltage was set at 3.8 kV, nitrogen was used both as the nebulizing gas (3 bar) and the drying gas (11 L/min), and the temperature was set to 250 °C. The fragmentation of the molecular ions was induced by nitrogen as a collision gas. NosA was observed mainly in positive mode as multiple charged ion, the fragmentation energy was set to 80-90 eV and 30-40 eV for double-charged and triple-charged, respectively.

Precise mass measurements and MS^2/MS^3 fragmentation experiments were performed using a 15 T SolariX XR Fourier-transform ion cyclotron resonance mass spectrometer (ESI-FTICR MS; Bruker Daltonics, Billerica, MA, USA) equipped with infrared multiple photon dissociation (IRMPD). All experiments were performed using a 2 µl/min direct infusion of NosA dissolved at 10 µM in 60 % acetonitrile acidified with 0.1 % formic acid. The ESI source parameters were as follows: the spray needle voltage was set at 3.9 kV, nitrogen was used both as the nebulizing gas (1.5 bar) and the drying gas (4 L/min), and the drying capillary temperature was 200 °C. Ions were isolated in a quadrupole with a 10 *m/z* selection window, thermalized, and accumulated for 0.5 s inside a 2.2 V potential collision cell with argon as collision gas. Finally, 64 spectra were acquired and summed over the 90 - 3000 *m/z* range with 2 M data points in an ICR Paracell with precise shimming and gated ion injection at 3.0 V axial trapping potential. The instrument was externally calibrated using 0.1% NaTFA clusters and fragmentation spectra were further post-calibrated using 2 peaks (*m/z* 936 and *m/z* 281) in DataAnalysis 5.1 (Bruker Daltonics). For CID, the collision cell potential was increased to 15 - 20 V with 40% Ar flow. For IRMPD experiments inside the ICR cell, a Diamond C-30A CO_2 laser (Coherent, Santa Clara, CA, USA) resonating at 10.6 µm was custom-coupled to the SolariX FTICR MS and axially aligned at the center of the ICR Paracell. The laser was precisely timed in synchronization with the ICR pulse sequence. Mass-selected precursor ions were irradiated immediately before ICR excitation spin, with irradiation times varied between 75 to 250 ms depending on the NosA precursor charge state at 55% laser output power regulated by 5 kHz pulse-width modulation.

NMR spectroscopy

NosA was measured in CD_3OH at 293 K using an NMR spectrometer Bruker Avance III 700 MHz with TCI CryoProbe (700.13 MHz for ^{1}H, 176.04 MHz for ^{13}C, and 70.94 MHz for ^{15}N, Bruker Biospin GmbH, Rheinstetten, Germany). Spectra were referenced by the residual solvent signal as an internal standard (δ_H 3.305 ppm and δ_C 49.04 ppm). We measured ^{1}H NMR, ^{13}C NMR, COSY, ^{1}H-^{13}C HSQC, ^{1}H-^{13}C HMBC, ^{1}H-^{13}C HSQC-TOCSY, *J*-resolved, ^{1}H-^{15}N HSQC, and ^{1}H-^{15}N HMBC spectra using the standard manufacturer's software. The ^{1}H NMR spectrum was zero-filled to 2-fold data points before Fourier transformation. Protons were assigned by COSY and ^{1}H-^{13}C HSQC-TOCSY, and the assignment was transferred to carbons by HSQC. The chemical shifts are given in the δ scale (ppm), and coupling constants are given in Hz. The chemical shifts of carbon and nitrogen atoms were read out from 2D

spectra. The digital resolution allowed us to present the proton and carbon chemical shifts to three and one decimal places, respectively. The proton chemical shift readouts from HSQC are reported to two decimal places.

GC-MS chiral amino acids analysis of NosA hydrolysate

A sample of NosA and was dissolved in methanol and 50 µL (100 µg) was dispensed in 1 mL heavy-wall vial followed by addition of DL-4-fluorophenylalanine (40 nmol) as internal standard. The solution was dried under a gentle stream of nitrogen. Further, 2,3-dimercapto-1-propanesulfonic acid (DMPS) (5 µL, 1.5% aq. solution) and HCl (300 µL) were added and protective argon atmosphere was applied. The vial was sealed and subjected to acidic hydrolysis at two conditions (Condition A: 115°C, 24h, 6M HCl; Condition B 100°C, 2h, 5M HCl). After the hydrolysis, the sample was cooled to room temperature and one half (150 µL) was dried under a stream of nitrogen and subjected to 2,2,3,3,4,4,4-heptafluorobutyl chloroformate (HFBCF) derivatization followed by methyl amidation[2, 3].

Briefly, 25 µL aliquots of an aqueous sodium bicarbonate (100 mmolL^{-1}) and 50 µL of the reactive organic medium (HFBCF: 2,2,3,3,4,4,4-heptafluorobutanol: isooctane = 4: 1: 15, (v/v/v)) were subsequently added to the dried hydrolysate prepared as described above. Next, 25 µL of the catalytic medium (4% pyridine in 1 molL^{-1} aqueous sodium hydroxide) was added and the content was vortexed for 3 s, leaving the dispersed organic phase still cloudy. After another addition of the same volume of the catalytic medium, the arising two-phase system was shaken for 5 s, until the dispersed milky organic phase turned clear. Finally, 100 µL of isooctane and 25 µL of 1 molL^{-1} aqueous HCl were added, the mixture was vortexed for 3 s and the upper organic phase (50 µL aliquot) was transferred into an autosampler vial for chiral GC–MS profiling of primary AAs. Another 50 µL isooctane extract prepared in the previous step was further treated with 50 µL of methylamine solution in ethanol (8 mol L^{-1}). The mixture was kept at 40 °C for 15 min, concentrated under a gentle stream of nitrogen and finally, the residue was dissolved in 50 µL of chloroform before GC–MS analysis.

The obtained extracts were analyzed by GC–MS[2, 3], using a Trace Ultra gas chromatography coupled with a DSQ quadrupole mass spectrometer equipped with a 70 eV EI ion source (all instruments Thermo Scientific, San José, CA, USA). In both cases, a 0.5 µL aliquot was injected using a PTV injector kept at 200 °C, split-flow 25 mL min^{-1}; splitless time 0.7 min. The EI ion source and a GC–MS transfer line were also at 200 °C. Full scan 50–900 Da was used. Both HFBCF derivatives and the amide derivatives were separated on the same CP-Chirasil-L-Val column (25 m × 0.25 mm, 0.12 µm, Agilent Technologies, USA). In the former case, the GC oven temperature program for the HFBOC-HFB analytes started at 89 °C, 1.5 min; 2 °C min^{-1} to 150 °C, 6 min, and then back at 20 °C min^{-1} to 89 °C. For the amide derivatives, the temperature program begun at 110 °C for 1 min; then 5 °C min^{-1} to 180 °C; 1 °C min^{-1} to 200 °C for 8 min ended by a 20 °C min^{-1} cooling to 110 °C to the initial conditions. Helium flow was 1.2 mL min^{-1}.

Genome sequencing and bioinformatics analysis of NosA BGC

Single filaments of *Nostoc* sp. CCALA 1144 were isolated by the glass capillary technique described previously.[4] Briefly, filaments were serially washed in ten drops of TE buffer and the whole genomic DNA was amplified by multiple-displacement amplification (MDA) using the Repli-G Mini Kit (Qiagen). Ten filaments which passed a quality check by 16S rRNA sequencing, were pooled together and sent for commercial *de novo* genome sequencing (EMBL Genomics Core Facility, Heidelberg, Germany) using

an Illumina MiSeq Pair-End library with 250 bp reads, 350 bp average insert length, and 1.2 Gbp data yield. Additionally, total genomic DNA was directly extracted from the strain biomass as follows: (i) the biomass was lyophilized; (ii) approximately 500 mg of dry biomass was pulverized using a mortar and a pestle with liquid nitrogen; (iii) high molecular weight DNA was extracted using the NucleoBond AXG500 column and Buffer Set III (Macherey-Nagel). Subsequently, the extracted DNA was sequenced by the SeqMe company (Dobříš, Czech Republic) using a BAC gDNA library (3-20 kb) in a PacBio RSII single end sequencing run. The sequencing reads from Illumina and PacBio were co-assembled using SPAdes 3.14 de novo assembler[5] with the single-cell option enabled. Protein coding genes were predicted using Prodigal in the assembled scaffolds longer than 999 bp. All predicted proteins were compared to the NCBI-nr database using MMSeqs26 and only contigs that had the majority of hits to cyanobacteria were kept for the rest of the analysis. Completeness of the single cyanobacterial bin was estimated using CheckM.[6]

The NosA BGC was first identified based on a custom BLASTp search against the *Nostoc* sp. CCALA 1144 genome, using the partially predicted amino-acid sequence derived from MS^2 analysis. The genomic surroundings of the single retrieved BLAST hit were analyzed and annotated using antiSMASH 7.0[7] and dedicated BLASTp, CDD[8], and MiBIG 3.0[9] searches, and comparison to previously published bacterial TOMM gene clusters.

The alignment of core peptide sequences and sequences of radical SAM enzymes homologous to NstA was performed in Geneious Prime 2020.0.3 (available from https://www.geneious.com) using the MUSCLE algorithm[10] and adjusted manually. The core peptide sequences of structurally related TOMMs were selected based on a literature search.[11-13] The radical SAM enzymes to be aligned with NstA were selected based on best-matching MiBIG hits and from a literature.[14-16] The most homologous BGCs to the one of NosA were identified based on single protein BLAST searches and the ClusterBlast module search implemented in antiSMASH 7.0.[7]

Cell culture maintenance and preparation of primary culture from guinea pig kidneys

Mycoplasma-free cell cultures (obtained from ATCC culture collection and in-house collection of the Medical University of Innsbruck) were maintained at 37 °C in a humidified atmosphere with 5% CO_2 in RPMI cultivation medium (Gibco Life Technologies) supplemented with 10% FBS (Gibco Life Technologies), 1% antibiotics (Gibco Life Technologies), and L-glutamine 2 mM (Gibco Life Technologies). All manipulations with animals were performed in accordance with Animal Protection Law of the Czech Republic (No. 246/1992 Coll.) and the institutional ethical committee (University of South Bohemia, Faculty of Science). For the preparation of guinea pig kidney primary cells, the kidney was obtained from humanely euthanized young Guinea pig and transferred to sterile PBS. The capsula was removed and the kidney was cut into two halves which was followed by pans and calyces removal. The remaining tissue was weighed, cut into 3 mm fragments and transferred to an Erlenmeyer flask with a stirrer and 50-190 mL of PBS based on the obtained weight. The tissue was rinsed with PBS and transferred to 0.25% solution of trypsin in PBS (20 mL per gram of tissue) and stirred for 5 min. The solution was replaced by a fresh one and the process continued for another 20 min. The supernatant was poured into a container with cooled calf serum. Finally, the cell suspension was centrifuged (~300 *g*, 5 min) and the sediment was diluted in RPMI 1640 medium (Biosera) containing 5% precolostral calf serum (Biosera) and 1% ATB (XC-A4122/100 Penicillin-Streptomycin Solution 100X, Biosera). The cells were seeded into culture bottles at a concentration of $2x10^5$ cells/ml.

Cell viability (ATP assay) and washout experiment

The HeLa WT and primary Guinea pig kidney cells (see above) were seeded into a 96-well flat bottom white plate at a concentration of 1×10^4 cells per well one day before the experiment. The cells were treated with NosA at final concentrations of 500, 250, 100, 50, and 25 nM in three technical replicates. The viability of cells was determined at three different time points (48, 72, 96 h) using the CellTiter-Glo® Luminescent Cell Viability Assay (Promega, G7571) as the endpoint measurement. The assay was performed according to the protocol provided by the manufacturer and luminescence was recorded using a Tecan (Infinite 2000) well-plate reader. Three independent biological experiments were performed and the viability index was calculated as a ratio between the luminescence of the treated and control cells and expressed as a percentage viability value.

The HeLa WT, HeLa Bax/Bak DKO cells were seeded into a 96-well flat bottom white plate at a concentration of 1×10^4 cells per well one day before the experiment. The cells were treated by NosA at a final concentration of 100 nM in three technical replicates. Staurosporine (Sigma, S5921, St. Louis, MO, USA) at 0.5 µM concentration was used as a positive control. The viability of cells was determined at three different time points (24, 48, and 72 h) and the assay was performed as described above.

For the long-term wash-out experiments, the RPE-1 cells were seeded into a white flat-bottom 96-well plate at a concentration of 4×10^3 and 1×10^3 cells per well, respectively, one day before the experiment. The cells were seeded in different concentrations, due to the differential doubling time of the cell lines. The cells were treated with NosA at final concentrations of 500, 100, 30, and 10 nM in hexaplicate. The medium was removed from all wells after 72 h of treatment. One triplicate was re-treated with the same concentrations of NosA (500, 100, 30, 10 nM), and the medium in the other three wells was replaced by fresh medium without NosA (wash out). The cell viability was determined at 168 h using the CellTiter-Glo® Luminescent Cell Viability Assay (Promega) as the endpoint measurement. The assay was performed according to the protocol provided by the manufacturer (Promega) and luminescence was recorded using Omega (BGM Labtech) well-plate reader. Three independent biological experiments were performed and the viability index was calculated as described above.

Hemolytic assay

The hemolytic assay was performed on sheep red blood cells (RBCs) (Thermo Scientific, Czech Republic) following the previously described protocol.[17] Briefly, erythrocytes from sheep blood (500 µl) were washed three times with sterile phosphate buffer saline (pH=7.4), centrifuged (1400 *g*, 5 min) and re-suspended in PBS to obtain a 2%-suspension. 2 µl of NosA solutions were added to 98 µl of the erythrocyte suspension to obtain the final concentration range of 1.5 - 800 nM NosA followed by incubation at room temperature for 2 hours. DMSO (2 µl) was used as negative control and Triton-X (2 µl) as a positive control with final concentrations 0.5% and 0.1%, correspondingly. Samples were centrifuged (9000 *g*, 10 min), and 95 µL of supernatant was transferred to transparent 96-well plates. The absorbance of the supernatant was measured at 570 nm using a Tecan (Infinite 2000) plate reader. The level of hemolysis (%) was calculated using the following formula:

Hemolysis (%) = 100x(Sample-Negative control)/(Positive control-Negative control)

Chemosensitivity profiling using a panel of 21 cell lines

The cell lines BJ, Caov-3, Capan-2, D-283 Med, DU-145, HCT-116, HEK-293, Hep G2, HL-60, HT-29, K-562, MCF-7, MDA-MB-231, RKO, RPE-1, and U-2OS were purchased from public repositories ATCC (the American Type Culture Collection, USA) and DSMZ-German Collection of Microorganisms and Cell Cultures GmbH (Leibniz Institute, Germany). They were maintained in Advanced D-MEM/F-12 medium supplemented with 5% of fetal bovine serum (ThermoFisher Scientific). Cell lines: AsPC-1, BxPc-3, SJCRH30, SW480, and U-937 were grown in Advanced RPMI 1640 Medium supplemented with 5% fetal bovine serum (ThermoFisher Scientific). Cells were harvested the day before the experiment, counted, and filtered with 20-30 µm strainers (pluriSelect). 5 µl or 20 µl of cell suspension was dispensed to each well (1,000 or 4,000 cells/well) of white solid, tissue-culture treated 1536- or 384-well plates (Corning) respectively using a bulk dispenser (Formulatrix, Inc). NosA was pre-diluted in DMSO, reformatted to 384-well plates (Labcyte), and transferred to cells using contact-free acoustic transfer by ECHO 500 series (Labcyte, Inc., USA). NosA was tested over a wide range of concentrations between 0.2 nM and 8 µM. Cell viability was assessed after 72 h of incubation with compounds by determining the level of intracellular ATP using CellTiter-Glo® (Promega) luminescent assay. Plates were equilibrated to room temperature for 20 min, followed by the addition of the luminescent assay reagent. Then, plates were shaken for 5 min, spun down, and the luciferase signal was measured on a multimode plate reader Envision (Perkin Elmer). Data were collected and processed using a proprietary LIMS system ScreenX. The analysis was further performed in GraphPad Prism (Version 10, Boston, MA., USA). Data were normalized to a percentage range where 100% corresponds to the signal from inactive samples and 0% to the signal from samples with no cells. IC_{50} values were obtained using nonlinear regression analysis (Y=Bottom + (Top-Bottom)/(1+10^((LogEC50-X)*HillSlope)).

Synchronization of cells in G1/S by double thymidine block

Cells (HeLa WT, HeLa Bax/Bak DKO) were seeded onto 24-well plates (25×10^3 per a well/500 µL) and left to adhere overnight before the synchronization. Cells were synchronized by double thymidine block as follows: Cells were incubated in medium with thymidine (2 mM) for 20 h, washed twice with PBS, and incubated in fresh medium for 9 h followed by second thymidine block (2 mM) for 17 h. Afterward, cells were washed 2 times with PBS and released into the fresh medium or NosA-containing medium (100 nM). At particular time points (0, 6, 12, 18, 24, 30, 36, 42, 48 h) untreated as well as treated cells were fixed with ethanol for cell cycle analysis as described below. Asynchronous control cells were also involved in the experiment and were used for gating the cell populations in FlowJo software. The experiment was performed in technical triplicates of one biological experiment.

Analysis of cell cycle by flow cytometry

Cells (HeLa WT, HeLa Bax/Bak DKO, HCT-116 WT, HCT-116 DKO) were seeded onto 12-well plates (1×10^5 per 1 mL). Fractions of cells were pre-treated (2 h) with QVD-Oph (20 µM). Cells were treated with NosA (100 nM) for 24, 48, and 72 h, harvested by trypsinization, washed with PBS, subsequently fixed with pre-chilled 70% ethanol, and stored at −20 °C at least overnight. Fixed cells were collected by

centrifugation at 300 *g* for 5 min, washed twice with PBS, and incubated with propidium iodide (40 µg/mL) at 37°C for 30 min in the presence of RNase A (100 µg/mL, Sigma). The stained cells were analyzed directly in a flow cytometer BD FACSCalib™. Primary data were analyzed using FlowJo (FlowJo, LLC) software (doublets and polyploid cells were excluded). For cell cycle analysis of asynchronous cells, three independent experiments were performed.

Immunolabelling of tubulin and nucleus and visualization by confocal microscopy

The HeLa WT cells were grown in a 6-well plate on coated coverslips and treated with 100 nM NosA for 24 hours. The procedure of staining was as follows: Cells were washed twice with pre-warmed PBS, fixed with 3.7% formaldehyde in PBS for 15 min at room temperature (RT), washed two more times with PBS, permeabilized with 0.25% Triton X-100 in PBS for 5 min and again washed two times with PBS. Consequently, the cells were left in 5% bovine serum albumin for one hour at RT, incubated in Alpha-Tubulin Mouse monoclonal Antibody (1 µg/mL) for 3 h at RT, washed two times with PBS and incubated with DAPI (5 µl per well) and Alexa Fluor 488 Rabbit Anti-Mouse IgG Secondary Antibody (1 µl stock solution/200 µl PBS) for 30 min in darkness at RT. Subsequently, the cells were washed two times with PBS. The coverslip with cells was attached to a slide using mounting gel and observed by a confocal microscope the next day. Images were acquired by a laser scanning confocal microscope (Zeiss LSM 880; Carl Zeiss Microscopy GmbH) equipped with a Plan-Apochromatic 63×/1.4 Oil DIC M27 objective. The signal was detected with a GaAsP photomultiplier in 8-bit mode. Cells were excited with 405 nm/488 nm/543 nm lasers for DAPI staining/tubulin staining Alexa Fluor 488; with beam splitters MBS 405/ MBS 488. The contrast in all images acquired was maximized at the white value for DAPI and tubulin at level 150 for better visualization. Three independent biological experiments were performed.

Cell morphology

The HeLa WT cells were seeded to a transparent 96-well cell culture plate, flat bottom at a concentration of 1×10^4 cells per well one day before the experiment. The cells were treated by NosA at a final concentration of 100 nM in three technical replicates. Apoptosis inductor staurosporine (STS) (Sigma, S5921, St. Louis, MO, USA) at 0.5 µM concentration was used as a positive control. The bright-field images were recorded at 24, 48, and 72 h using Axiovision Z.1 Zeiss microscope. Three independent biological experiments were performed and representative figures are shown.

Caspase 3/7 assay

The caspase 3/7 activity in HeLa WT and HeLa Bax/Bak DKO cells was evaluated using Caspase-Glo® 3/7 luminescence assay in 24, 48, and 72 h. Data were completed by quantification of the total number of cells per well using Hoechst 33342 nuclear stain and an automated Fiji macro developed in our laboratory. Subsequently, the obtained caspase 3/7 relative luminescence units (RLU) were adjusted to the cell count and normalized to the control. Staurosporine (STS) 0.5 µM was used as a positive control.

Western blot

HeLa WT cells treated with 100 nM NosA at different time points were harvested, washed twice with PBS, and lysed with RIPA buffer (50 mM Tris HCl pH8, 150 mM NaCl, 1% NP-40, 0.5% sodium deoxycholate, 0.1% SDS) supplemented with a 1x Protease inhibitor (cOmplete™, EDTA-free Protease

Inhibitor Cocktail, 04693132001, Roche). The lysate was then spun down (10 min, 4 °C at 12,000 *g*) and the protein concentration in the supernatant was determined using a BCA kit (Pierce™ BCA Protein Assay Kits, 23225, Thermo Scientific™). 50 µg of proteins per sample were denatured in SDS sample buffer (4x) with DTT (10%). Samples were then subjected to SDS-PAGE and blotted on a nitrocellulose membrane (Amersham™ Protran® Western blotting membranes, nitrocellulose, 10600002, Cytiva™). The membranes were blocked with 5% milk (Powdered milk, T145.3, Carl Roth) in TBS-T for 1 h, and then incubated with the primary antibody overnight at 4 °C. Membranes were washed three times in TBS-T for 10 min and subsequently incubated with the corresponding secondary antibody for 1 h at room temperature. The immunoreactive bands were detected using an ECL KIT (ECL™ Select Western Blotting Detection Reagent, RPN2235, Cytiva™). The antibodies were commercially obtained: PARP (9542, Cell Signaling Technology), GAPDH (sc-32233, Santa Cruz Biotechnology), beta-Tubulin (sc-53140, Santa Cruz Biotechnology), and phospho-H2A.X (07-164, Merck), anti-mouse (7076, Cell Signaling Technology), anti-rat (7077, Cell Signaling Technology).

<u>Antimicrobial assay</u>

The minimum inhibitory concentration (MIC) of purified compound was determined against four phytopathogens; *Monographella cucumerina* (BCC020_2872), *Aspergillus fumigatus* (BCC020_2845), *Fusarium oxysporum* (BCC020_2866), *Alternaria alternata* (BCC020_0609), and five bacterial human pathogens; *Escherichia coli* (CCM2024), *Bacillus subtilis* (CCM1999), *Pseudomonas aeruginosa* (CCM1959), *Staphylococcus aureus* (CCM3824), *Streptococcus sanguinis* (CCM4047), using broth two-fold microdilution method following the standard protocol of broth two-fold microdilution method (CLSI M100-S20).[18] Briefly, for the broth microdilution method, the compound was serially diluted at varying concentrations of 64–0.031 µg/mL in Mueller Hinton broth (with 2% glucose for fungal isolates) in 96-well plates. The inoculum (approximately 5×10^5 CFU/mL as the final concentration) was prepared and added to each well. Culture broth (0.1 mL) was added to each well. Plates were incubated aerobically at 37 °C for 16 h (for bacteria) and 30 °C for 48 h (for fungi). Negative controls were prepared using culture media with 50% DMSO in water (the stock solvent). Erythromycin (32–0.0625 µg/mL) was used as the positive control for *Staphylococcus aureus* and *Streptococcus sanguinis*, Gentamycin (32–0.0625 µg/mL) was used as the positive control for *Bacillus subtilis* and *Pseudomonas aeruginosa*, whereas Fluconazole (32–0.0625 µg/mL) was used as the positive control for all the fungal isolates tested. All the positive controls were also dissolved and prepared in the same way as the tested compound. After incubation, the well with the lowest concentration of the compound showing some inhibition in the growth was taken as the MIC value for the respective organisms. All the experiments were done in triplicates.

<u>Metabolomic analysis</u>

HeLa WT cells (1.5×10^6) were seeded in 5 cm Petri dishes and were synchronized by a double-thymidine block as described above. The experimental design included pentaplicates for control (DMSO treated) as well as compound-treated cells (NosA, 100 nM). The process blanks (background) were prepared as wells containing medium without cells. Three extra petri dishes from every sample were prepared for cell number counting (sample normalization) and flow cytometry analysis (control of cell synchronization). The samples were prepared according to the modified protocol of Martano et. al 2015.[19] The medium was removed from the dish and cells were washed three times using 2 mL of HPLC LC-MS grade water (37˚ C) and subsequently 800 µL of quenching solution (-20 °C)

methanol:acetonitrile: water (2:2:1) was added. The dish was placed on ice and cells were scraped by a cell scraper and harvested into a 5 mL Eppendorf tube placed on ice. The process was repeated with an additional 400 µL of quenching solution. The samples were sonicated using a sonication bath filled with ice water for 1 min and subsequently incubated for 15 min on ice. The samples were placed in a centrifugal evaporator (MiVac Quatro, Genevac^{TM}) with a pre-chilled rotor (-20 ℃) to evaporate organic solvents. The samples were frozen in liquid nitrogen and placed in a deep freezer (-80 °C) for 30 min, dried until complete dryness, and kept at -80 °C before the analysis. Before the analysis, each sample residue was reconstituted with 40 µL 50% acetonitrile, mixed properly (30 s), and placed in the ultrasonic bath (5 min).

The LC-HRMS analysis has been described in detail in Moos et al. 2022.[20] Briefly: A high-resolution Orbitrap Q Exactive Plus mass spectrometer coupled to a Dionex Ultimate 3000 liquid chromatograph and a Dionex open autosampler (all Thermo Fisher Scientific, San Jose, CA, USA) was used for metabolite profiling and quantitative analysis. Metabolites were separated on a 150 mm x 4.6 mm i.d., 5 µm, SeQuant ZIC-pHILIC (Merck KGaA, Darmstadt, Germany) with a mobile phase flow rate of 450 µL/min, an injection volume of 5 µL, and a column temperature of 35 °C. The mobile phase was: A = acetonitrile, B = 20 mmol/L aqueous ammonium carbonate (pH = 9.2; adjusted with NH_4OH); gradient: 0 min, 20% B; 20 min, 80% B; 20.1 min, 95% B; 23.3 min, 95% B; 23.4 min, 20% B; 30.0 min 20% B. The Q-Exactive settings were: Mass range 70-1000 Da; 70 000 resolution (m/z 200; 3 x 10^6 Automatic Gain Control (AGC) target and maximum ion injection time (IT) 100 ms; electrospray operated in positive mod: 3000 kV spray voltage, 350 °C capillary temperature, sheath gas at 60 au, aux gas at 20 au, spare gas at 1 au, probe temperature 350 °C and S-lens level at 60 au. Data were processed using XcaliburTM software, version 2.1 (Thermo Fisher Scientific, San Jose, CA, USA), and an in-house developed metabolite mapper platform.

Supplementary Results

Formation and extended MS evidence for Nos A macrocycle

NosA contains an unusual macrocycle enclosed by a sactipeptide bridge. A sactipeptide bridge is a thioether-type bridge between the thiol group of a cysteine residue and the alpha-carbon of an amino acid. The physicochemical properties of this amino acid seem not to be crucial for the bridge formation, as e.g. Tyr, Met, Asn or Phe are currently described to be involved in sactipeptide bridges.[24] The formation of a sactipeptide bridge is currently proposed to follow two possible mechanisms involving radical SAM and its Fe/S clusters as co-factors.[21] In the first one, S-adenosyl methionine molecule is cleaved by a Fe/S cluster of the rSAM enzyme into free methionine and the 5′-deoxyadenosyl radical subtracting the hydrogen from the α-carbon of an amino acid. In parallel, the second Fe/S cluster is bound to the cysteine residue, resulting in the hydrogen subtraction allowing the subsequent formation of the sactipeptide bridge. In the second scenario, the hydrogen from the cysteine residue is removed by a common base leading to S^-. The α-carbon is activated by a 5′-deoxyadenosyl radical, and subsequently oxidized by two auxiliary Fe/S clusters forming a positively charged N-acyliminium intermediate, which directly binds to the negatively charged cysteine residue.[21]

In the case of NosA, no analog to a sactipeptide radical SAM is included in the biosynthetic gene cluster. As discussed previously, nstC shows homology to a cobalamine-dependent SAM radical enzymes and is likely involved in installation of the Pro(4-iBu) residue. However, due to the high number of aromatic heterocycles and Dha/Dhb residues, NosA might form a tautomeric structure leading to the reorganization of hydrogens present on the main backbone, namely switch between the amide and iminol form of the peptide bonds and relocalization of the double bond present in Dha/Dhb from exomethylene to imine (Fig. S15). Such imines and iminols are structurally similar to the previously mentioned N-acyliminium intermediate, and consequently, we expect the thioether bond might be formed without auxiliary enzymes. The precise mechanism of addition of the thiol to the double bond might be identical to lanthipeptide bridge formation, however, the thioether bridge is not created between the thiol and the exomethylene carbon but between the thiol and the iminol alpha carbon.

The presence of the thioether bridge closing the macrocycle is further supported by targeted MS^2 analyses showing fragments at *m/z* 495.16573 ($C_{20}H_{27}N_6O_7S^+$, Δ -0.17 ppm) and 477.15501 ($C_{20}H_{25}N_6O_6S^+$, Δ -0.15 ppm) interpretable as F6+F9 (^{21}Ala-^{20}Oxa-^{19}Ala(*sb)-^{12}Cys(*sb)-^{13}mOxa(H)-^{14}Thr) with possible water loss, respectively (Tab. S3). Such a fragment would be impossible without an intramolecular bond linking ^{12}Cys and ^{19}Dha. The thioether bond presence was further supported by ions at *m/z* 648.28102 ($C_{29}H_{42}N_7O_8S_1^+$, Δ -0.02ppm) and 630.27065 ($C_{29}H_{40}N_7O_7S_1^+$, Δ -0.32 ppm), corresponding to F6+F7+F9 (^{21}Ala-^{20}Oxa-^{19}Ala(*sb)-^{12}Cys(*sb)-^{13}mOxa(H)-^{14}Thr-^{15}Pro), again with the latter being dehydrated (Tab. S3). To confirm the structural assignment, all fragments used for the structure elucidation were additionally confirmed on the ^{15}N labeled sample (see Tab. S3). The existence of a macrocycle formed by F6-F7-F8-F9 (^{12}Cys(sb*)-^{13}mOxa(H)-^{14}Thr-^{15}Pro(4-iBu)-^{16}Dha-^{17}Tza-^{18}Leu-^{19}Ala(*sb)) explains the stabilization allowing the detection of this part of the molecule in NMR spectroscopy.

Structure elucidation of the γ-isobutyl-proline (Pro(4-iBu)) residue

The identity of Pro(4-iBu) residue was determined using the combination of ^{1}H NMR, COSY, and ^{1}H-^{13}C HMBC spectra.

The terminal methyl of the butyl group appears in the ^{1}H NMR spectrum as two close resonating doublets, which indicates that it is bound to a methine group. Moreover, we observed a correlation of terminal methyl hydrogens with its own carbon at 22.7 ppm, to methine at 27.6 ppm, and methylene at 43.3 ppm in the ^{1}H-^{13}C HMBC spectrum (Fig. S8). The autocorrelation in the ^{1}H-^{13}C HMBC spectrum is a sign of the partial symmetry in the residue, which also well agrees with the iso-butyl group. Further elucidation was also based on COSY. It allowed us to identify the correlation between ^{15}Pro(4-iBu)-H6 and ^{15}Pro(4-iBu)-H7 (Fig. S9). ^{15}Pro(4-iBu)-H6 is further coupled with ^{15}Pro(4-iBu)-H3, which in turn is correlated with two methylene groups, ^{15}Pro(4-iBu)-H2 and ^{15}Pro(4-iBu)-H4 (Fig. S10). These data approve the position of the iso-butyl group in the proline residue because the alternative position of iso-butyl group would give different coupling pattern in the COSY spectrum

Chiral analysis of NosA hydrolysate

NosA was subjected to acid hydrolysis followed by chiral amino acid analysis. Two types of acidic hydrolytic conditions (classical conditions and mild conditions) and two types of hydrolysate derivatizations (2,2,3,3,4,4,4-heptafluorobutyl chloroformate derivatization (HFBCF) and HFBCF with methylamidation[2, 3]) were used. Under mild hydrolytic conditions, L-Leu, L-Thr, L-Ser, L-Cys, L- Ala and to a minor extent D-Ala (2.8 % of total Ala) were detected together with L-Asp and L-Glu (Fig. S13). Cys oxidized to cysteic acid under classical hydrolysis conditions as revealed by LC-MS analysis (data not shown). However, using mild acid hydrolysis and DMPS protection,[22] it was possible to assign the L-Cys configuration. In conclusion, all Nos A proteinogenic amino acids were present in their L-configuration according to our analyses.

The assessment of the stereochemistry of the 4(iBu)Pro residue was done using two approaches. Initially we compared the retention behavior of 4(iBu)Pro with the published literature on retention behavior of 4-substituted prolines.[2, 3] Unfortunately, 4-substituted proline derivatives usually show diastereomeric (trans x cis) and very poor chiral separation on the CP Chirasil-L-Val GC column under HFBCF derivatization conditions.[2, 3] For this reason, we included the analysis of the HFBCF derivatives after amidation, where the D-trans isomer of the 4-substituted proline usually elutes first, followed by the coeluting L-trans and D-cis isomers, and finally the L-cis isomer. It is known that the D-trans isomer of 4-substituted proline racemizes to L-cis and L-trans to D-cis under classical acidic conditions.[23] In NosA, after classical hydrolytic conditions, HFBCF and methylamidation, two peaks of 4(iBu)Pro were formed, indicating that the first larger peak corresponds to the D-trans isomer and the second smaller peak to the L-cis isomer formed by acidic hydrolytic racemization (Fig. S13). Based on this evidence we can conclude that the 4(iBu)Pro residue is present in D-trans configuration.

As no 4(iBu)Pro standards with defined stereochemistry were available, and in order to provide more rigorous evidence, we proceeded with determination of stereochemistry of the ethyl-proline residue naturally occurring in the NosA analog with m/z 2477 (see main manuscript). The standard of L-trans-4-ethylproline was obtained using a published procedure.[24] Peptide 2477 was subjected to acid

hydrolysis under the classical hydrolytic conditions and compared with synthetically prepared 4-ethylproline. The 4-ethylproline analysis of the HFBCF derivatives after amidation showed a similar retention behavior to that of the 4-hydroxyprolines.[2, 3] The D-trans isomer eluted first, followed by the D-cis and L-trans isomers, which are very close to each other, and finally by the L-cis isomer (Fig. S14). The synthetic standard of L-trans-4-ethylproline (non-hydrolyzed) was shown to contain a D-trans impurity (1.1 %). After hydrolysis, peptide 2477 contained mainly D-trans-4-ethylproline and a minor L-cis isomer (2.8%) resulting from acid hydrolysis (Fig. S14 B). All nostatin variants are synthetized by the identical biosynthetic machinery, most likely by iterative methylation of the proline residue. Thus, it is unlikely that the stereochemistry is different among the NosA congeners. The 4(iBu)Pro residue is a result if the iterative methylation of 4-ethyl-proline containing nostatin (i.e. at the already established stereocenter) and thus we can conclude that NosA contains D-trans-4-isobutylproline.

Metabolomic analysis of NosA treated cells

The analyzed metabolomic dataset involved 156 primary metabolites belonging to energy metabolism, nuscleobase base biosynthesis, one-carbon metabolism, amino acid biosynthesis, and vitamins/co-factors. Only very few metabolites were significantly altered (p log2 value ≥ 1, $p < 0.05$) after treatment with NosA. None of the metabolites met the qualification criteria after 6 h treatment with NosA, indicating slow compound effect kinetics. However, after 24 h of exposure to NosA, a subset of significantly altered metabolites was detected (Fig. 6). No alteration was observed in energy metabolism including glycolysis and TCA cycle, showing that energy deprivation is not the main cause of cell death induced by NosA. Severe impairment of purine metabolism was observed, namely in the general precursor of the purine base inosine monophosphate (log2 fold change: -0.98), its degradation product 2'-deoxyxanthosine 5'-monophosphate (log2 fold change: -0.97), and the deoxynucleotides adenosine-mono-(di-)-(tri-) phosphates (log2 fold change: -1.39, -2.56, -2.08, respectively). The metabolomic analysis also revealed increased levels of NADH, pyridoxal phosphate, xanthosine, and N-acetylglucosamine-6-phosphate in NosA-treated cells. For primary data see supplementary dataset.

Supplementary tables

Table S1: NMR characteristics of partial structure (^{12}Cys-^{20}Oxa) of NosA, ^{H} – HSQC readout.

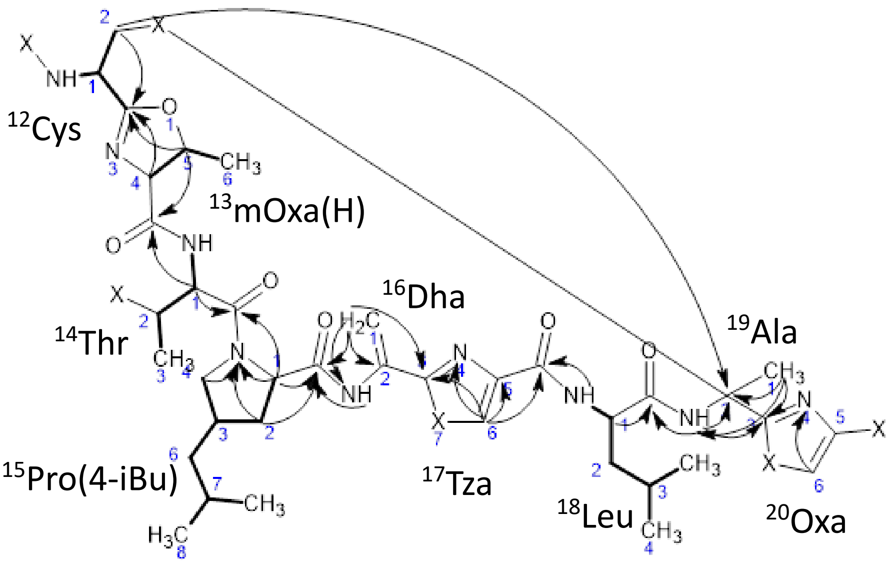

Atom #	δ_C	m	δ_N	δ_H	m	J_{HH}[Hz]
			**^{12}Cys**			
**1**	52.0	d		4.573	m	-
**2**	33.3	t		3.59^{H}	m	-
				2.74^{H}	m	-
**NH**	-		120.1	8.26^{H}	m	-
			**^{13}mOxa(H)**			
**2**	167.2	s		-		
**3(N)**			217.6		m	-
**4**	76.7	d		3.070	d	9.0
**5**	82.8	d		4.350	m	-
**6**	21.4	q		1.267	d	6.4
**4-CO**	173.6	s		-		
			**^{14}Thr**			
**1**	59.8	d		4.601	dd	7.4, 8.5
**2**	66.7	d		4.739	m	-
**3**	19.4	q		1.364	d	6.3
**NH**	-		119.6	8.881	d	7.4
**1-CO**	172.5	s		-		
			**^{15} Pro (4-iBu)**			
**1**	61.6	d		5.048	br d	7.4
**2**	32.9	t		2.686	m	-
				1.55^{H}	m	-
**3**	36.9	d		2.485	m	-
**4**	53.9	t		4.22^{H}	m	-
				3.252	dd	$\sum J$ = 20.2
**5(N)**	-		139.9	-		
**6**	43.3	t		1.39^{H}	m	-
**7**	27.6	d		1.678	m	-
**8**	22.7	q		0.969	d	6.6
				0.967	d	6.6
**1-CO**	171.1	s	-	-		

Table S1 (continuation): NMR characteristics of partial structure ([12]Cys-[20]Oxa) of NosA, [H] – HSQC readout

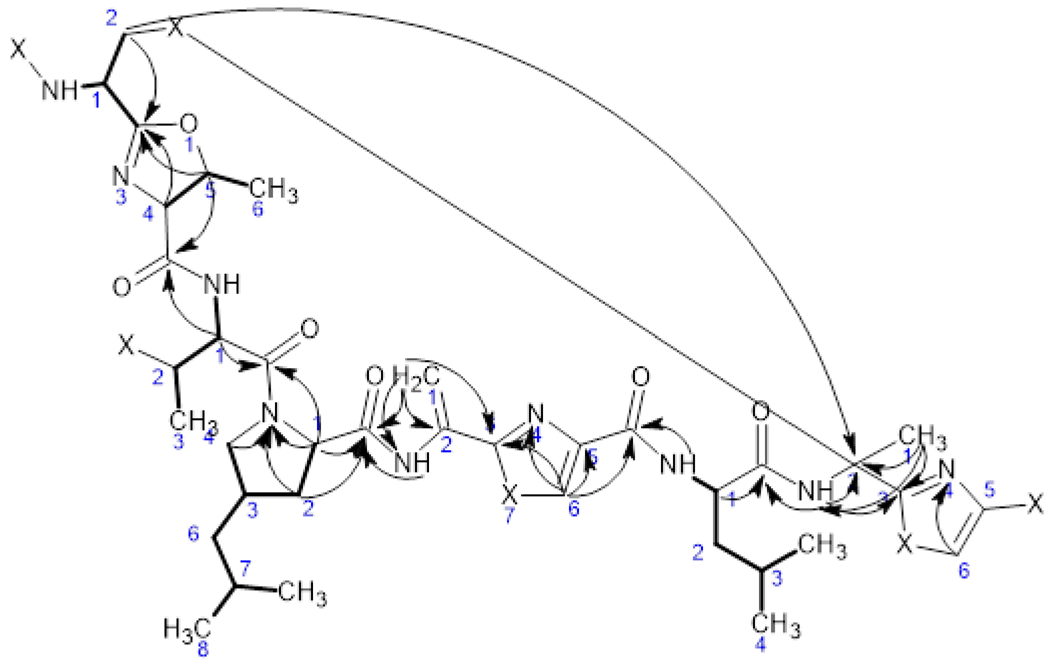

			**[16]Dha - [17]Tza**			
**1**	102.7	t		6.319	m	-
				5.506	m	-
**2**	135.8	s		-		
**3**	166.2	s		-	m	-
**4(N)**	-		302.8	-	m	-
**5**	149.5					
**6**	126.2	d		8.193	s	-
**2-NH**	-		127.8	10.512	s	-
**5-CO**	163.6	s		-		
			**[18]Leu**			
**1**	52.4	d		5.647	m	-
**2**	43.1	t		1.97[H]	m	-
				1.69[H]	m	-
**3**	25.8	d		1.786	m	-
**4**	23.3	q		1.000	d	6.6
	21.5	q		0.913	d	6.6
**NH**			119.4	8.638	d	9.9
**1-CO**	174.6	s				
			**[19]Ala-[20]Oxa**			
**1**	23.2	q	-	2.248	br s	-
**2**	61.5	s	-	-		
**3**	160.4	s		-	-	
**4(N)**	-		249.2	-	-	
**5**	137.5					
**6**	143.6	d		8.267	s	-
**2-NH**	-		132.0	9.595	s	-
**5-CO**	n.d.			-		

[H] – indicates HSQC readout, splitting caused by ^{15}N is not mentioned.

Table S2: NosA and ^{15}N labelled NosA building blocks obtained as MS^2 fragments using infrared-multiple photon dissociation mass spectrometry.

	^{14}N					^{15}N				
**Fragment/loss**	**Sum formula**	**theoretical mass**	**measured mass**	**intensity**	**ppm**	**Sum formula**	**theoretical mass**	**measured mass**	**intensity**	**ppm**
F1	$C_3H_6N_1O_1$+	72.04439	n.a.	n.a.	n.a	$C_3H_6{}^{\mathbf{15}}\mathbf{N}_1O_1$+	73.04143	n.a.	n.a.	n.a
F2	$C_3H_6N_1O_1$+	72.04439	n.a.	n.a.	n.a	$C_3H_6{}^{\mathbf{15}}\mathbf{N}_1O_1$+	73.04143	n.a.	n.a.	n.a
F3	$C_{11}H_{13}N_4O_3S_1$+	281.07029	281.07031	169660512	0.08	$C_{11}H_{13}{}^{\mathbf{15}}\mathbf{N}_4O_3S_1$+	285.05843	285.05839	108734016	-0.13
F4	$C_{11}H_{14}N_3O_3$+	236.10297	236.10298	41303908	0.05	$C_{11}H_{14}{}^{\mathbf{15}}\mathbf{N}_3O_3$+	239.09407	239.09404	15333203	-0.14
F5	$C_9H_{10}N_3O_3$+	208.07167	208.07165	2759369	-0.09	$C_9H_{10}{}^{\mathbf{15}}\mathbf{N}_3O_3$+	211.06277	211.06279	821101	0.08
F6	$C_{11}H_{18}N_3O_4S_1$+	288.10125	n.a.	n.a.	n.a	$C_{11}H_{18}{}^{\mathbf{15}}\mathbf{N}_3O_4S_1$+	291.09236	n.a.	n.a.	n.a
$-H_2O$	$C_{11}H_{16}N_3O_3S_1$+	270.09069	n.a.	n.a.	n.a	$C_{11}H_{16}{}^{\mathbf{15}}\mathbf{N}_3O_3S_1$+	273.08179	n.a.	n.a.	n.a
$-H_2S$	$C_{11}H_{16}N_3O_4$+	254.11353	254.1135	1902971	-0.13	$C_{11}H_{16}{}^{\mathbf{15}}\mathbf{N}_3O_4$+	257.10464	257.10455	2297271	-0.34
F7 -CO	$C_8H_{16}N_1$+	126.12773	126.12776	3808747	0.28	$C_8H_{16}{}^{\mathbf{15}}\mathbf{N}_1$+	127.12476	127.12476	8122119	-0.0
F8	$C_{13}H_{16}N_3O_3S_1$+	294.09069	n.a.	n.a.	n.a	$C_{13}H_{16}{}^{\mathbf{15}}\mathbf{N}_3O_3S_1$+	297.08179	n.a.	n.a.	n.a
-CO	$C_{12}H_{16}N_3O_2S_1$+	266.09577	266.0958	5248618	0.1	$C_{12}H_{16}{}^{\mathbf{15}}\mathbf{N}_3O_2S_1$+	269.08688	269.08687	2064299	-0.03
F9	$C_9H_{10}N_3O_3$+	208.07167	208.07165	2759369	-0.09	$C_9H_{10}{}^{\mathbf{15}}\mathbf{N}_3O_3$+	211.06277	211.06279	821101	0.08
F10	$C_{10}H_{12}N_3O_3$+	222.08732	222.08733	25550688	0.05	$C_{10}H_{12}{}^{\mathbf{15}}\mathbf{N}_3O_3$+	225.07842	225.07839	18778034	-0.15
F11	$C_{10}H_{11}N_4O_4$+	251.07748	251.07748	36677972	-0.01	$C_{10}H_{11}{}^{\mathbf{15}}\mathbf{N}_4O_4$+	255.06562	255.06559	34593808	-0.13
F12	$C_9H_{12}N_3O_4S_1$+	258.0543	258.05433	21823066	0.1	$C_9H_{12}{}^{\mathbf{15}}\mathbf{N}_3O_4S_1$+	261.04541	261.04536	25705980	-0.19
$-H_2O$	$C_9H_{10}N_3O_3S_1$+	240.04374	240.04378	10017250	0.17	$C_9H_{10}{}^{\mathbf{15}}\mathbf{N}_3O_3S_1$+	243.03484	243.03483	8078961	-0.06

Table S3: Fragmentation of NosA higher fragments at *m/z* 913 and 936 and fragments proving in the intramolecular cyclization of NosA molecule.

	^{14}N					^{15}N				
	**Sum formula**	**theoretical *m/z***	**measured *m/z***	**intensity**	**ppm**	**Sum formula**	**theoretical *m/z***	**measured *m/z***	**intensity**	**ppm**
fragmentation of m/z 913 (MS^3 with in-source fragmentation)										
**F5-F6-F7-F8**	$C_{41}H_{57}N_{10}O_{10}S_2$+	913.36951	913.36948	428102528	-0.03	$C_{41}H_{57}{}^{\mathbf{15}}\mathbf{N_{10}}O_{10}S_2$+	923.33986	923.33958	43459428	-0.3
-H_2O	$C_{41}H_{55}N_{10}O_9S_2$+	895.35894	895.35904	107890800	0.11	$C_{41}H_{55}{}^{\mathbf{15}}\mathbf{N_{10}}O_9S_2$+	905.32929	905.32891	11346123	-0.42
-H_2S	$C_{41}H_{55}N_{10}O_{10}S_1$+	879.38179	879.38185	222543536	0.07	$C_{41}H_{55}{}^{\mathbf{15}}\mathbf{N_{10}}O_{10}S_1$+	889.35214	889.35182	30846558	-0.36
**F6-F7-F8**	$C_{32}H_{48}N_7O_7S_2$+	706.30512	706.30509	22511256	-0.04	$C_{32}H_{48}{}^{\mathbf{15}}\mathbf{N_7}O_7S_2$+	713.28436	713.28415	12957270	-0.3
-H_2O	$C_{32}H_{46}N_7O_6S_2$+	688.29455	688.29454	8742430	-0.02	$C_{32}H_{46}{}^{\mathbf{15}}\mathbf{N_7}O_6S_2$+	695.2738	695.27358	7515870	-0.31
-H_2S	$C_{32}H_{46}N_7O_7S_1$+	672.3174	672.31746	119387960	0.1	$C_{32}H_{46}{}^{\mathbf{15}}\mathbf{N_7}O_7S_1$+	679.29664	679.2965	34068440	-0.21
fragmentation of m/z 936 (MS^3 with in-source fragmentation)										
**F9-F10-F11-F12**	$C_{38}H_{42}N_{13}O_{14}S_1$+	936.26894	936.26903	26063350	0.09	$C_{38}H_{42}{}^{\mathbf{15}}\mathbf{N_{13}}O_{14}S_1$+	949.2304	949.23017	3976920	-0.24
**F9-F10-F11**	$C_{29}H_{31}N_{10}O_{10}$+	679.22192	679.22191	8654822	-0.01	$C_{29}H_{31}{}^{\mathbf{15}}\mathbf{N_{10}}O_{10}$+	689.19227	689.19186	1157686	-0.59
**F9-F10**	$C_{19}H_{21}N_6O_6$+	429.15171	429.1517	25333748	-0.02	$C_{19}H_{21}{}^{\mathbf{15}}\mathbf{N_6}O_6$+	435.13392	435.13379	5336273	-0.3
**F10-F11**	$C_{20}H_{22}N_7O_7$+	472.15752	472.15754	23266108	0.03	$C_{20}H_{22}{}^{\mathbf{15}}\mathbf{N_7}O_7$+	479.13677	479.13666	4688799	-0.23
**F10-F11-F12**	$C_{29}H_{33}N_{10}O_{11}S_1$+	729.20455	729.20451	53411468	-0.06	$C_{29}H_{33}{}^{\mathbf{15}}\mathbf{N_{10}}O_{11}S_1$+	739.1749	739.17455	8709984	-0.48
fragmentation of NosA cycle (MS^2)										
**F6-F7-F9**	$C_{29}H_{42}N_7O_8S_1$+	648.28102	648.28101	29033774	-0.02	$C_{29}H_{42}{}^{\mathbf{15}}\mathbf{N_7}O_8S_1$+	655.26026	655.26014	13278066	-0.18
-H_2O	$C_{29}H_{40}N_7O_7S_1$+	630.27065	630.27044	5559035	-0.32	$C_{29}H_{40}{}^{\mathbf{15}}\mathbf{N_7}O_7S_1$+	637.24969	637.24927	3086924	-0.66
**F6-F9**	$C_{20}H_{27}N_6O_7S_1$+	495.16573	495.16564	5829608	-0.17	$C_{20}H_{27}{}^{\mathbf{15}}\mathbf{N_6}O_7S_1$+	501.14786	501.14774	4238934	-0.23
-H_2O	$C_{20}H_{25}N_6O_6S_1$+	477.15501	477.15508	1067751	-0.15	$C_{20}H_{25}{}^{\mathbf{15}}\mathbf{N_6}O_6S_1$+	483.13729	483.13750	1948964	0.43

Table S4: Summary of encoded proteins in the NosA biosynthetic pathway and their closest homologues.

Nostatin A pathway			Top BLASTp hit				Top MIBiG hit with known function			
**Protein**	**Size [aa]**	**Predicted Function**	**Accession**	**Organism**	**Identity [%]**	**Annotation**	**Accession**	**Organism**	**Identity [%]**	**Function**
NstA	694	SAM-radical C-methyltransferase	WP_069070542.1	*Nostoc* sp. KVJ20	99.6	radical SAM protein	AGU42417.1	*Streptomyces argenteolus*	29	CmmK (carbapenem MM4550); putative SAM-radical C-methyltransferase
NstB	138	unknown	WP_069070541.1	*Nostoc* sp. KVJ20	100	ester cyclase	NA	NA	NA	NA
NstC	127	NHLP precursor peptide	WP_069070540.1	*Nostoc* sp. KVJ20	100	nitrile hydratase subunit alpha	NA	NA	NA	NA
NstD	796	cyclodehydratase (oxazoline/thiazoline-forming)	WP_256090978.1	*Nostoc* sp. KVJ20	99.5	TOMM precursor leader peptide-binding protein	ZP_00672901.1	*Trichodesmium erythraeum* IMS101	44	TriA (trichamide); heterocyclization
NstE	492	oxidase (oxazole/thiazole-forming)	WP_069070539.1	*Nostoc* sp. KVJ20	99.8	SagB family peptide dehydrogenase	ZP_00672897.1	*Trichodesmium erythraeum* IMS101	40	TriD (trichamide); oxidase
NstF	475	epimerase	WP_069070538.1	*Nostoc* sp. KVJ20	98.1	radical SAM family RiPP maturation amino acid epimerase	WP_019503883.1	*Pleurocapsa* sp. PCC 7319	40	PlpD (PlpA1); amino acid epimerase
NstG	1075	LanM-like (dehydratase)	WP_069070537.1	*Nostoc* sp. KVJ20	99.1	type 2 lanthipeptide synthetase LanM	BAB04174.1	*Bacillus halodurans* C-125	27	HalM (haloduracin); lanthionine formation
NstH	635	ABC transporter	WP_069070536.1	*Nostoc* sp. KVJ20	99.7	ABC transporter ATP-binding protein/permease	CAG38700.1	*Streptomyces lividus*	32	LivU (lividomycin); ABC transporter
NstI	588	ABC transporter	WP_218108007.1	*Nostoc* sp. KVJ20	99.7	ABC transporter ATP-binding protein/permease	CAD89782.1	*Melittangium lichenicola*	31	ORF7 (melithiazol); ABC transporter

Table S5: NosA and ^{15}N labeled NosA y-/b- ions obtained using IRMPD-MS.

	^{14}N					^{15}N				
**Fragment/loss**	**Sum formula**	**theoretical *m/z***	**measured *m/z***	**intensity**	**ppm**	**Sum formula**	**theoretical *m/z***	**measured *m/z***	**intensity**	**ppm**
$M+H^+$	$C_{107}H_{133}N_{32}O_{32}S_4+$	2505.86411	2505.86349	1348165120	-0.25	$C_{107}H_{133}{}^{15}\mathbf{N_{32}}O_{32}S_4+$	2537.76923	2537.77263	117826328	1.34
$-H_2O$	$C_{107}H_{131}N_{32}O_{31}S_4+$	2487.85354	2487.85315	56239428	-0.16	$C_{107}H_{131}{}^{15}\mathbf{N_{32}}O_{31}S_4+$	2519.75866	2519.7552	5420913	-1.37
$-H_2S$	$C_{107}H_{131}N_{32}O_{32}S_3+$	2471.87639	2471.87776	158864960	0.56	$C_{107}H_{131}{}^{15}\mathbf{N_{32}}O_{32}S_3+$	2503.78151	2503.78567	8593149	1.66
-142 Da (F1-F2)	$C_{101}H_{123}N_{30}O_{30}S_4+$	2363.78988	2363.78985	41163584	-0.01	$C_{101}H_{123}{}^{15}\mathbf{N_{30}}O_{30}S_4+$	2393.70093	2393.70306	5122351	0.89
y1	$C_9H_{12}N_3O_4S_1+$	258.0543	258.05433	21823066	0.1	$C_9H_{12}{}^{15}\mathbf{N_3}O_4S_1+$	261.04541	261.04536	25705980	-0.19
b11	$C_{98}H_{122}N_{29}O_{28}S_3+$	2248.81708	2248.81756	55375592	0.21	$C_{98}H_{122}{}^{15}\mathbf{N_{29}}O_{28}S_3+$	2277.73109	2277.73594	2414166	2.13
$-H_2O$	$C_{98}H_{120}N_{29}O_{27}S_3+$	2230.80651	2230.80864	12146063	0.95	$C_{98}H_{120}{}^{15}\mathbf{N_{29}}O_{27}S_3+$	2259.72053	n.a.	n.a.	n.a
$-H_2S$	$C_{98}H_{120}N_{29}O_{28}S_2+$	2214.82936	2214.83172	23878978	1.07	$C_{98}H_{120}{}^{15}\mathbf{N_{29}}O_{28}S_2+$	2243.74337	2243.74352	1411456	0.07
-142 Da (F1-F2) (b-frag)	$C_{92}H_{112}N_{27}O_{26}S_3+$	2106.74285	2106.74785	26264294	2.37	$C_{92}H_{112}{}^{15}\mathbf{N_{27}}O_{26}S_3+$	2133.6628	2133.66367	1939298	0.41
-142 Da (F1-F2) (c-frag)	$C_{92}H_{115}N_{28}O_{26}S_3+$	2123.7694	2123.77036	25328206	0.45	$C_{92}H_{115}{}^{15}\mathbf{N_{28}}O_{26}S_3+$	2151.68638	n.a.	n.a.	n.a
y2	$C_{19}H_{22}N_7O_8S_1+$	508.12451	508.1245	61636924	-0.02	$C_{19}H_{22}{}^{15}\mathbf{N_7}O_8S_1+$	515.10375	515.10361	20791278	-0.28
b10	$C_{88}H_{112}N_{25}O_{24}S_3+$	1998.74687	1998.74698	176167152	0.05	$C_{88}H_{112}{}^{15}\mathbf{N_{25}}O_{24}S_3+$	2023.67275	2023.67533	8604616	1.28
$-H_2O$	$C_{88}H_{110}N_{25}O_{23}S_3+$	1980.73631	1980.73682	42573864	0.26	$C_{88}H_{110}{}^{15}\mathbf{N_{25}}O_{23}S_3+$	2005.66218	2005.66515	2450915	1.48
$-H_2S$	$C_{88}H_{110}N_{25}O_{24}S_2+$	1964.75915	1964.76022	101721104	0.54	$C_{88}H_{110}{}^{15}\mathbf{N_{25}}O_{24}S_2+$	1989.68503	1989.69003	3789482	2.51
-142Da (F1-F2) (b-frag)	$C_{82}H_{102}N_{23}O_{22}S_3+$	1856.67265	1856.67284	57788008	0.1	$C_{82}H_{102}{}^{15}\mathbf{N_{23}}O_{22}S_3+$	1879.60445	1879.60734	5749361	1.54
-142Da (F1-F2) (c-frag)	$C_{82}H_{105}N_{24}O_{22}S_3+$	1873.6992	1873.69936	28626802	0.09	$C_{82}H_{105}{}^{15}\mathbf{N_{24}}O_{22}S_3+$	1897.62804	1897.6336	1433013	2.93
y3	$C_{29}H_{33}N_{10}O_{11}S_1+$	729.20455	729.20451	53411468	-0.06	$C_{29}H_{33}{}^{15}\mathbf{N_{10}}O_{11}S_1+$	739.1749	739.17455	8709984	-0.48
b9	$C_{78}H_{101}N_{22}O_{21}S_3+$	1777.66683	1777.66782	162357728	0.56	$C_{78}H_{101}{}^{15}\mathbf{N_{22}}O_{21}S_3+$	1799.6016	1799.60414	7937481	1.41
$-H_2O$	$C_{78}H_{99}N_{22}O_{20}S_3+$	1759.65627	1759.65847	37035688	1.25	$C_{78}H_{99}{}^{15}\mathbf{N_{22}}O_{20}S_3+$	1781.59104	1781.59047	2127191	-0.32
$-H_2S$	$C_{78}H_{99}N_{22}O_{21}S_2+$	1743.67911	1743.67955	104701576	0.25	$C_{78}H_{99}{}^{15}\mathbf{N_{22}}O_{21}S_2+$	1765.61388	1765.61604	4423110	1.22
-142 Da (F1-F2) (b-frag)	$C_{72}H_{91}N_{20}O_{19}S_3+$	1635.5926	1635.59173	117299112	-0.53	$C_{72}H_{91}{}^{15}\mathbf{N_{20}}O_{19}S_3+$	1655.5333	1655.53438	9237334	0.65
-142 Da (F1-F2) (c-frag)	$C_{72}H_{94}N_{21}O_{19}S_3+$	1652.61915	1652.61928	77351776	0.08	$C_{72}H_{94}{}^{15}\mathbf{N_{21}}O_{19}S_3+$	1673.55689	1673.55764	3871301	0.45

Table S5 (continuation): NosA and ^{15}N labelled NosA y-/b- ions obtained using IRMPD-MS.

		^{14}N				^{15}N			
**Fragment/loss**	**Sum formula**	**theoretical *m/z***	**measured *m/z***	**intensity**	**ppm**	**Sum formula**	**theoretical *m/z***	**measured *m/z***	**intensity**	**ppm**
y4	$C_{38}H_{42}N_{13}O_{14}S_1$+	936.26894	936.26903	26063350	0.09	$C_{38}H_{42}{}^{15}\mathbf{N}_{13}O_{14}S_1$+	949.2304	949.23017	3976920	-0.24
b8	$C_{69}H_{92}N_{19}O_{18}S_3$+	1570.60244	1570.60248	121339232	0.03	$C_{69}H_{92}{}^{15}\mathbf{N}_{19}O_{18}S_3$+	1589.54611	1589.54788	7112484	1.12
-H_2O	$C_{69}H_{90}N_{19}O_{17}S_3$+	1552.59188	1552.5931	38060532	0.79	$C_{69}H_{90}{}^{15}\mathbf{N}_{19}O_{17}S_3$+	1571.53554	1571.53254	2878272	-1.91
-H_2S	$C_{69}H_{90}N_{19}O_{18}S_2$+	1536.61472	1536.61339	114377408	-0.86	$C_{69}H_{90}{}^{15}\mathbf{N}_{19}O_{18}S_2$+	1555.55838	1555.55991	5999509	0.98
-142 Da (F1-F2) (b-frag)	$C_{63}H_{82}N_{17}O_{16}S_3$+	1428.52821	1428.52559	191005840	-1.84	$C_{63}H_{82}{}^{15}\mathbf{N}_{17}O_{16}S_3$+	1445.47781	1445.47803	14791032	0.15
-142 Da (F1-F2) (c-frag)	$C_{63}H_{85}N_{18}O_{16}S_3$+	1445.55476	1445.55453	223384592	-0.16	$C_{63}H_{85}{}^{15}\mathbf{N}_{18}O_{16}S_3$+	1463.50139	1463.50259	10337331	0.82
y5	$C_{50}H_{57}N_{16}O_{16}S_2$+	1201.35744	1201.3542	3409649	-2.7	$C_{50}H_{57}{}^{15}\mathbf{N}_{16}O_{16}S_2$+	1217.31	n.a.	n.a.	n.a
b7	$C_{57}H_{77}N_{16}O_{16}S_2$+	1305.51394	1305.51497	7944158	0.79	$C_{57}H_{77}{}^{15}\mathbf{N}_{16}O_{16}S_2$+	1322.46600	n.a.	n.a.	n.a
y6	$C_{59}H_{72}N_{17}O_{17}S_2$+	1354.47280	1354.47298	15009932	0.13	$C_{59}H_{72}{}^{15}\mathbf{N}_{17}O_{17}S_2$+	1371.4224	1371.42325	2357744	0.62
b6	$C_{48}H_{62}N_{15}O_{15}S_2$+	1152.39858	1152.40323	3009117	4.04	$C_{48}H_{62}{}^{15}\mathbf{N}_{15}O_{15}S_2$+	1167.3541	n.a.	n.a.	n.a
y7	$C_{70}H_{89}N_{20}O_{21}S_3$+	1641.56678	1641.56631	16393052	-0.29	$C_{70}H_{89}{}^{15}\mathbf{N}_{20}O_{21}S_3$+	1661.50748	1661.50769	1904963	0.12
-H_2O	$C_{70}H_{87}N_{20}O_{19}S_3$+	1623.55621	n.a.	n.a.	n.a	$C_{70}H_{87}{}^{15}\mathbf{N}_{20}O_{19}S_3$+	1643.49691	n.a.	n.a.	n.a
-H_2S	$C_{70}H_{87}N_{20}O_{21}S_2$+	1607.57906	1607.57934	13533878	0.17	$C_{70}H_{87}{}^{15}\mathbf{N}_{20}O_{21}S_2$+	1627.51976	1627.52135	1114746	0.98
b5	$C_{37}H_{45}N_{12}O_{11}S_1$+	865.3046	865.30466	98891296	0.07	$C_{37}H_{45}{}^{15}\mathbf{N}_{12}O_{11}S_1$+	877.26902	877.26852	5302389	-0.57
-142 Da (F1-F2) (b-frag)	$C_{31}H_{35}N_{10}O_9S_1$+	723.23037	723.23046	78297696	0.12	$C_{31}H_{35}{}^{15}\mathbf{N}_{10}O_9S_1$+	733.20072	733.20072	5178934	-0.0
-142 Da (F1-F2) (c-frag)	$C_{31}H_{39}N_{11}O_9S$+	709.29268	n.a.	n.a.	n.a	$C_{31}H_{39}{}^{15}\mathbf{N}_{11}O_9S$+	720.26006	n.a.	n.a.	n.a
y8	$C_{79}H_{98}N_{23}O_{24}S_3$+	1848.63118	1848.63077	46948864	-0.22	$C_{79}H_{98}{}^{15}\mathbf{N}_{23}O_{24}S_3$+	1871.56298	1871.56447	8085660	0.8
-H_2O	$C_{79}H_{96}N_{23}O_{23}S_3$+	1830.62061	1830.62005	29268026	-0.31	$C_{79}H_{96}{}^{15}\mathbf{N}_{23}O_{23}S_3$+	1853.55242	1853.5499	4601808	-1.36
-H_2S	$C_{79}H_{96}N_{23}O_{24}S_2$+	1814.64346	1814.64565	10315803	1.21	$C_{79}H_{96}{}^{15}\mathbf{N}_{23}O_{24}S_2$+	1837.57526	1837.57853	1306599	1.78
b4	$C_{28}H_{36}N_9O_8S_1$+	658.24021	658.24022	79637960	0.02	$C_{28}H_{36}{}^{15}\mathbf{N}_9O_8S_1$+	667.21352	667.21342	14849436	-0.15
-142 Da (F1-F2) (b-frag)	$C_{22}H_{26}N_7O_6S_1$+	516.16598	516.16597	649552960	-0.02	$C_{22}H_{26}{}^{15}\mathbf{N}_7O_6S_1$+	523.14522	523.14515	64684244	-0.14
-142 Da (F1-F2) (c-frag)	$C_{22}H_{30}N_8O_6S$+	502.22828	n.a.	n.a.	n.a	$C_{22}H_{30}{}^{15}\mathbf{N}_8O_6S$+	510.20456	n.a.	n.a.	n.a

Table S5 (continuation): NosA and ^{15}N labelled NosA y-/b- ions obtained using IRMPD-MS

Fragment/loss	^{14}N Sum formula	^{14}N theoretical *m/z*	^{14}N measured *m/z*	^{14}N intensity	^{14}N ppm	^{15}N Sum formula	^{15}N theoretical *m/z*	^{15}N measured *m/z*	^{15}N intensity	^{15}N ppm
y9	$C_{90}H_{111}N_{26}O_{27}S_3+$	2083.72687	2083.72699	6643874	0.06	$C_{90}H_{111}{}^{15}\mathbf{N_{26}}O_{27}S_3+$	2109.64978	n.a.	n.a.	n.a
-H_2O	$C_{90}H_{109}N_{26}O_{26}S_3+$	2065.7163	n.a.	n.a.	n.a	$C_{90}H_{109}{}^{15}\mathbf{N_{26}}O_{26}S_3+$	2091.63921	n.a.	n.a.	n.a
-H_2S	$C_{90}H_{109}N_{26}O_{27}S_2+$	2049.73915	n.a.	n.a.	n.a	$C_{90}H_{109}{}^{15}\mathbf{N_{26}}O_{27}S_2+$	2075.66206	n.a.	n.a.	n.a
b3	$C_{17}H_{23}N_6O_5S_1+$	423.14452	423.14447	8308957	-0.11	$C_{17}H_{23}{}^{15}\mathbf{N_6}O_5S_1+$	429.12673	429.12663	10002351	-0.22
-142 Da (F1-F2)	$C_{11}H_{13}N_4O_3S_1+$	281.07029	281.07031	169660512	0.08	$C_{11}H_{13}{}^{15}\mathbf{N_4}O_3S_1+$	285.05843	285.05839	108734016	-0.13
y10	$C_{101}H_{123}N_{30}O_{30}S_4+$	2363.78988	2363.78985	41163584	-0.01	$C_{101}H_{123}{}^{15}\mathbf{N_{30}}O_{30}S_4+$	2393.70093	2393.70306	5122351	0.89
-H_2O	$C_{101}H_{121}N_{30}O_{29}S_4+$	2345.77931	2345.78096	16654154	0.7	$C_{101}H_{121}{}^{15}\mathbf{N_{30}}O_{29}S_4+$	2375.69036	2375.69089	2231003	0.22
-H_2S	$C_{101}H_{121}N_{30}O_{30}S_3+$	2329.80216	2329.80691	21011294	2.04	$C_{101}H_{121}{}^{15}\mathbf{N_{30}}O_{30}S_3+$	2359.71321	2359.71148	1602807	-0.73
b2	$C_6H_{11}N_2O_2+$	143.0815	143.0815	1234491	-0.03	$C_6H_{11}{}^{15}\mathbf{N_2}O_2+$	145.07557	145.07559	1027487	0.11

Table S6: MS^2 fragmentation pattern of naturally occurring NosA variants differing in the modification of the proline moiety.

Fragment/ loss		2519.8 Sum formula	theoretical *m/z*	measured *m/z*	intensity	ppm	2504.8 Sum formula	theoretical *m/z*	measured *m/z*	intensity	ppm
F1		$C_3H_6N_1O_1+$	72.04439	n.a.	n.a.	n.a.	$C_3H_6N_1O_1+$	72.04439	n.a.	n.a.	n.a
F2		$C_3H_6N_1O_1+$	72.04439	n.a.	n.a.	n.a.	$C_3H_6N_1O_1+$	72.04439	n.a.	n.a.	n.a
F3		$C_{11}H_{13}N_4O_3S_1+$	281.07029	281.0703	7569	0.04	$C_{11}H_{13}N_4O_3S_1+$	281.07029	281.07031	169660512	0.08
F4		$C_{11}H_{14}N_3O_3+$	236.10297	236.1023	1289	-2.83	$C_{11}H_{14}N_3O_3+$	236.10297	236.10298	41303908	0.05
F5		$C_9H_{10}N_3O_3+$	208.07167	208.0713	5311	-1.77	$C_9H_{10}N_3O_3+$	208.07167	208.07165	2759369	-0.09
F6		$C_{11}H_{18}N_3O_4S_1+$	288.10125	288.1005	290	-2.62	$C_{11}H_{18}N_3O_4S_1+$	288.10125	n.a.	n.a.	n.a
	$-H_2O$	$C_{11}H_{16}N_3O_3S_1+$	270.09069	270.0901	185	-2.18	$C_{11}H_{16}N_3O_3S_1+$	270.09069	n.a.	n.a.	n.a
	$-H_2S$	$C_{11}H_{16}N_3O_4+$	254.11353	254.1127	288	-3.28	$C_{11}H_{16}N_3O_4+$	254.11353	254.1135	1902971	-0.13
**F7**	-CO	**$C_9H_{18}N_1+$**	**140.14337**	**140.1433**	**21943**	**-0.54**	**$C_8H_{16}N_1+$**	**126.12773**	**126.12776**	**3808747**	**0.28**
F8		$C_{13}H_{16}N_3O_3S_1+$	294.09069	n.a.	n.a.	n.a.	$C_{13}H_{16}N_3O_3S_1+$	294.09069	n.a.	n.a.	n.a
	-CO	$C_{12}H_{16}N_3O_2S_1+$	266.09577	266.0976	2265	6.86	$C_{12}H_{16}N_3O_2S_1+$	266.09577	266.0958	5248618	0.1
F9		$C_9H_{10}N_3O_3+$	208.07167	208.0871	5311	-1.77	$C_9H_{10}N_3O_3+$	208.07167	208.07165	2759369	-0.09
F10		$C_{10}H_{12}N_3O_3+$	222.08732	222.0871	11122	-0.98	$C_{10}H_{12}N_3O_3+$	222.08732	222.08733	25550688	0.05
F11		$C_{10}H_{11}N_4O_4+$	251.07748	251.0775	8074	0.07	$C_{10}H_{11}N_4O_4+$	251.07748	251.07748	36677972	-0.01
F12		$C_9H_{12}N_3O_4S_1+$	258.0543	258.0544	11886	0.37	$C_9H_{12}N_3O_4S_1+$	258.0543	258.05433	21823066	0.1
	$-H_2O$	$C_9H_{10}N_3O_3S_1+$	240.04374	240.0434	1189	-1.41	$C_9H_{10}N_3O_3S_1+$	240.04374	240.04378	10017250	0.17

Table S6 (continuation): MS^2 fragmentation pattern of naturally occurring NosA variants differing in the modification of the proline moiety.

Fragment/ loss		2491.8 Sum formula	theoretical mass	measured mass	intensity	ppm	2477.8 Sum formula	theoretical mass	measured mass	intensity	ppm
F1		$C_3H_6N_1O_1$+	72.04439	n.a.	n.a.	n.a	$C_3H_6N_1O_1$+	72.04439	n.a.	n.a.	n.a
F2		$C_3H_6N_1O_1$+	72.04439	n.a.	n.a.	n.a	$C_3H_6N_1O_1$+	72.04439	n.a.	n.a.	n.a
F3		$C_{11}H_{13}N_4O_3S_1$+	281.07029	281.0702	23457	-0.31	$C_{11}H_{13}N_4O_3S_1$+	281.07029	281.0699	173581	-1.38
F4		$C_{11}H_{14}N_3O_3$+	236.10297	236.1027	4557	-1.14	$C_{11}H_{14}N_3O_3$+	236.10297	236.14024	65484	-2.41
F5		$C_9H_{10}N_3O_3$+	208.07167	208.0718	16232	0.63	$C_9H_{10}N_3O_3$+	208.07167	208.0717	50648	0.15
F6		$C_{11}H_{18}N_3O_4S_1$+	288.10125	288.1007	555	-1.92	$C_{11}H_{18}N_3O_4S_1$+	288.10125	288.1010	6522	-0.88
	-H_2O	$C_{11}H_{16}N_3O_3S_1$+	270.09069	270.0900	634	-2.55	$C_{11}H_{16}N_3O_3S_1$+	270.09069	270.0902	5742	-1.81
	-H_2S	$C_{11}H_{16}N_3O_4$+	254.11353	254.1128	787	-2.88	$C_{11}H_{16}N_3O_4$+	254.11353	254.1131	11314	-1.7
**F7**	-CO	**$C_7H_{14}N_1$+**	**112.11208**	**112.1119**	**73626**	**-1.56**	**$C_6H_{12}N_1$+**	**98.09643**	**98.0963**	**257480**	**-1.21**
F8		$C_{13}H_{16}N_3O_3S_1$+	294.09069	n.a.	n.a.	n.a.	$C_{13}H_{16}N_3O_3S_1$+	294.09069	n.a.	n.a.	n.a.
	-CO	$C_{12}H_{16}N_3O_2S_1$+	266.09577	266.0960	6147	0.85	$C_{12}H_{16}N_3O_2S_1$+	266.09577	266.0953	14876	-1.78
F9		$C_9H_{10}N_3O_3$+	208.07167	208.0718	16232	0.63	$C_9H_{10}N_3O_3$+	208.07167	208.0717	50648	0.15
F10		$C_{10}H_{12}N_3O_3$+	222.08732	222.0875	39217	0.82	$C_{10}H_{12}N_3O_3$+	222.08732	222.0780	230742	-1.43
F11		$C_{10}H_{11}N_4O_4$+	251.07748	251.0772	28827	-1.12	$C_{10}H_{11}N_4O_4$+	251.07748	251.0773	105385	-0.72
F12		$C_9H_{12}N_3O_4S_1$+	258.0543	258.0543	36777	-0.02	$C_9H_{12}N_3O_4S_1$+	258.0543	258.0540	145756	-1.18
	-H_2O	$C_9H_{10}N_3O_3S_1$+	240.04374	240.043	2626	-3.08	$C_9H_{10}N_3O_3S_1$+	240.04374	240.0434	21457	-1.41

Table S7: Determination of minimum inhibitory concentration for NosA against gram-positive, gram-negative bacteria, and pathogenic fungi. The MIC value for the standard antibiotic used as a positive control (µg/mL) is given in parentheses. NA—no activity.

**Tested microorganisms**	**MIC (µg/mL)**
**Bacteria**	
Escherichia coli (1)	NA
Bacillus subtilis (4)	NA
Pseudomonas aeruginosa (16)	NA
Staphylococcus aureus (2)	0.5
Streptococcus sanguinis (8)	1
**Fungi**	
Candida friedrickii (8)	NA
Aspergillus fumigatus (32)	NA
Fusarium oxysporum (8)	NA
Monographella cucumerina (2)	NA
Alternaria alternata (2)	NA

Supplementary figures

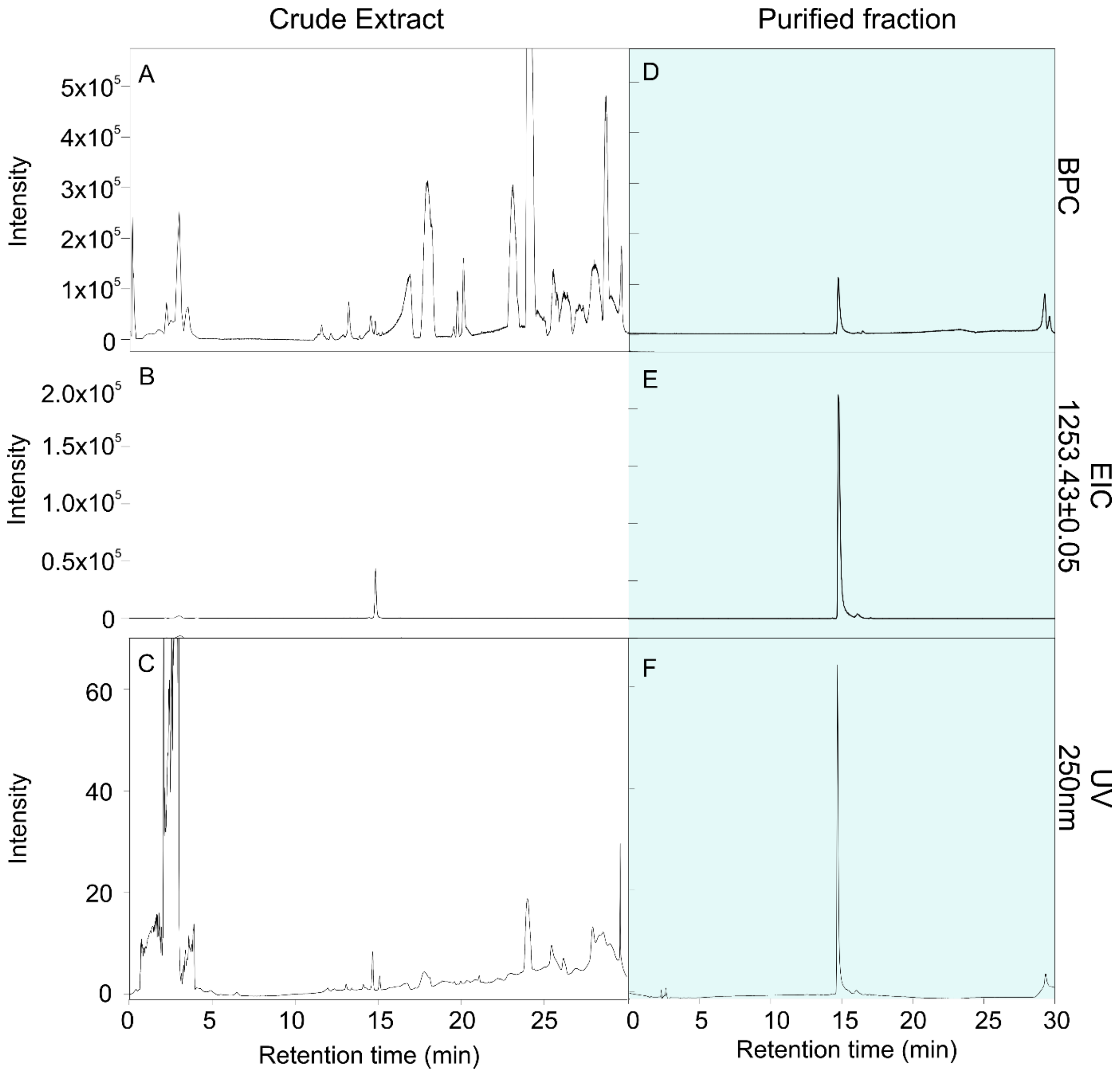

Figure S1: Purification of NosA: Base peak chromatogram, extracted ion chromatogram of NosA, and UV trace at 250 nm for crude extract (A, B, C) and purified NosA (D, E, F).

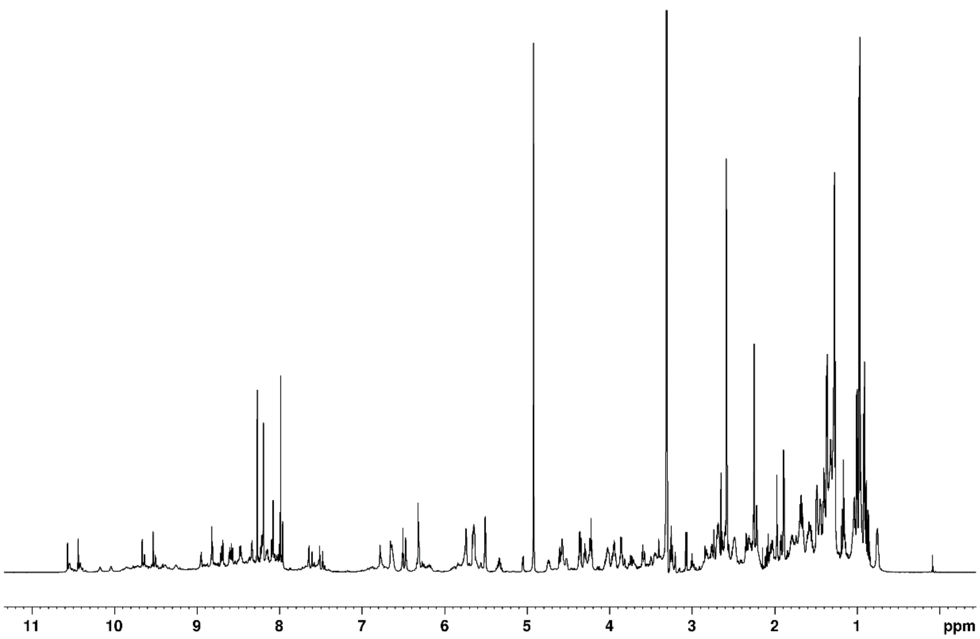

Figure S2: ^{1}H NMR spectrum of ^{15}N labeled NosA in CD_3OH.

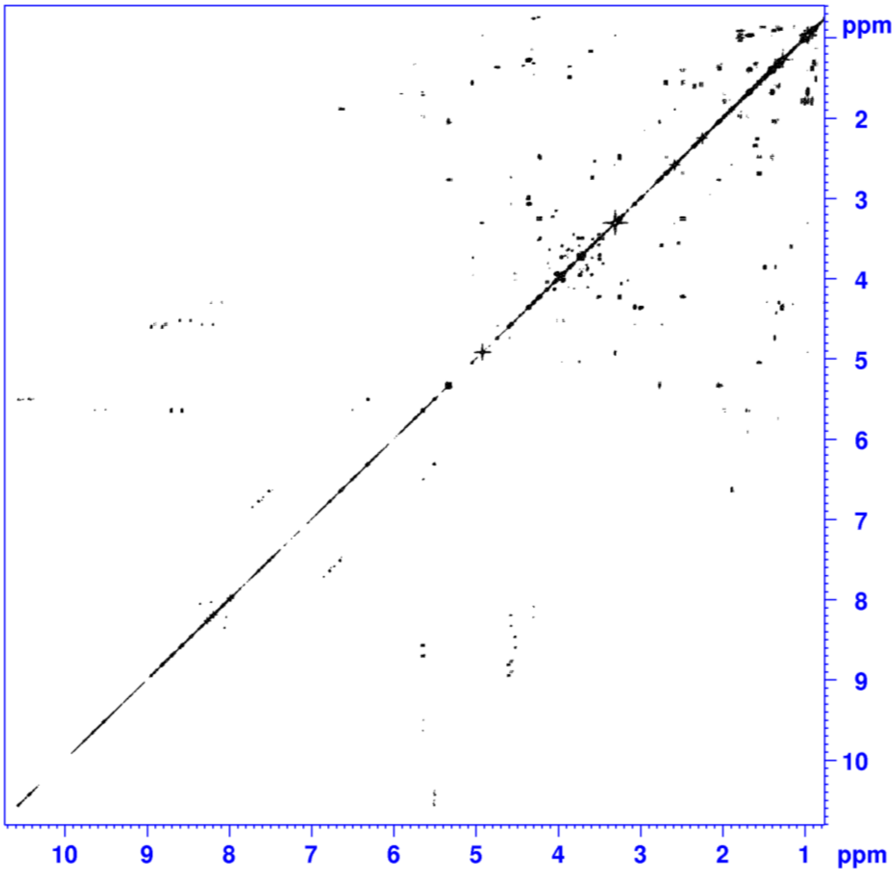

Figure S3: COSY spectrum of ^{15}N labeled NosA in CD_3OH.

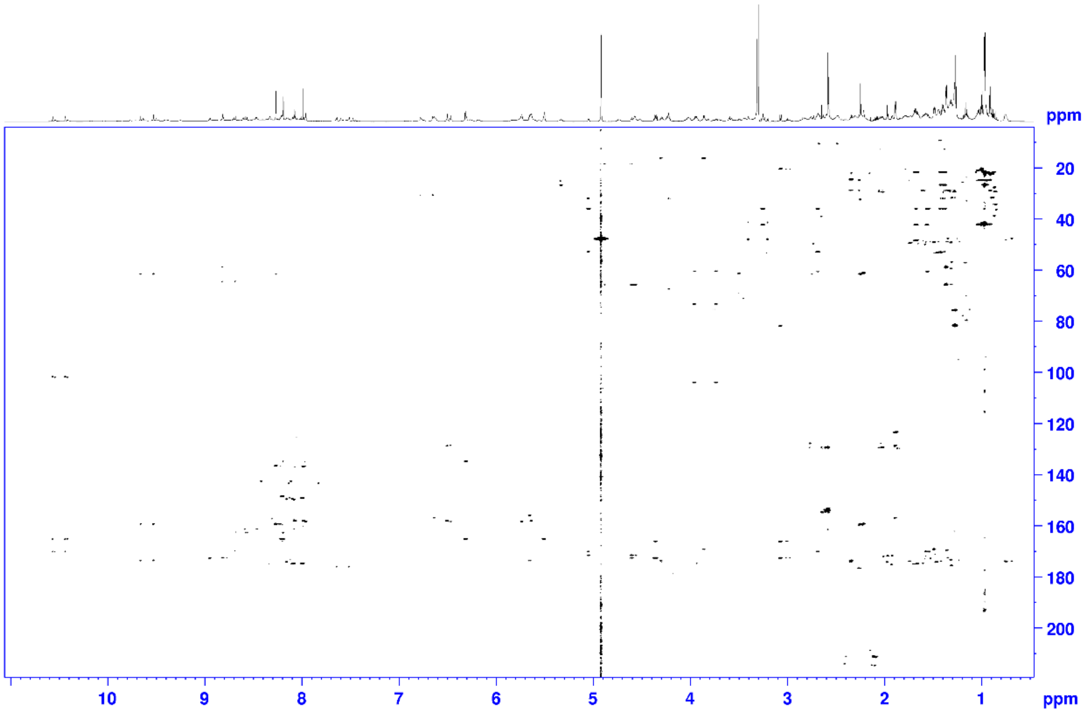

Figure S4: ^{1}H-^{13}C HMBC spectrum of ^{15}N labeled NosA in CD_3OH.

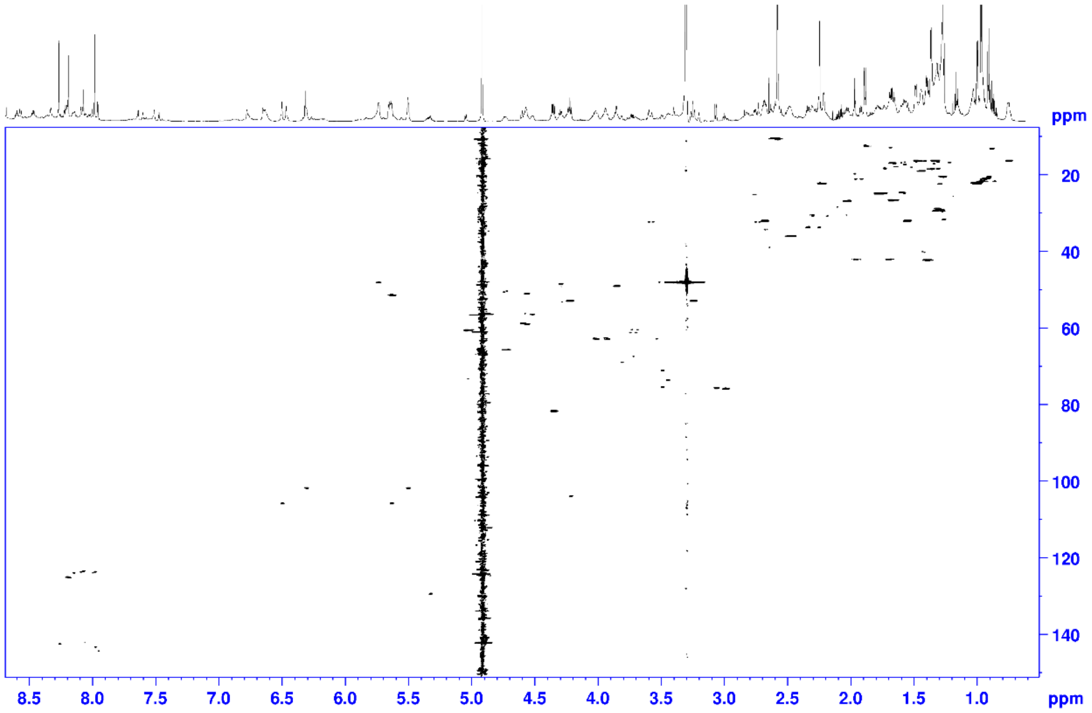

Figure S5: ^{1}H-^{13}C HSQC spectrum of ^{15}N labeled NosA in CD_3OH.

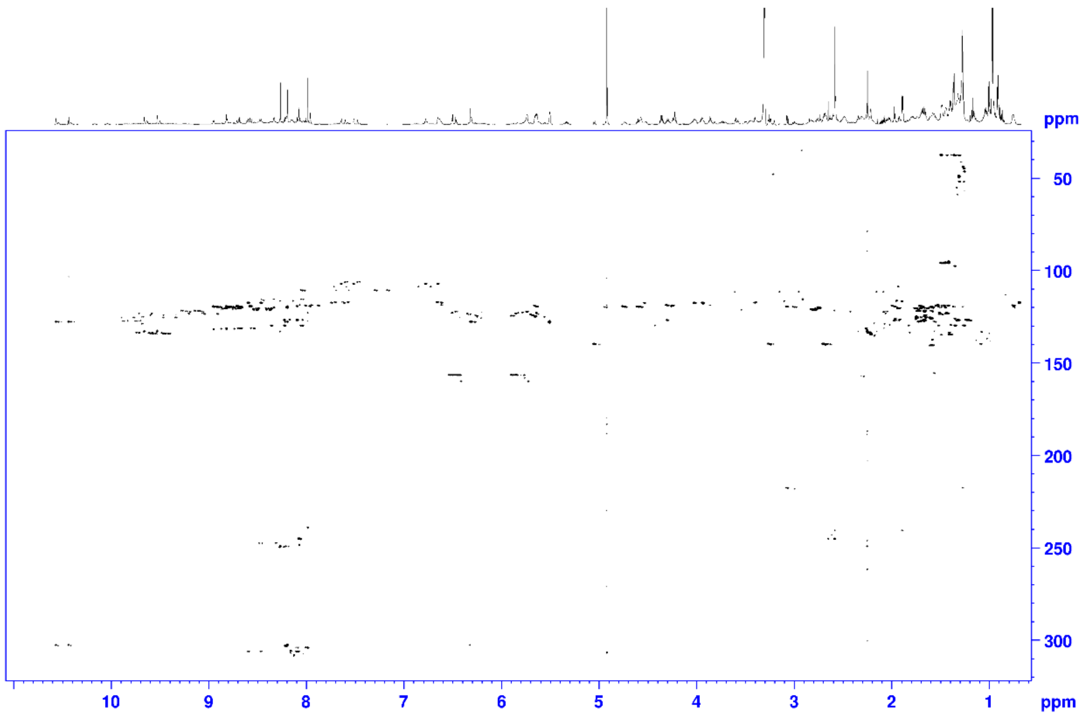

Figure S6: ^{1}H-^{15}N HMBC spectrum of ^{15}N labeled NosA in CD_3OH.

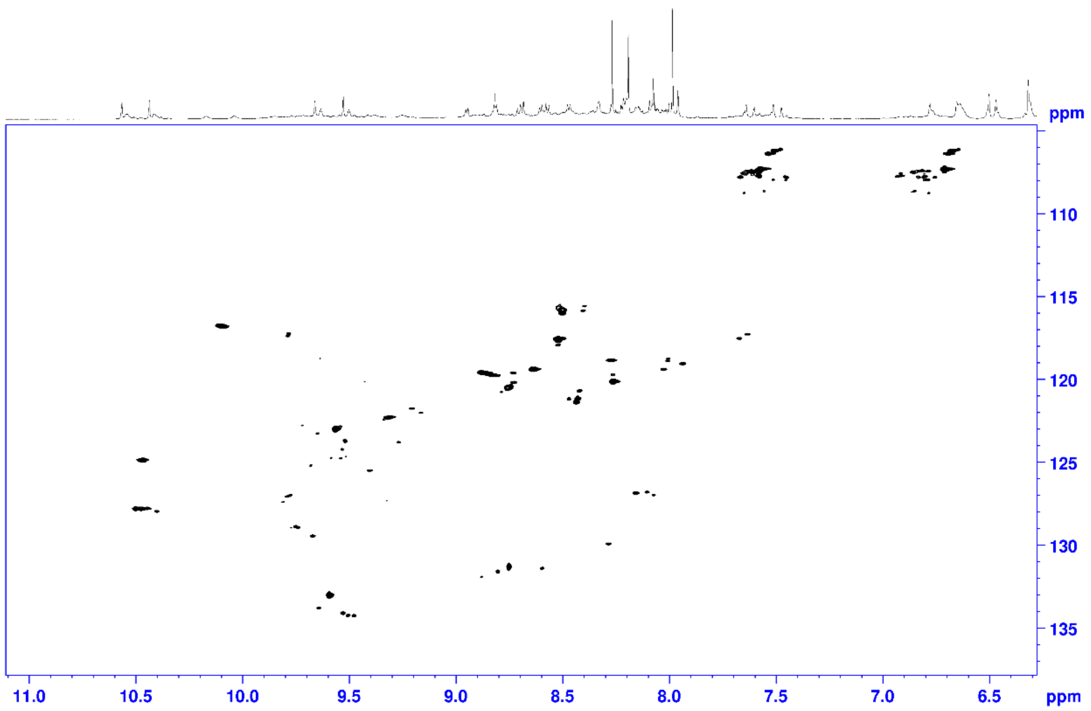

Figure S7: ^{1}H-^{15}N HSQC spectrum of ^{15}N labeled NosA in CD_3OH.

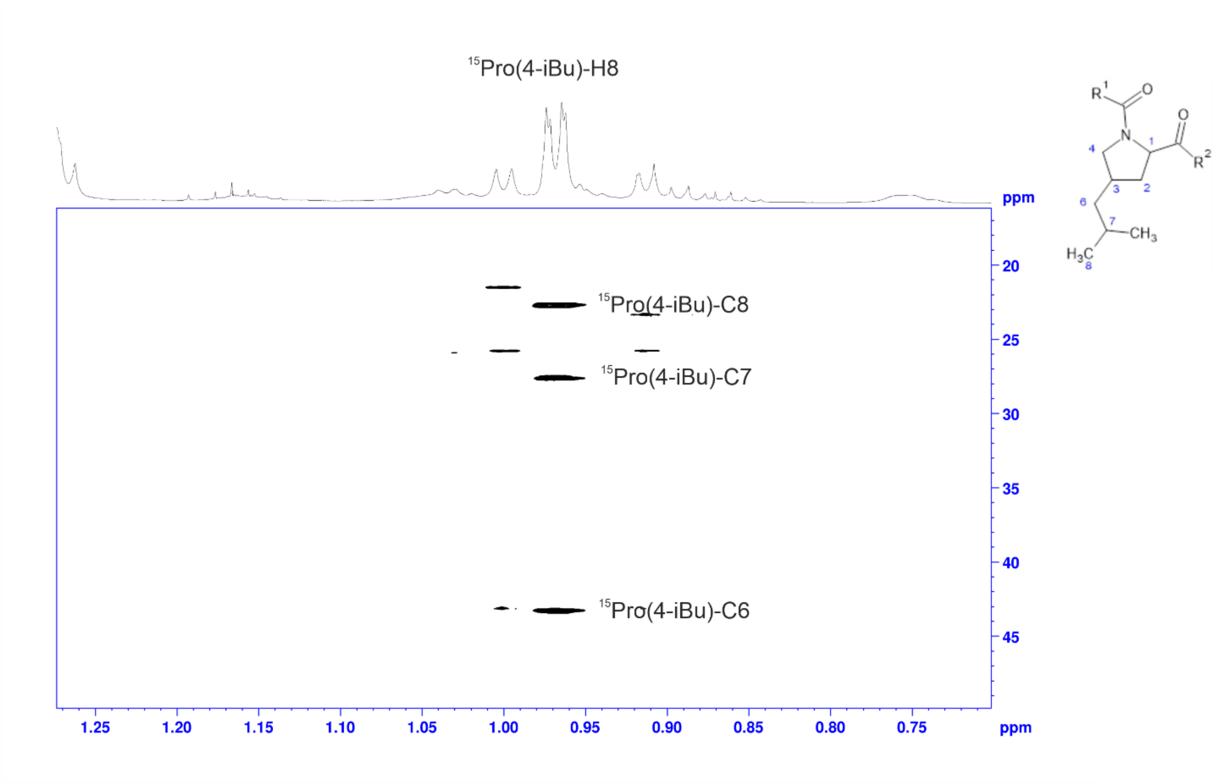

Figure S8: A detail of the Pro(4-iBu) ^{1}H-^{13}C HMBC spectrum

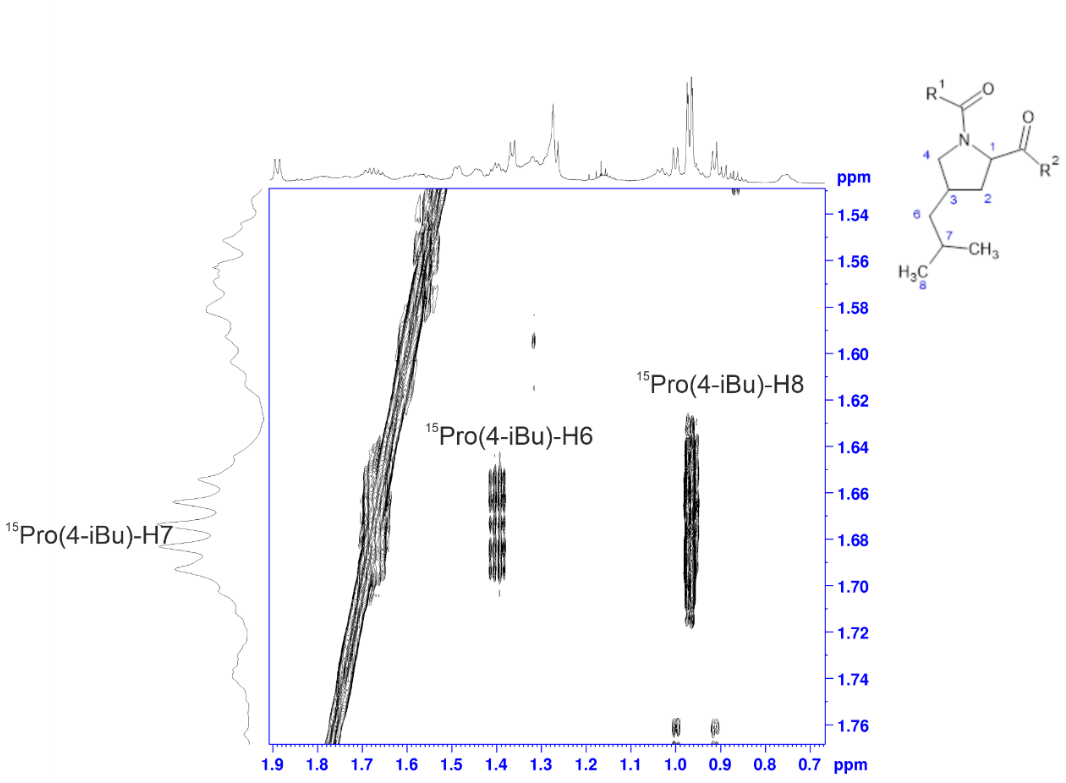

Figure S9: A detail of the Pro(4-iBu) COSY spectrum

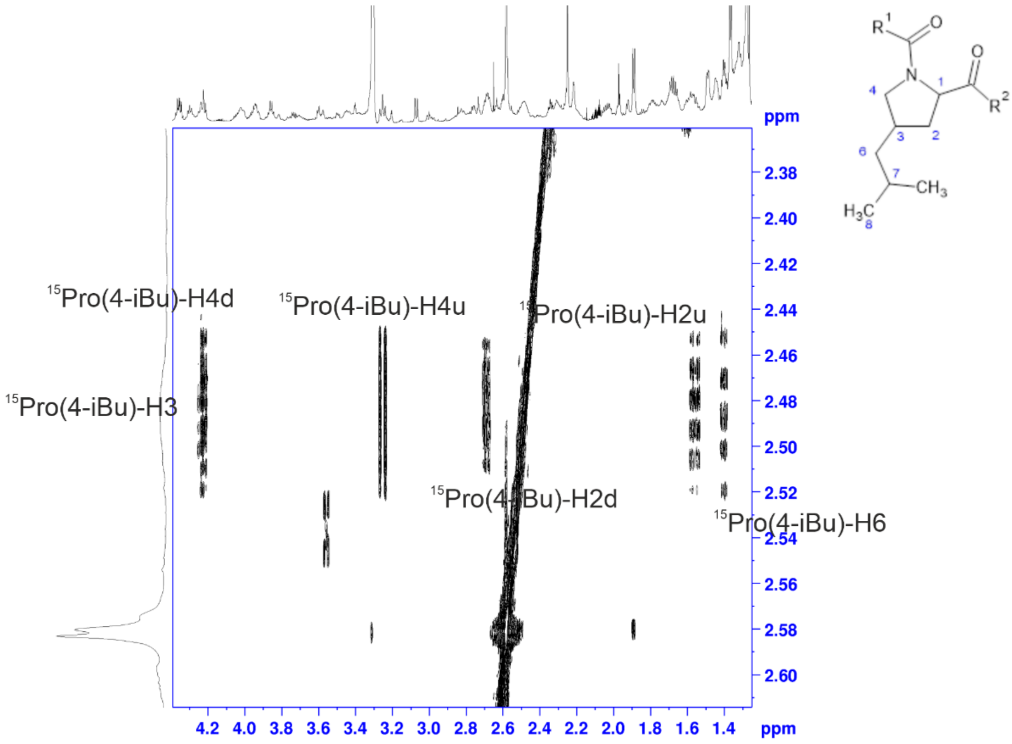

Figure S10: A detail of the Pro(4-iBu) COSY spectrum

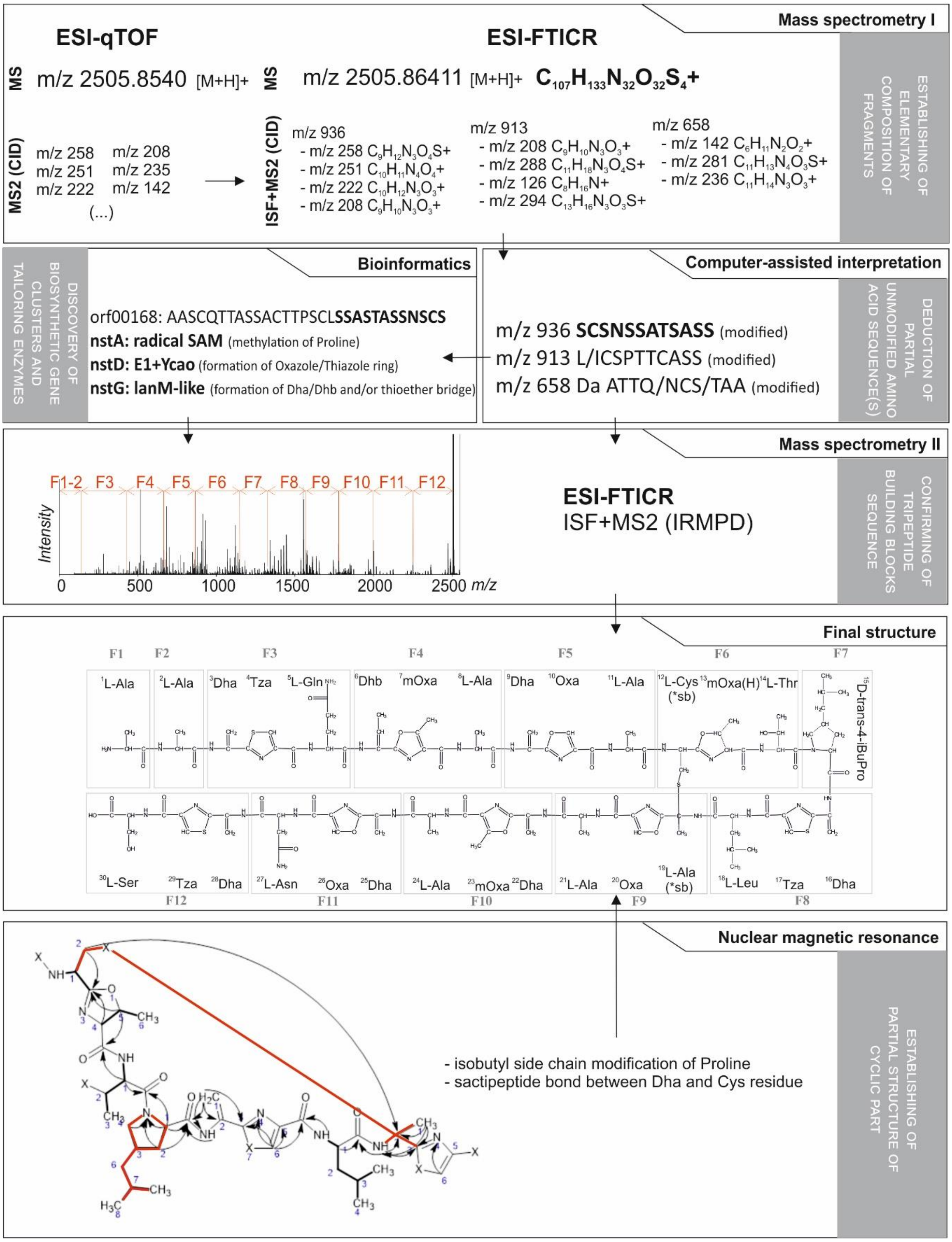

Figure S11: Schematic depiction of the structure elucidation process of nostatin A, the combination of different mass spectrometry methods and NMR is highlighted.

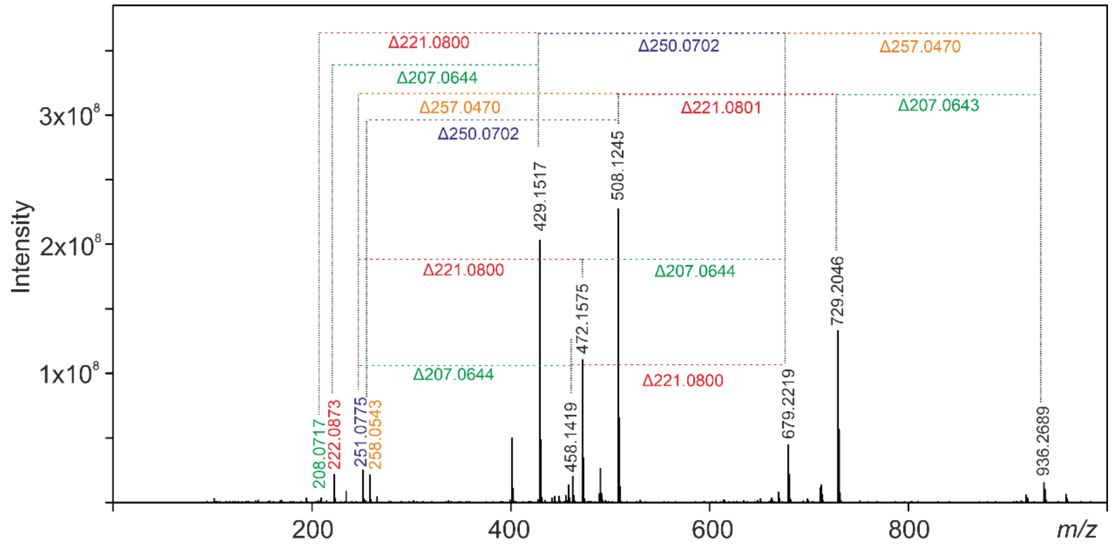

Figure S12: NosA low-molecular weight building blocks shown as neural losses and fragment ions of NosA daughter ion at *m/z* 936. Corresponding neutral loss and charged fragments are coded with identical color.

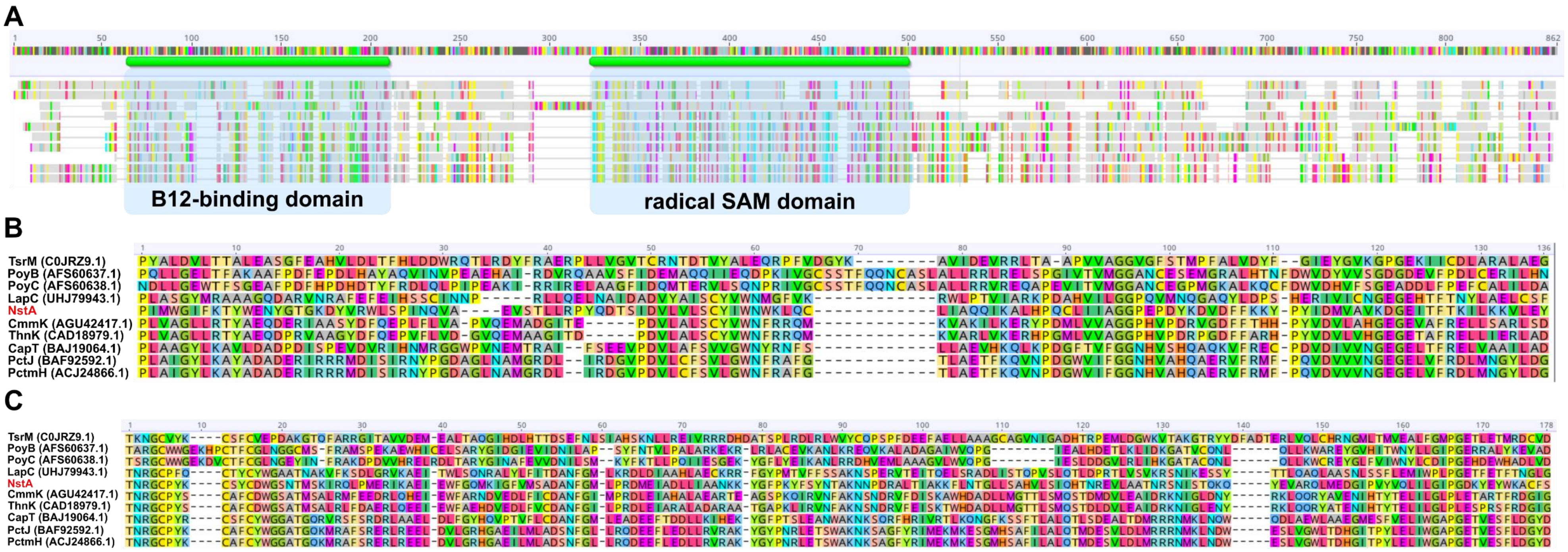

Figure S13: Protein alignment of the putative radical SAM C-methyltransferase NstA with selected known class B cobalamin-dependent C-methyltransferases. NstA is predicted to perform sequential C-methylation of the ^{15}Pro residue, iteratively forming the final isobutyl side chain. A) Overall view of the alignment showing the position of cobalamin (B12) binding domain and the radical SAM core domain. B) Detail of the alignment of the B12-binding domain. C) Detail of the alignment of the radical SAM core domain.

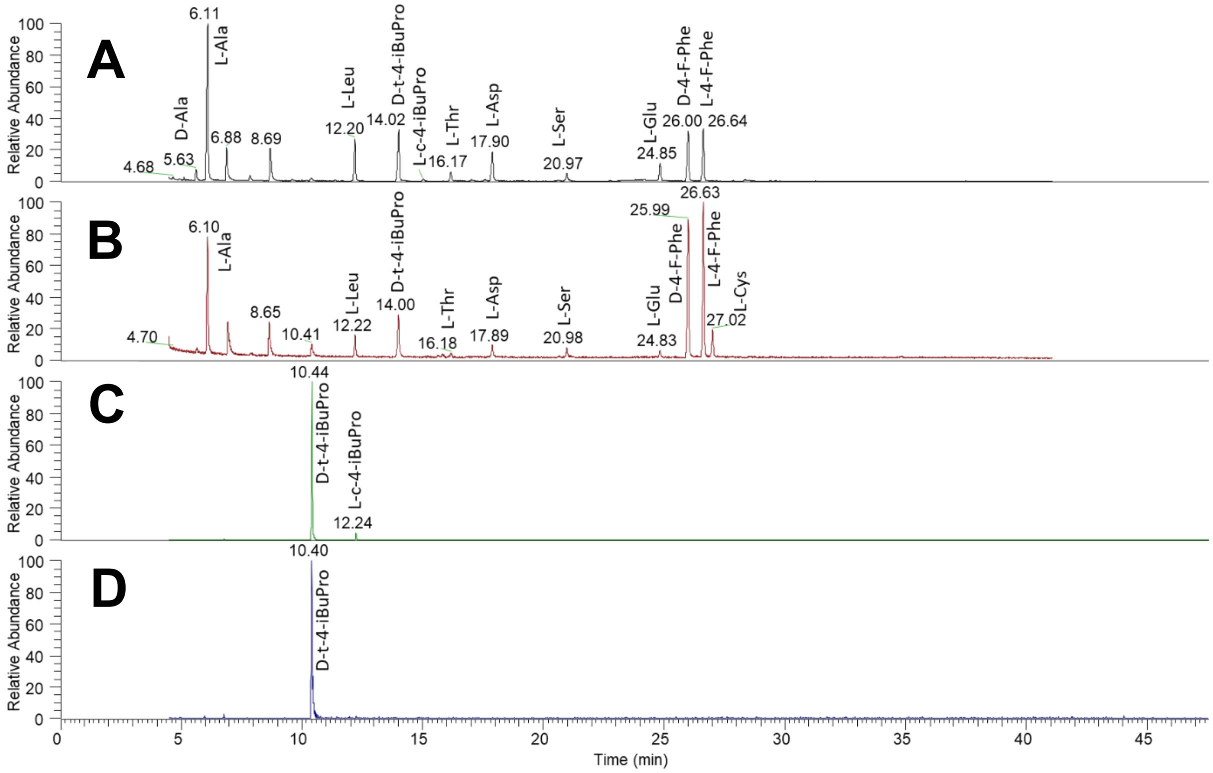

Figure S14: GC-MS chromatograms of chiral amino acid analysis after acidic hydrolysis of Nos A and derivatization of the hydrolysate. **A** – Total ion chromatogram of Nos A hydrolysate (115°C, 24h, 6M HCl) after HFBCF derivarization. **B** - Total ion chromatogram of Nos A hydrolysate at mild conditions (100°C, 2h, 5M HCl) after HFBCF derivarization. **C** - Extracted ion chromatogram (m/z 352.1 - D-t-4-iBuPro) of Nos A hydrolysate (115°C, 24h, 6M HCl) after HFBCF derivarization and amidation. **D** - Extracted ion chromatogram (m/z 352.1 - D-t-4-iBuPro) of Nos A hydrolysate at mild conditions (100°C, 2h, 5M HCl) after HFBCF derivarization and amidation. **4-F-Phe**= 4-fluorophenylalanine (IS); **D-t-4-iBuPro** = D-trans-4-isobutylproline; **L-c-4-iBuPro** = L-cis-4-isobutylproline.

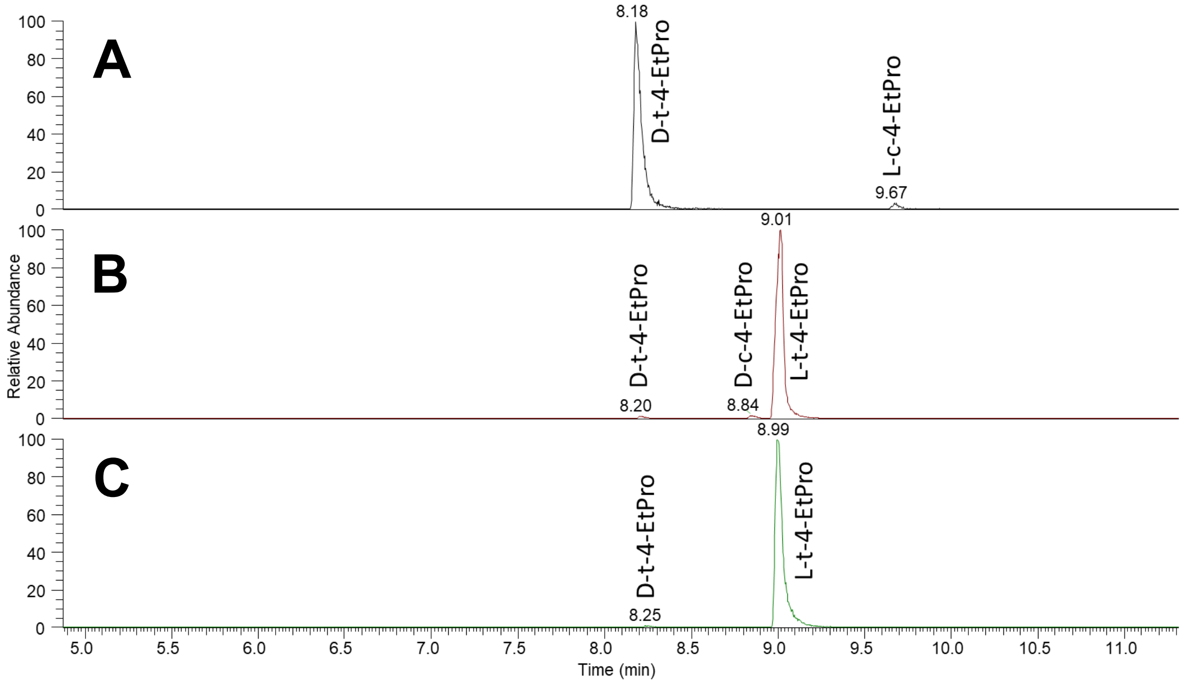

Figure S15: GC-MS chromatograms of chiral 4-ethylproline analysis after acidic hydrolysis of NosA analog 2477 and derivatization of the hydrolysate. **A -** Total ion chromatogram of Nos A analog 2477 hydrolysate (115°C, 24h, 6M HCl) after HFBCF derivarization and amidation. **B -** Extracted ion chromatogram (m/z 352.1 - D-t-4-EtPro bearing ^{15}N substitution) of 4-EtPro standard treated by hydrolytic conditions (115°C, 24h, 6M HCl) after HFBCF derivarization and amidation. **C -** Extracted ion chromatogram (m/z 352.1 - D-t-4-EtPro bearing ^{15}N substitution) of 4-EtPro standard after HFBCF derivarization and amidation. D-t-4-EtPro = D-trans-4-ethylproline; D-c-4-EtPro = D-cis-4-ethylproline; L-t-4-EtPro = L-trans-4-ethylproline; L-c-4-EtPro = L-cis-4-ethylproline.

A

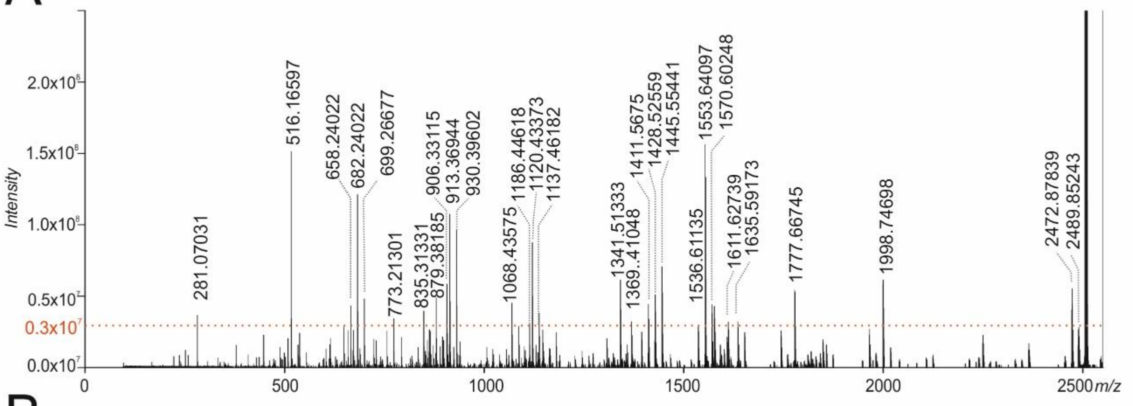

B

fragment	summ formula	theoretical mass	measured mass	ppm
F3	$C_{11}H_{13}N_4O_3S_1$+	281.07029	281.07031	0.08
F3+F4	$C_{22}H_{26}N_7O_6S_1$+	516.16598	516.16597	-0.02
F1+F2+F3+F4	$C_{28}H_{36}N_9O_8S_1$+	658.24021	658.24022	0.02
F3+F4+*166* (not assigned)	$C_{30}H_{36}N_9O_8S$+	682.24021	682.24022	-0.01
F3+F4+*166* +NH3 (not assigned)	$C_{30}H_{39}N_{10}O_8S$+	699.26676	699.26677	0.09
F3+F4+F12 (intramolecular cyclisation after fragmentation)	$C_{31}H_{37}N_{10}O_{10}S_2$+	773.21301	773.21301	0
[M+3H]3+ (triple charged mother ion, deformation due to isolation window)			835.31331	
F5+F6+F7+F8-SH2	$C_{41}H_{55}N_{10}O_{10}S_1$+	879.38179	879.38185	0.07
F3+F4+F5+*166* +NH3 (not assigned)	$C_{39}H_{48}N_{13}O_{11}S$+	906.33118	906.33115	-0.04
F5+F6+F7+F8	$C_{41}H_{57}N_{10}O_{10}S_2$+	913.36951	913.36948	-0.03
F5+F6+F7+F8+NH3	$C_{41}H_{60}N_{11}O_{10}S_2$+	930.39606	930.39602	-0.38
F5+F6+F7+F8+F9-SH2-H2O	$C_{50}H_{62}N_{13}O_{12}S$+	1068.43561	1068.43575	-0.13
F5+F6+F7+F8+F9-SH2	$C_{50}H_{64}N_{13}O_{13}S$+	1086.44618	1086.44617	0.01
F5+F6+F7+F8+F9	$C_{50}H_{66}N_{13}O_{13}S_2$+	1120.4339	1120.43373	0.15
F5+F6+F7+F8+F9+NH3	$C_{50}H_{69}N_{14}O_{13}S_2$+	1137.46045	1137.46039	0.05
F5+F6+F7+F8+F9+F10	$C_{60}H_{77}N_{16}O_{16}S_2$+	1341.51394	1341.51333	0.45
not assigned			1369.41048	
F3+F4+F5+F6+F7+F8-SH2	$C_{63}H_{83}N_{18}O_{16}S_2$+	1411.56704	1411.5671	-0.05
F3+F4+F5+F6+F7+F8	$C_{63}H_{82}N_{17}O_{16}S_3$+	1428.52821	1428.52559	-1.84
F3+F4+F5+F6+F7+F8+NH3	$C_{63}H_{85}N_{18}O_{16}S_3$+	1445.55476	1445.55441	-0.16
F1+F2+F3+F4+F5+F6+F7+F8-SH2	$C_{69}H_{90}N_{19}O_{18}S_2$+	1536.61472	1536.61316	1.01
F1+F2+F3+F4+F5+F6+F7+F8-SH2+NH3	$C_{69}H_{93}N_{20}O_{18}S_2$+	1553.64127	1553.64097	0.19
F1+F2+F3+F4+F5+F6+F7+F8	$C_{69}H_{92}N_{19}O_{18}S_3$+	1570.60244	1570.60248	0.03
F3+F4+F5+F6+F7+F8+NH3+*166* (not assi	$C_{17}H_{95}N_{20}O_{18}S_3$+	1611.62899	1611.62739	0.1
F3+F4+F5+F6+F7+F8+F9	$C_{72}H_{91}N_{20}O_{19}S_3$+	1635.5926	1635.59173	-0.53
F1+F2+F3+F4+F5+F6+F7+F8+F9	$C_{78}H_{101}N_{22}O_{21}S_3$+	1777.66683	1777.66782	0.56
F1+F2+F3+F4+F5+F6+F7+F8+F9+F10	$C_{88}H_{112}N_{25}O_{24}S_3$+	1998.74687	1998.74698	0.05
M-SH2	$C_{107}H_{131}N_{32}O_{32}S_3$+	2471.87639	2471.87776	0.56
M-H2O	$C_{107}H_{131}N_{32}O_{31}S_4$+	2487.85354	2487.85315	-0.16

Figure S16: Interpretation of the most intense MS fragments of Nostatin A obtained using IRMPD-MS method. Original mass spectrum highlighting m/z values of the most intense ions (A) and the interpretation of particular fragment ions, their (tri-)peptide building block composition, exact mass and error values (B) are show. The intensity threshold was set to 0.3 x 10^7. We were not able to fully interpret ions containing neutral loss 166 ($C_8H_{10}N_2O_2$) originating from $M+H^+$, we expect its occurrence via intramolecular cyclization after fragmentation.

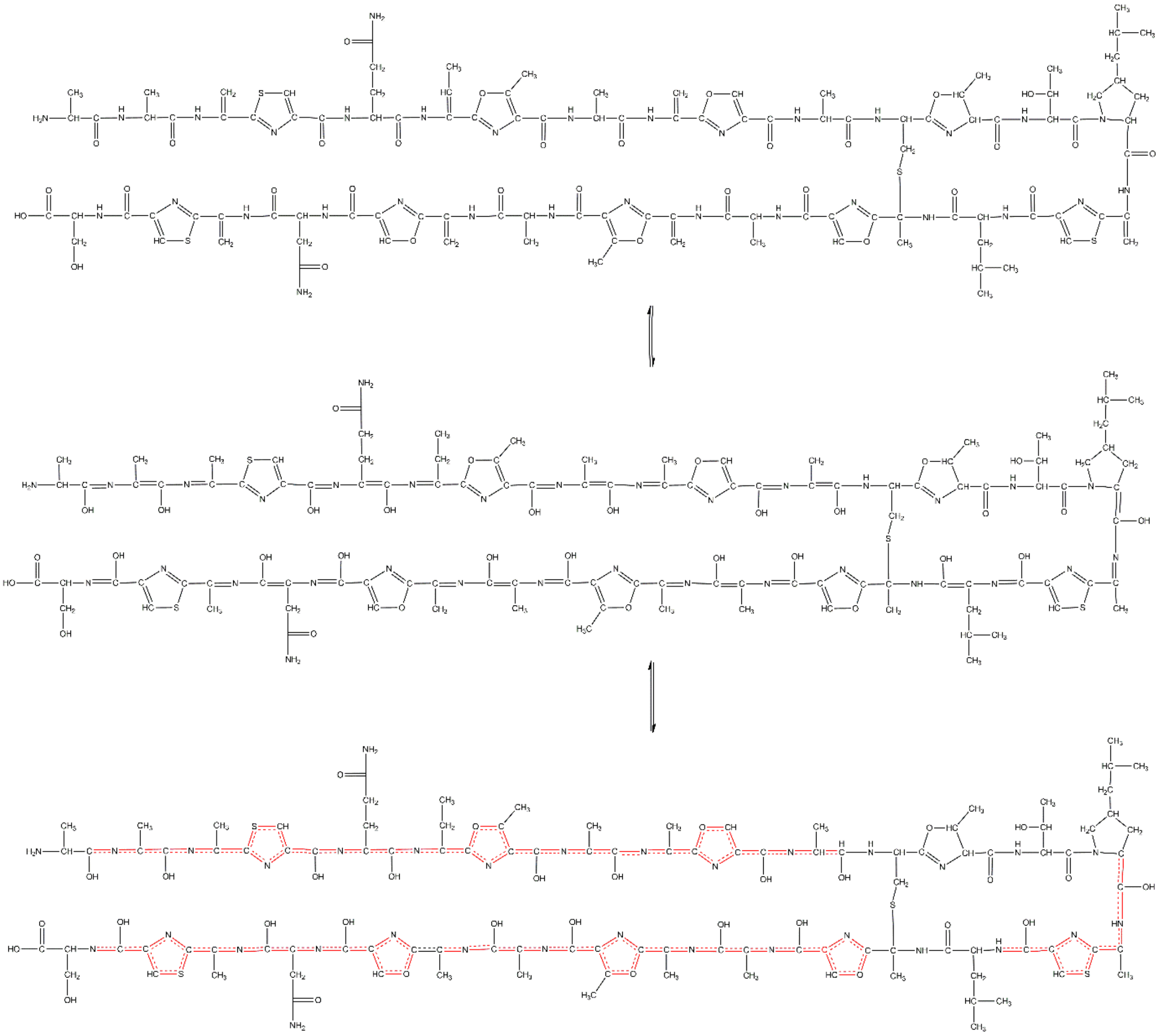

Figure S17: Suggested tentative NosA tautomeric structure. Due to the high number of aromatic heterocycles and Dha/Dhb residues, NosA might form a tautomeric structure, leading to the reorganization of hydrogens present on the main backbone, which is likely enabled by a shift of the equilibrium of the peptide bond in favor of the iminol form and relocalization of double bonds present in Dha/Dhb from exomethylene to imine. In the case of natural amino acids adjacent to Dha/Dhb, we expect the formation of the enol form to contribute to the delocalization. This tautomeric structure is expected to be more favored and stabilized by the formation of two large, fully delocalized systems.

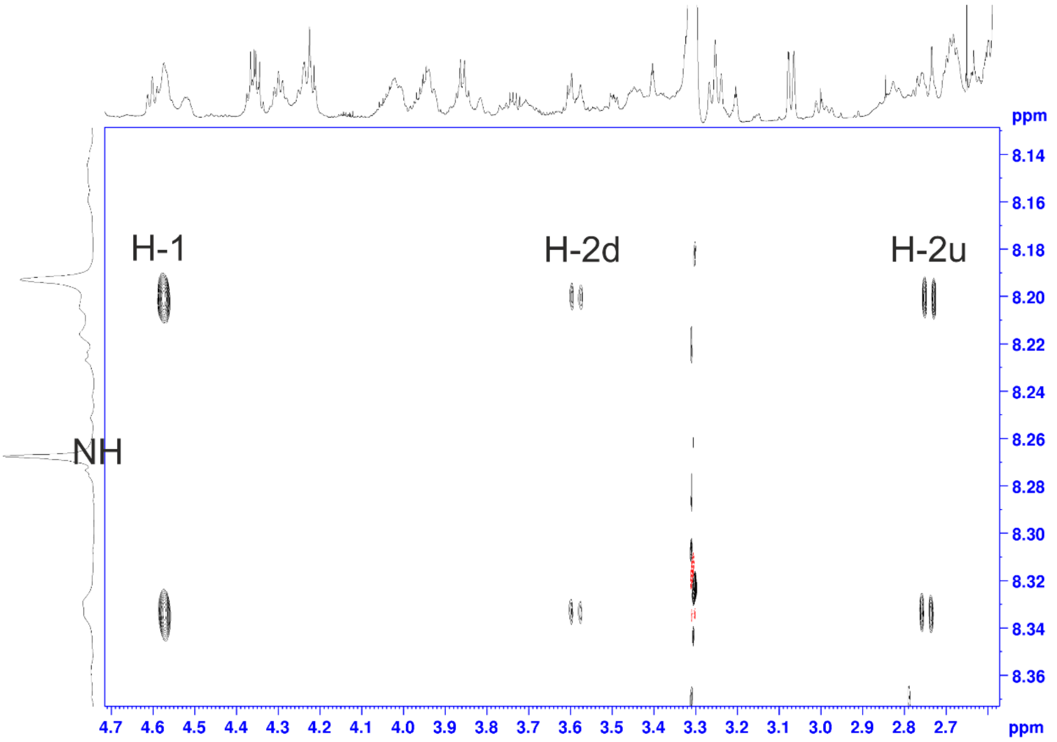

Figure S18: The TOCSY spectrum of the residue ^{12}Cys of ^{15}N labeled NosA in CD_3OH

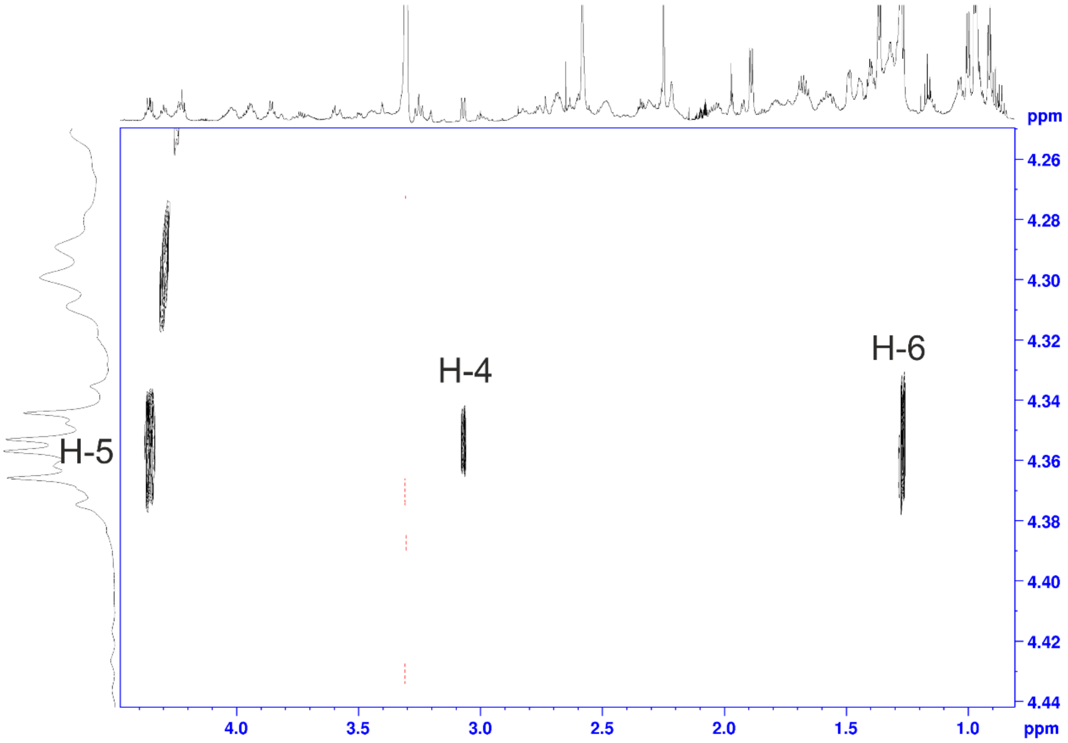

Figure S19: The TOCSY spectrum of the residue ^{13}mOxa(H) of ^{15}N labeled NosA in CD_3OH

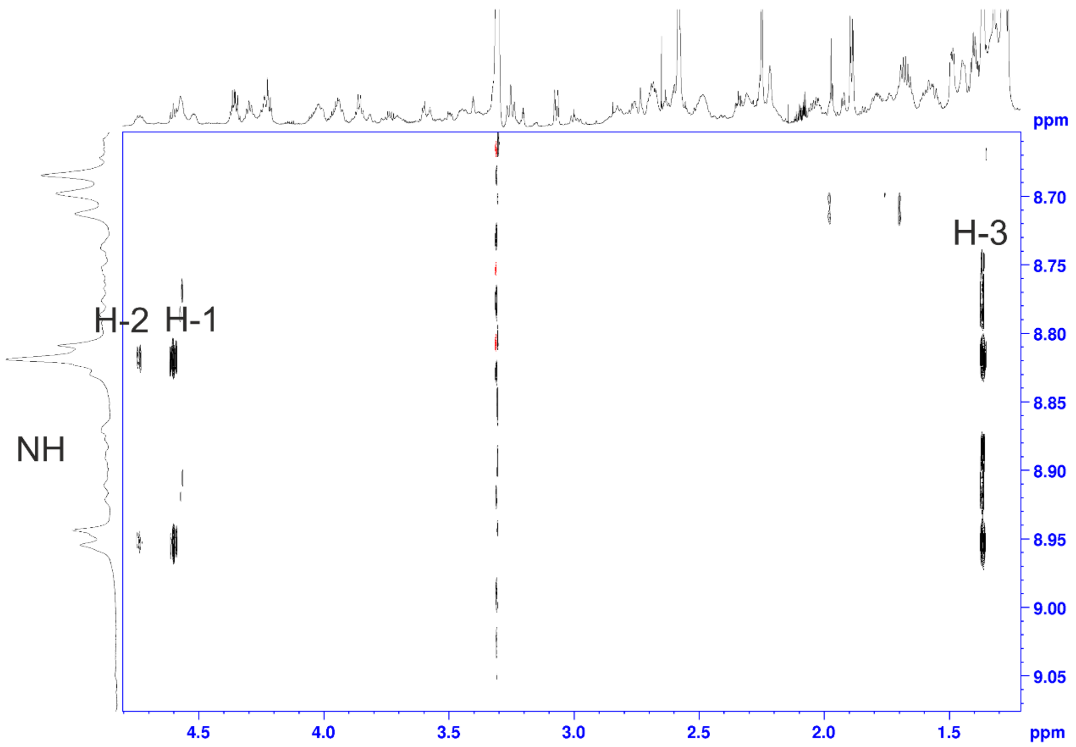

Figure S20: The TOCSY spectrum of the residue [14]Thr of ^{15}N labeled NosA in CD_3OH

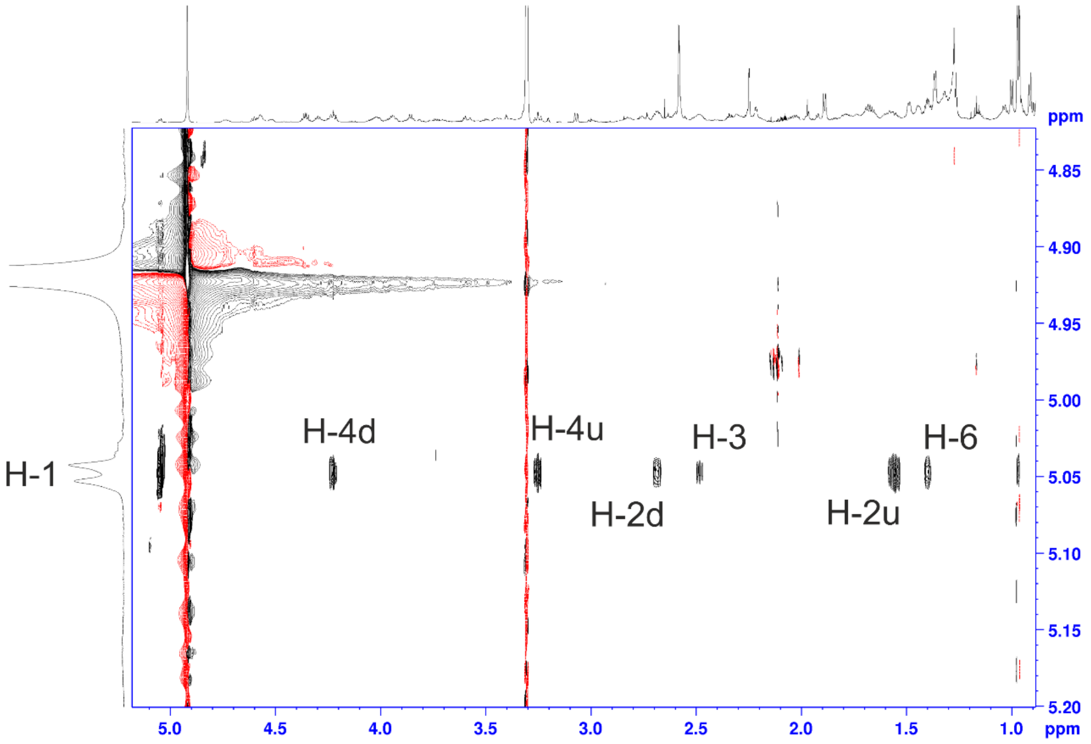

Figure S21: The TOCSY spectrum of the residue [15]Pro (4-iBu) of ^{15}N labeled NosA in CD_3OH

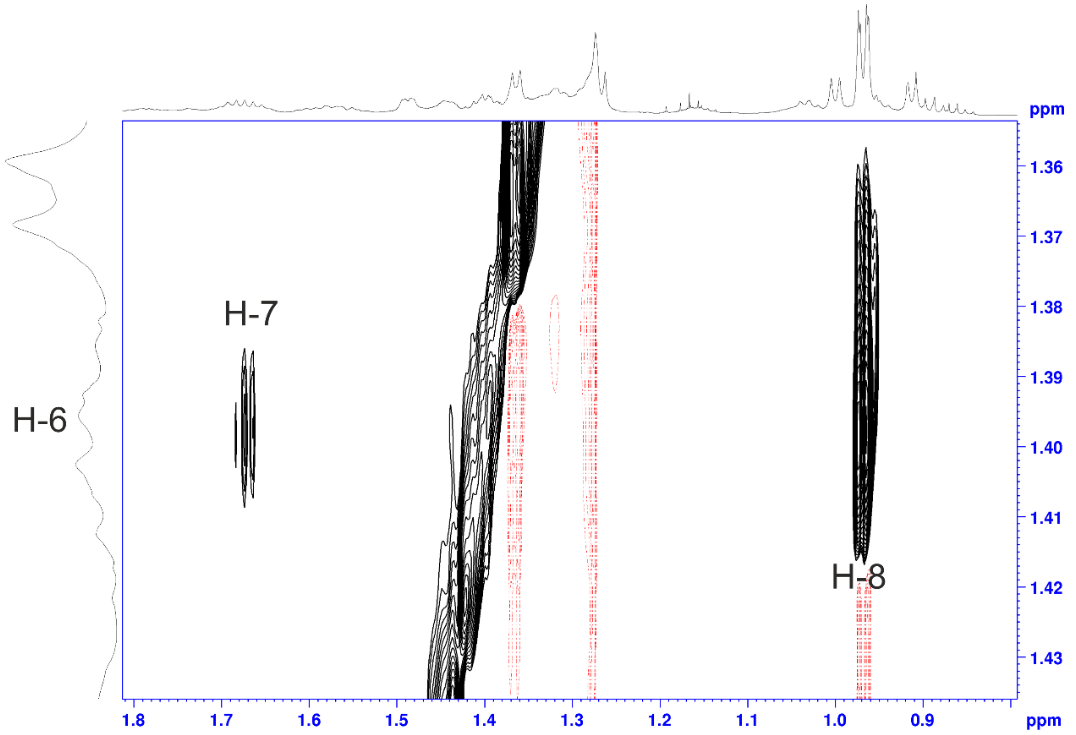

Figure S22: The TOCSY spectrum of the residue [15]Pro (4-iBu) of ^{15}N labeled NosA in CD_3OH – the side chain

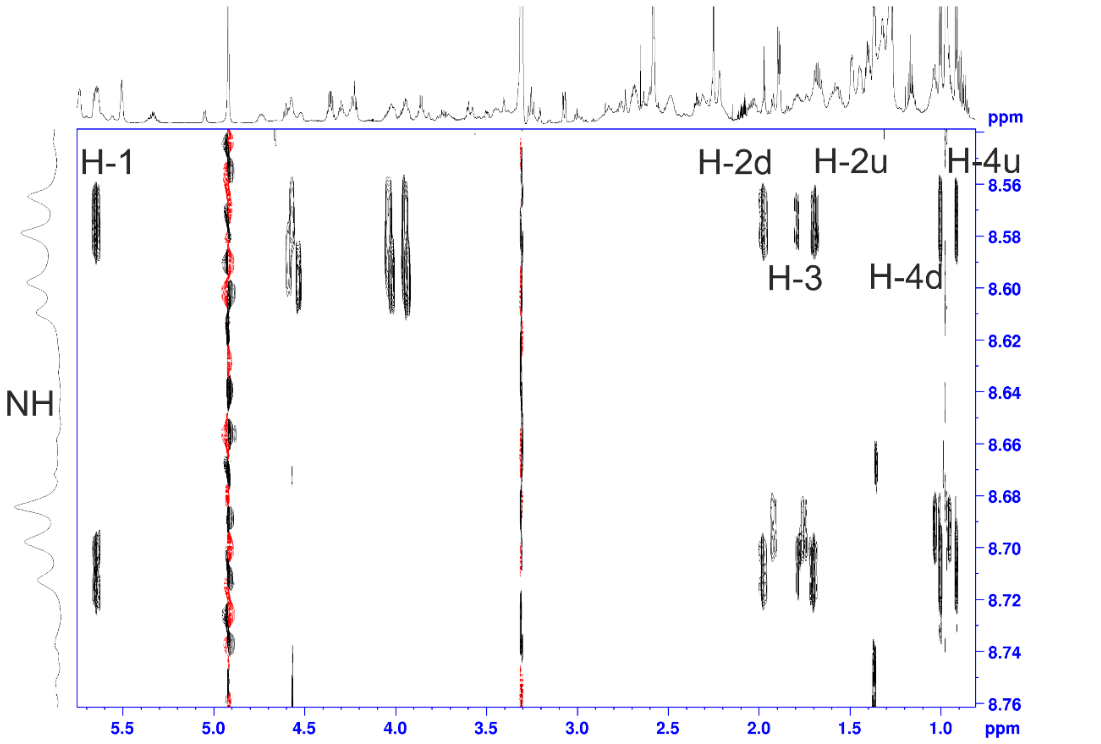

Figure S23: The TOCSY spectrum of the residue [18]Leu of ^{15}N labeled NosA in CD_3OH

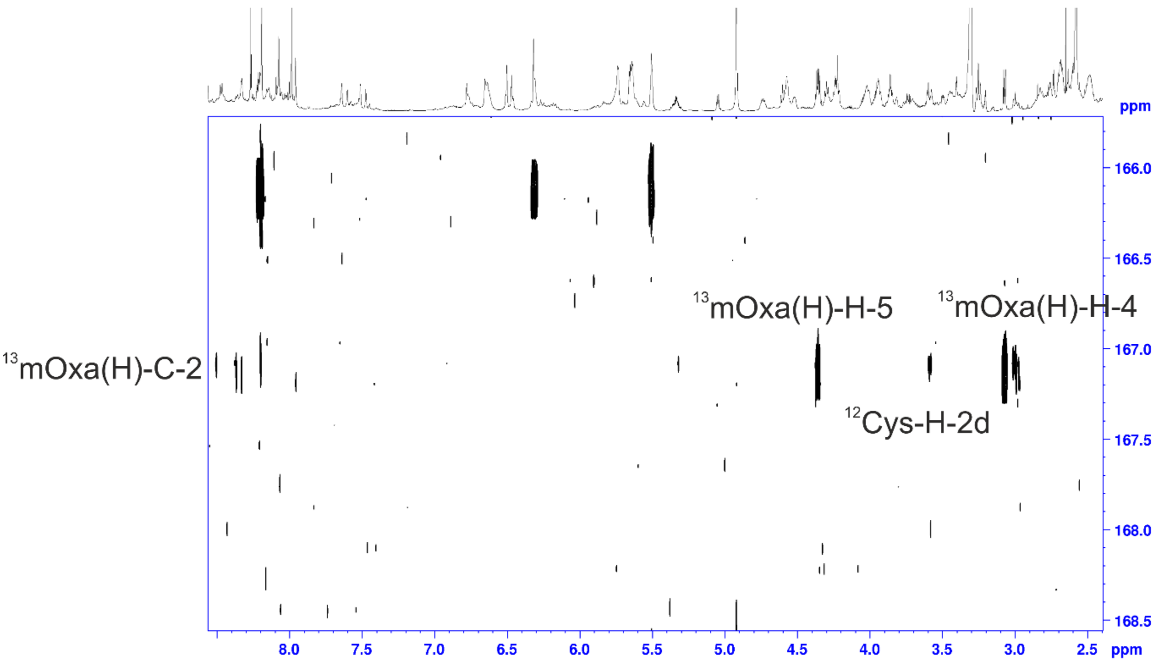

Figure S24: The ^{1}H-^{13}C HMBC correlation to carbon ^{13}mOxa(H)-C-2 of ^{15}N labeled NosA in CD_3OH

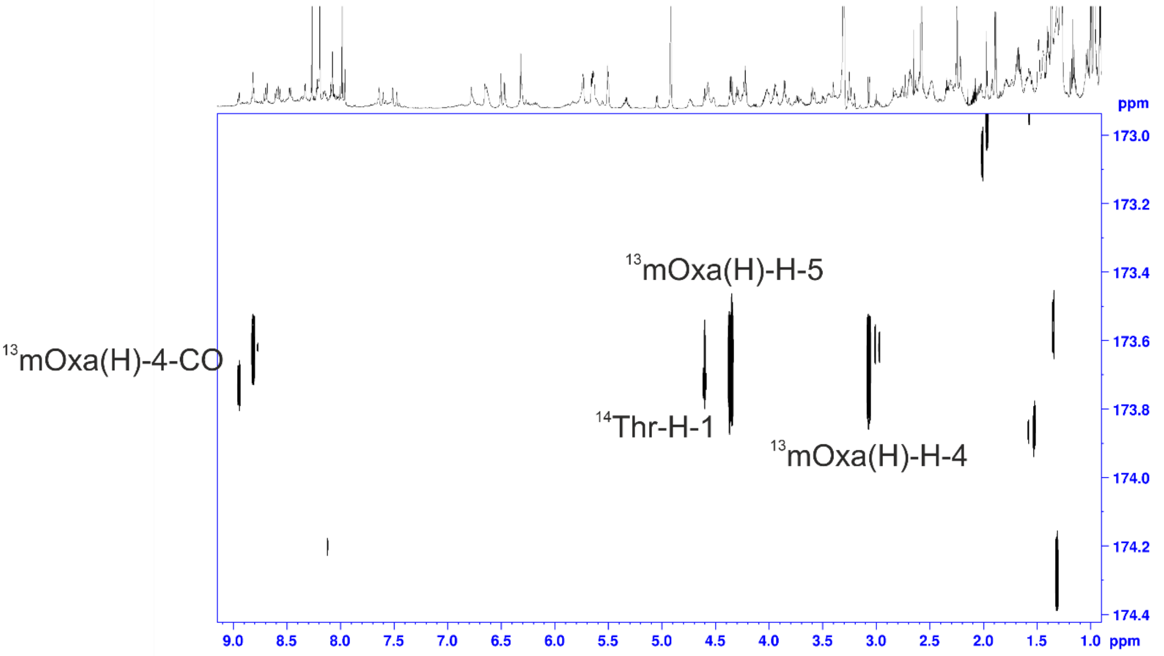

Figure S25: The ^{1}H-^{13}C HMBC correlation to carbon ^{13}mOxa(H)-4-CO of ^{15}N labeled NosA in CD_3OH

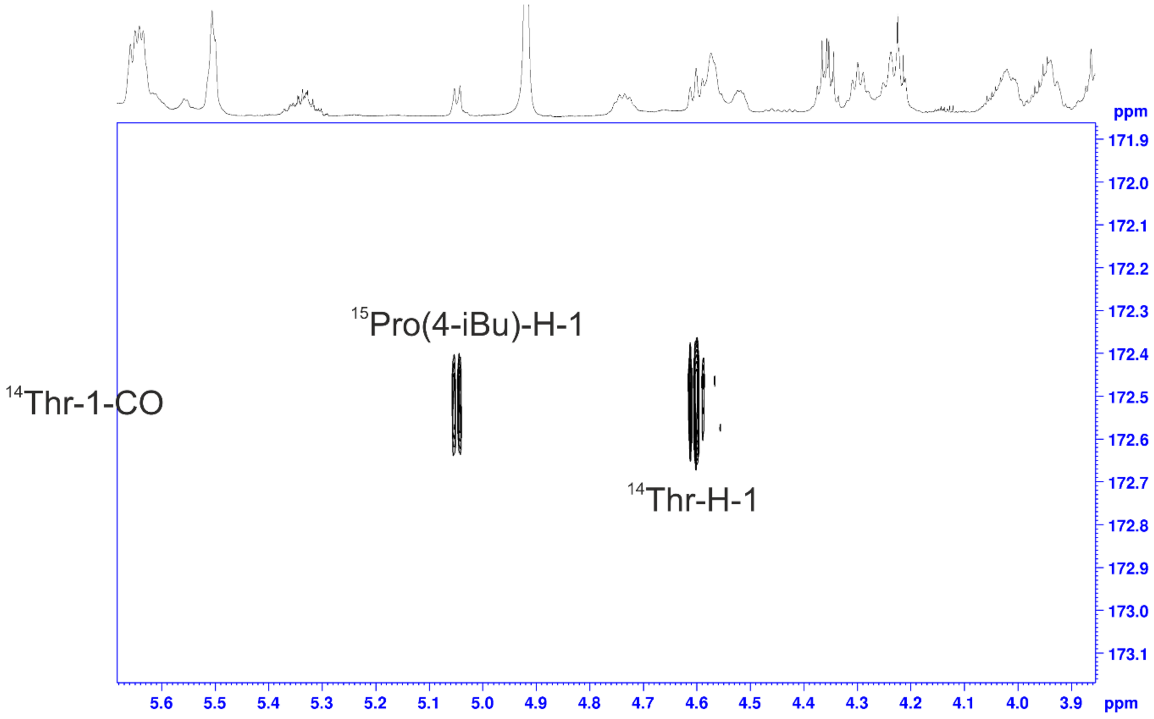

Figure S26: The ^{1}H-^{13}C HMBC correlation to carbon ^{14}Thr-1-CO of ^{15}N labeled NosA in CD_3OH

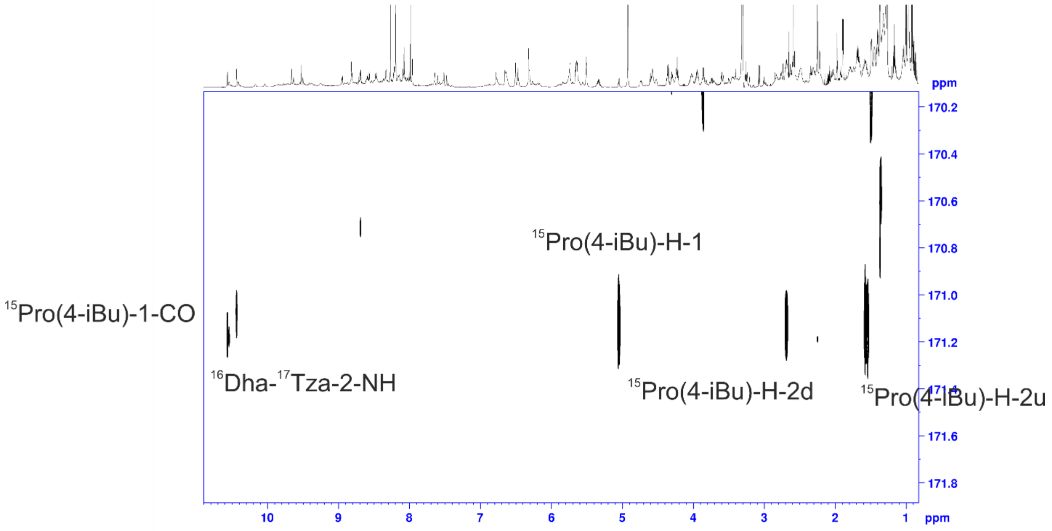

Figure S27: The ^{1}H-^{13}C HMBC correlation to carbon ^{15}Pro(4-iBu)-1-CO of ^{15}N labeled NosA in CD_3OH

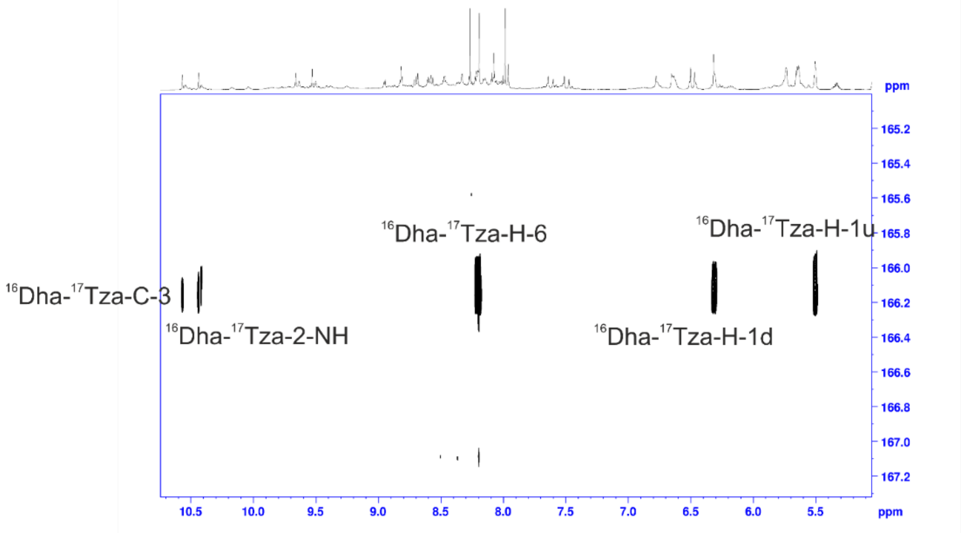

Figure 28: The ^{1}H-^{13}C HMBC correlation to carbon ^{16}Dha-^{17}Tza-C-3 of ^{15}N labeled NosA in CD_3OH

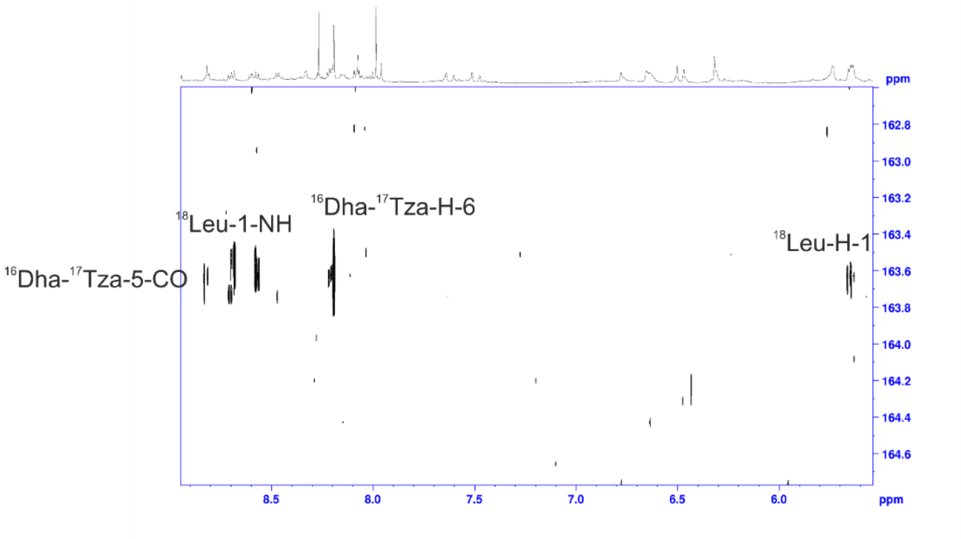

Figure 29: The ^{1}H-^{13}C HMBC correlation to carbon ^{16}Dha-^{17}Tza-5-CO of ^{15}N labeled NosA in CD_3OH

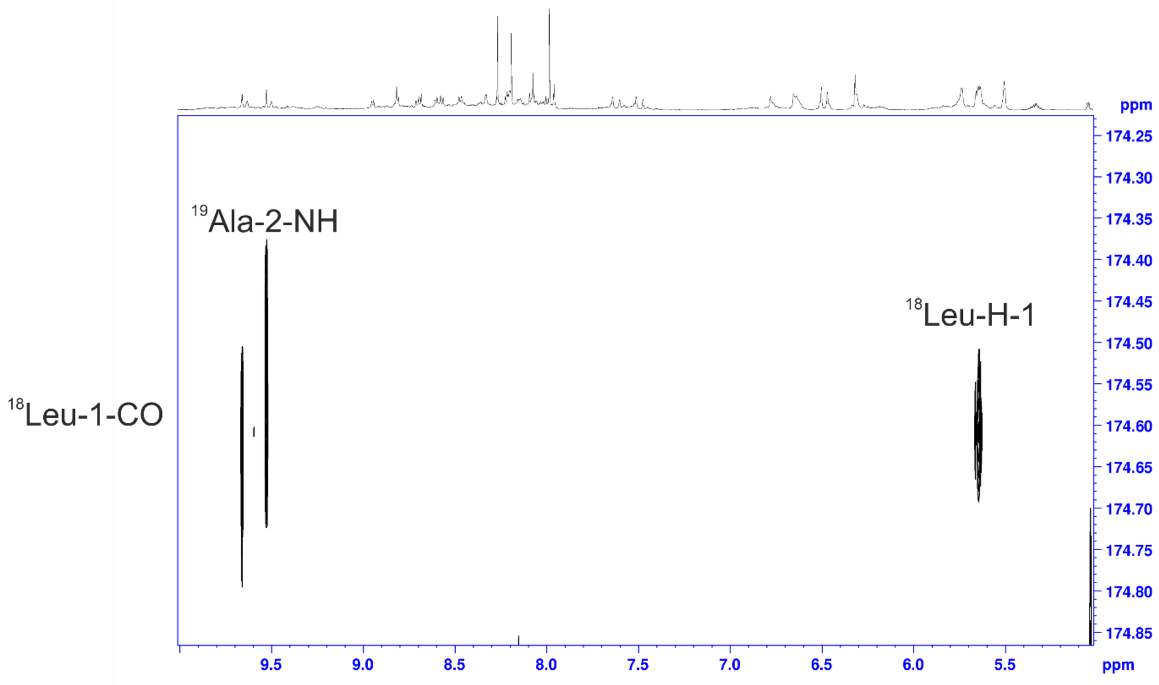

Figure 30: The ^{1}H-^{13}C HMBC correlation to carbon ^{18}Leu-1-CO of ^{15}N labeled NosA in CD_3OH

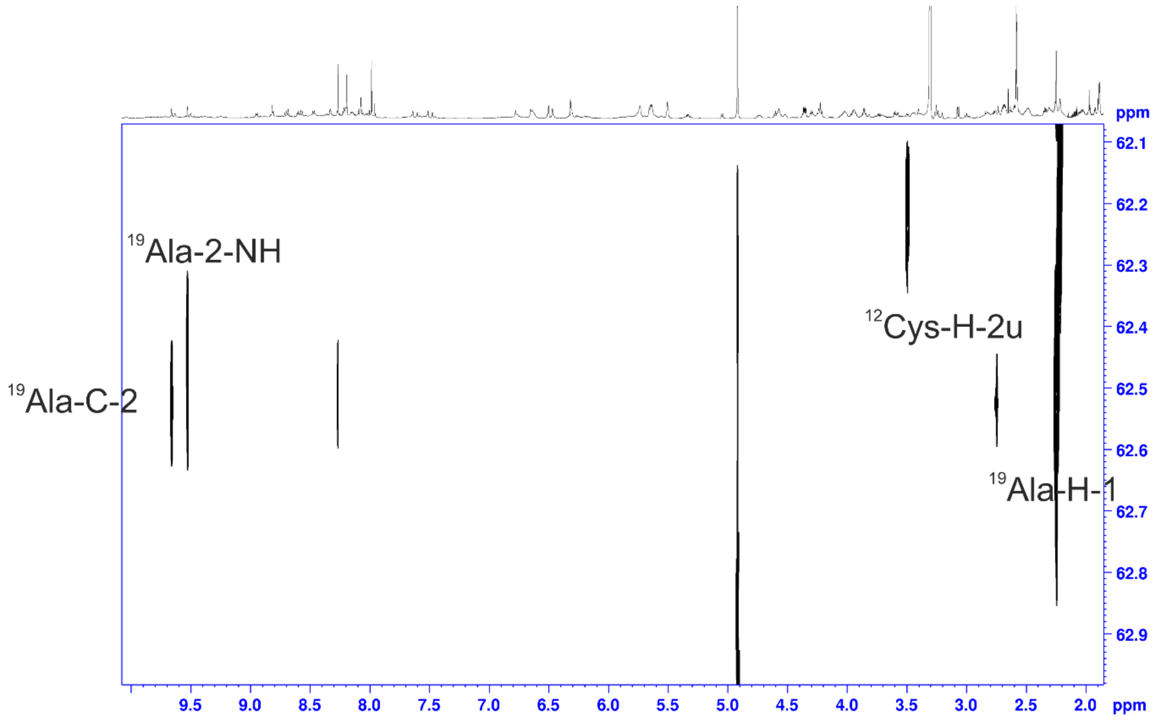

Figure 31: The ^{1}H-^{13}C HMBC correlation to carbon ^{19}Ala-C-2 of ^{15}N labeled NosA in CD_3OH

..

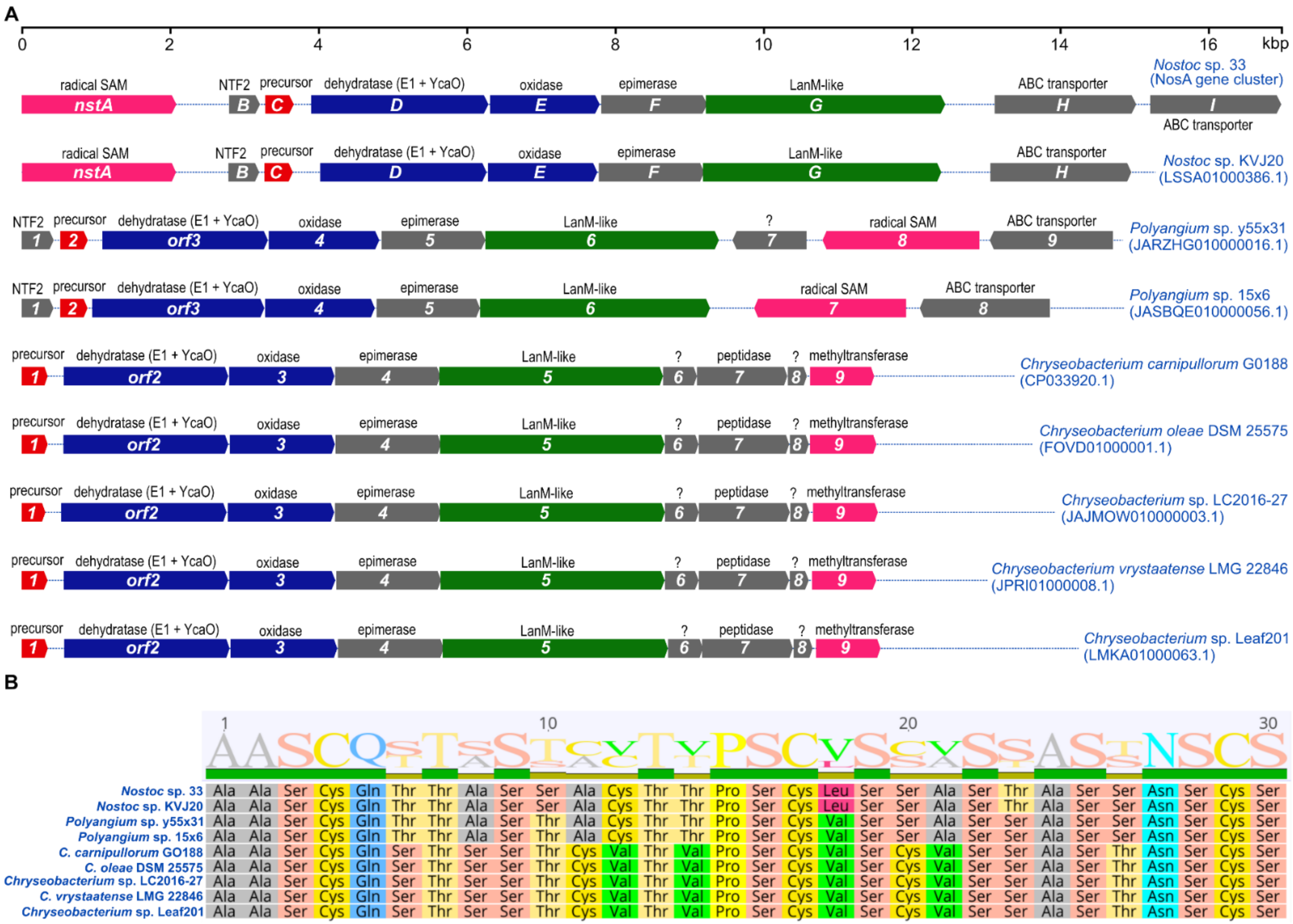

Figure S32: Comparison of the NosA biosynthetic gene cluster to homologous predicted gene clusters identified in publicly available bacterial genomes. A) Gene map comparing the topology and functional annotation of genes in the selected BGCs, showing a similar set of encoded tailoring enzymes. B) Alignment of the primary core peptide sequences of putative NosA analogues. The ^{15}Pro residue is conserved in all depicted BGCs.

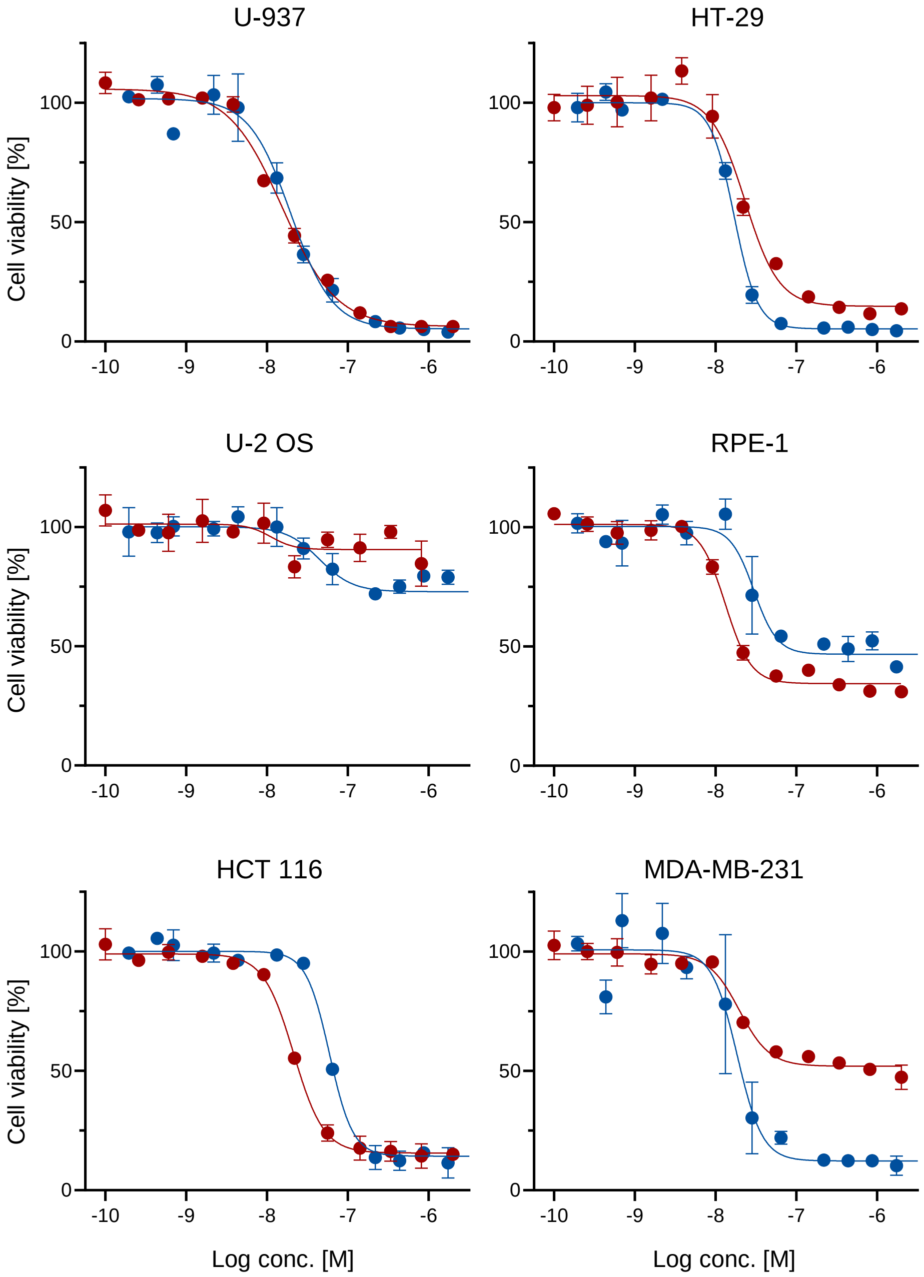

Figure S33: Cell viability titration experiments of NosA in the normal-immortalized (RPE-1) and selected cancer cell lines: U-937 (lymphoma), HT-29 and HCT 116 (colon adenocarcinoma), U-2 OS (osteosarcoma), and MDA-MB-231 (mammary gland adenocarcinoma). Cells were incubated with increasing concentrations of NosA for 72 hours followed by cell viability measurement using CellTiter-Glo luminescence assay. Data are from two independent experiments, each including triplicate measurements.

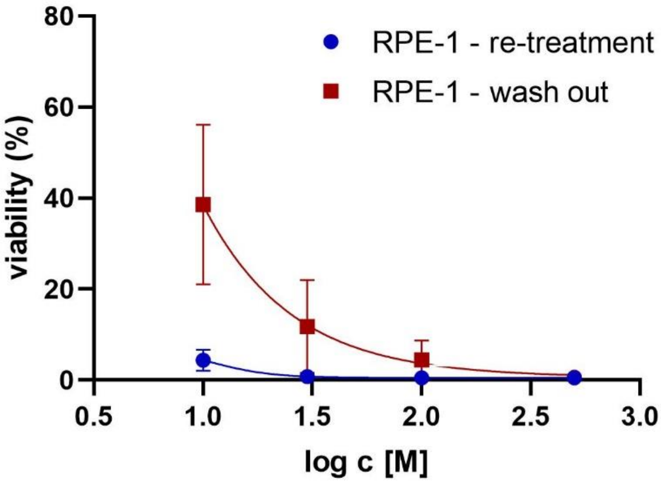

Figure S34: Viability of RPE-1 cells after long-term (168 h) NosA treatment in different concentrations with compound washout or retreatment performed after 72 h.

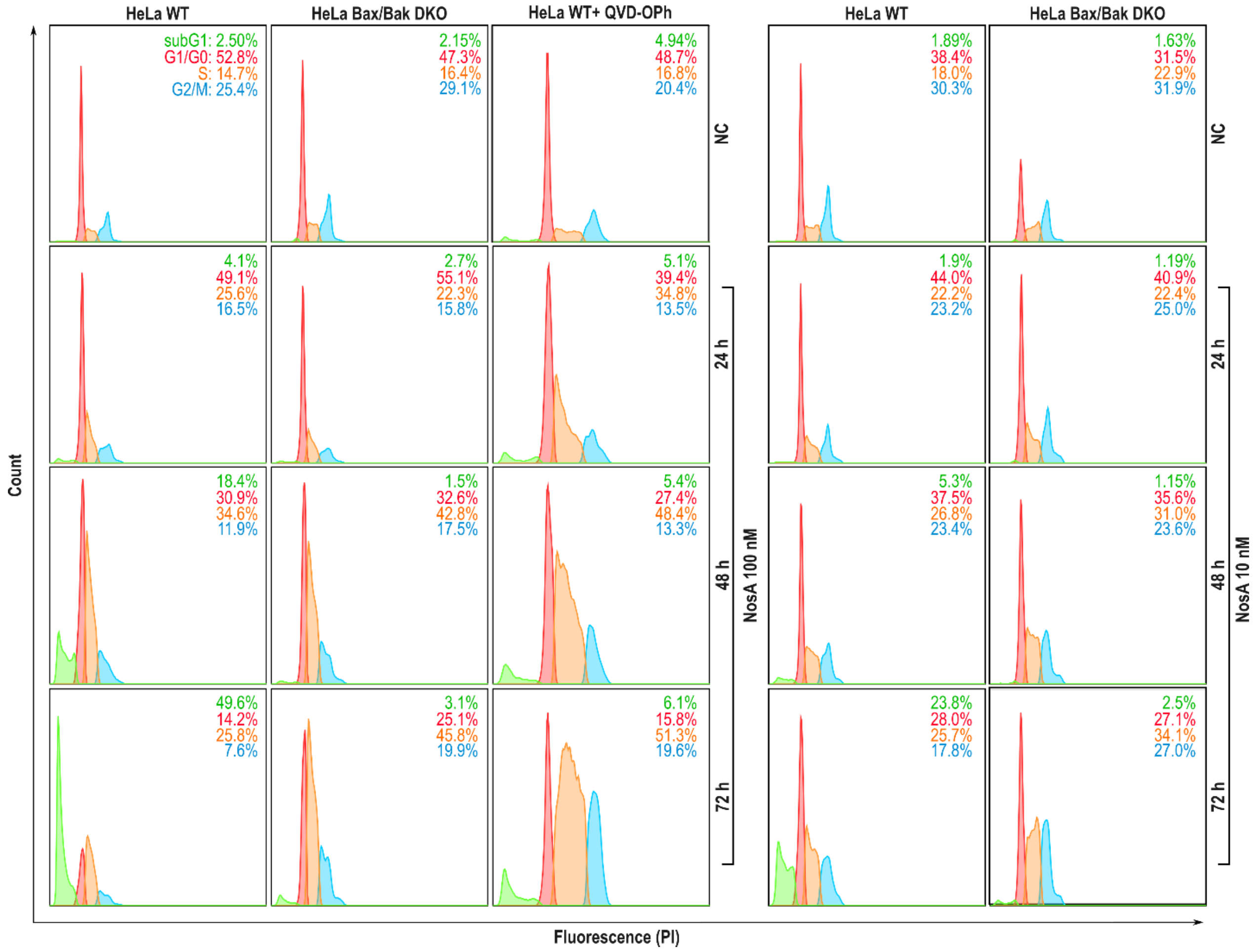

Figure S35: Cell cycle analysis of asynchronous HeLa cells treated with 100 nM (A) and 10 nM (B) NosA in HeLa WT, HeLa Bax/Bak DKO, and Hela WT pretreated with QVD-OPh. NC stands for negative control.

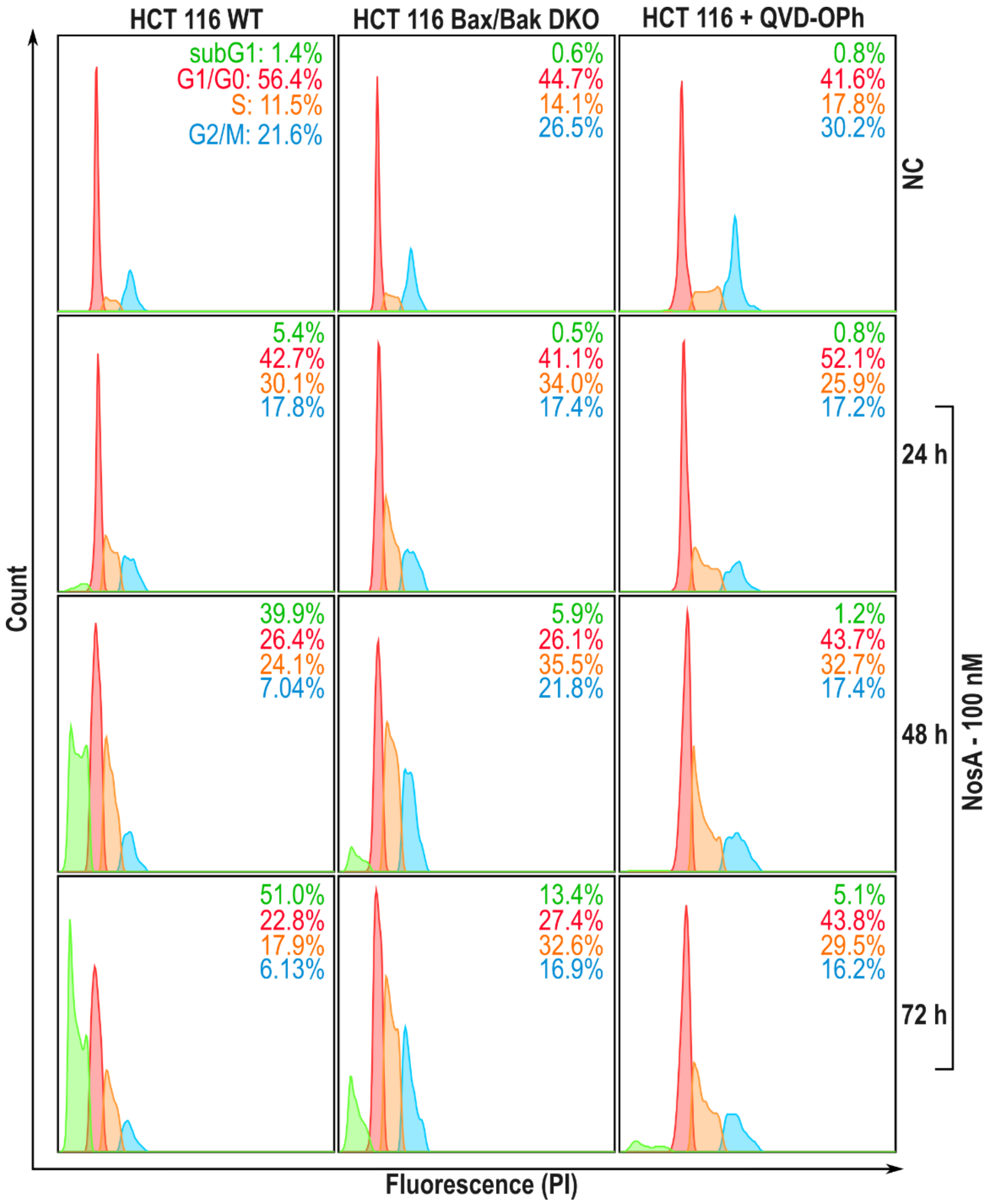

Figure S36: Cell cycle analysis of asynchronous HCT-116 cells treated with 100 nM NosA. NC stands for negative control.

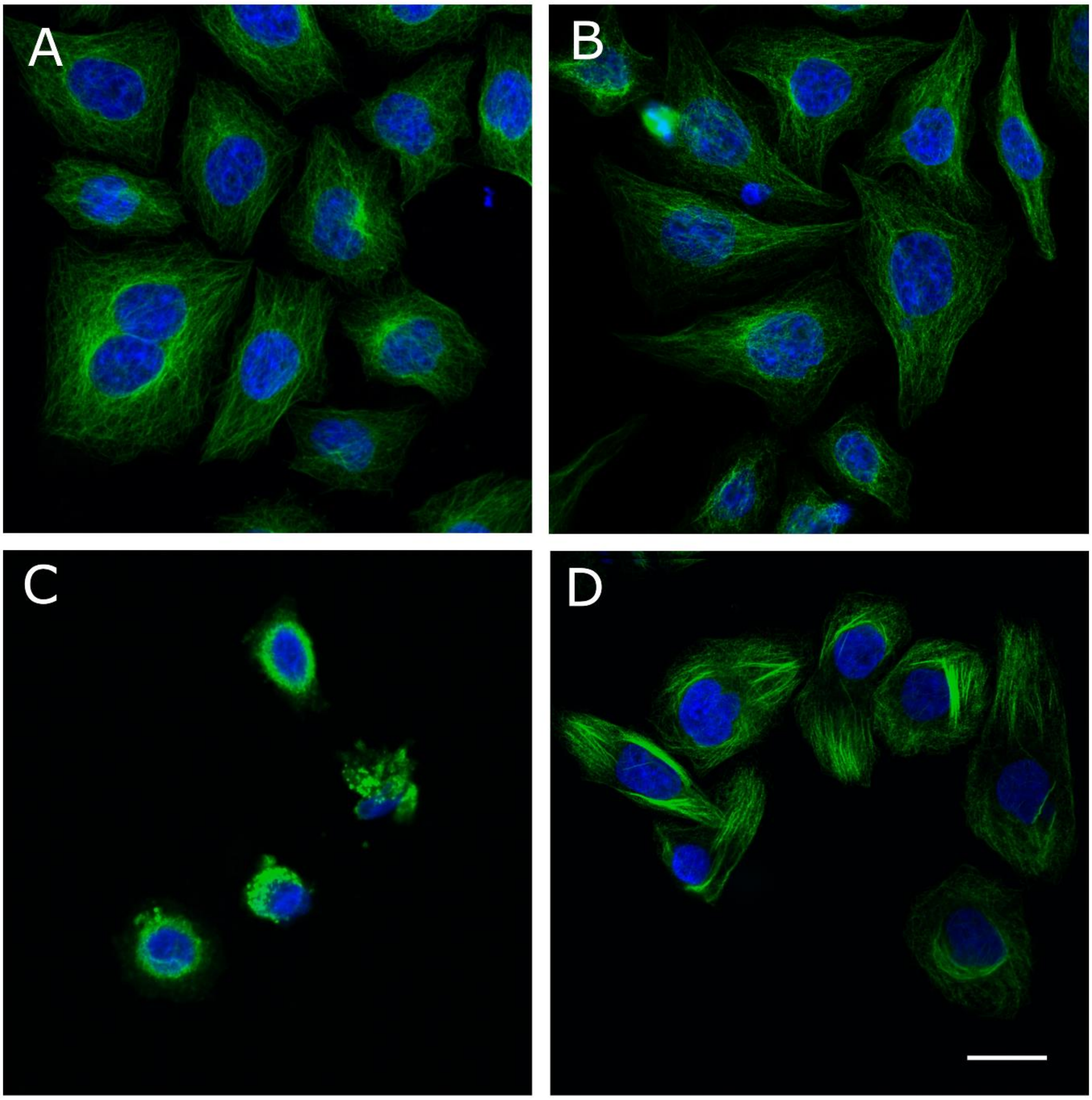

Figure S37: Immunofluorescence confocal microscopy images of HeLa WT cells treated with vehicle (A), NosA (B), Taxol (C), and MMAF (D). The nuclei and microtubules have been stained with DAPI (blue) and α-tubulin antibody (green), respectively.

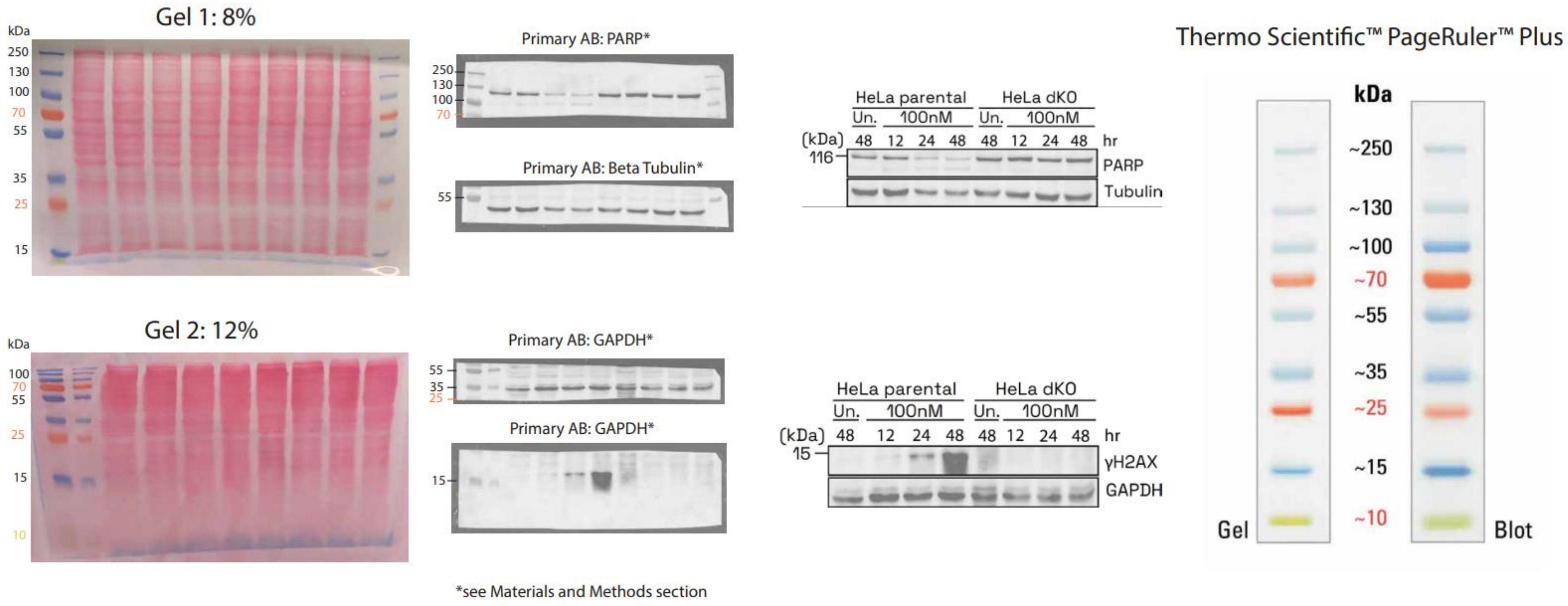

Figure S38: Immunofluorescence confocal microscopy images of HeLa WT cells treated with vehicle (A), NosA (B), Taxol (C), and MMAF (D). The nuclei and microtubules have been stained with DAPI (blue) and α-tubulin antibody (green), respectively.

Supplementary references

1. M. B. Allen and D. I. Arnon, Studies on notrogen-fixing blue-green alagae. 1. growth and nitrogen fixation by Anabaena Cylindrica Lemm., *Plant Physiology*, 1955, **30**, 366-372.
2. S. Opekar, H. Zahradnícková, P. Vodrázka, L. Rimnácová, P. Simek and M. Moos, A chiral GC-MS method for analysis of secondary amino acids after heptafluorobutyl chloroformate & methylamine derivatization, *Amino Acids*, 2021, **53**, 347-358.
3. H. Zahradnícková, S. Opekar, L. Rimnácová, P. Simek and M. Moos, Chiral secondary amino acids, their importance, and methods of analysis, *Amino Acids*, 2022, **54**, 687-719.
4. J. Mares, J. Hájek, P. Urajová, J. Kopecky and P. Hrouzek, A Hybrid Non-Ribosomal Peptide/Polyketide Synthetase Containing Fatty-Acyl Ligase (FAAL) Synthesizes the β-Amino Fatty Acid Lipopeptides Puwainaphycins in the *Cylindrospermum alatosporum*, *Plos One*, 2014, **9**.
5. A. Bankevich, S. Nurk, D. Antipov, A. A. Gurevich, M. Dvorkin, A. S. Kulikov, V. M. Lesin, S. I. Nikolenko, S. Pham, A. D. Prjibelski, A. V. Pyshkin, A. V. Sirotkin, N. Vyahhi, G. Tesler, M. A. Alekseyev and P. A. Pevzner, SPAdes: A New Genome Assembly Algorithm and Its Applications to Single-Cell Sequencing, *Journal of Computational Biology*, 2012, **19**, 455-477.
6. D. H. Parks, M. Imelfort, C. T. Skennerton, P. Hugenholtz and G. W. Tyson, CheckM: assessing the quality of microbial genomes recovered from isolates, single cells, and metagenomes, *Genome Research*, 2015, **25**, 1043-1055.
7. K. Blin, S. Shaw, H. E. Augustijn, Z. L. Reitz, F. Biermann, M. Alanjary, A. Fetter, B. R. Terlouw, W. W. Metcalf, E. J. N. Helfrich, G. P. van Wezel, M. H. Medema and T. Weber, antiSMASH 7.0: new and improved predictions for detection, regulation, chemical structures and visualisation, *Nucleic Acids Research*, 2023, **51**, W46-W50.
8. A. Marchler-Bauer, M. K. Derbyshire, N. R. Gonzales, S. N. Lu, F. Chitsaz, L. Y. Geer, R. C. Geer, J. He, M. Gwadz, D. I. Hurwitz, C. J. Lanczycki, F. Lu, G. H. Marchler, J. S. Song, N. Thanki, Z. X. Wang, R. A. Yamashita, D. C. Zhang, C. J. Zheng and S. H. Bryant, CDD: NCBI's conserved domain database, *Nucleic Acids Research*, 2015, **43**, D222-D226.
9. B. R. Terlouw, K. Blin, J. C. Navarro-Muñoz, N. E. Avalon, M. G. Chevrette, S. Egbert, S. Lee, D. Meijer, M. J. J. Recchia, Z. L. Reitz, J. A. van Santen, N. Selem-Mojica, T. Torring, L. Zaroubi, M. Alanjary, G. Aleti, C. Aguilar, S. A. A. Al-Salihi, H. E. Augustijn, J. A. Avelar-Rivas, L. A. Avitia-Domínguez, F. Barona-Gómez, J. Bernaldo-Agüero, V. A. Bielinski, F. Biermann, T. J. Booth, V. J. C. Bravo, R. Castelo-Branco, F. O. Chagas, P. Cruz-Morales, C. Du, K. R. Duncan, A. Gavriilidou, D. Gayrard, K. Gutiérrez-García, K. Haslinger, E. J. N. Helfrich, J. J. J. van der Hooft, A. P. Jati, E. Kalkreuter, N. Kalyvas, K. B. Kang, S. Kautsar, W. Kim, A. M. Kunjapur, Y. X. Li, G. M. Lin, C. Loureiro, J. J. R. Louwen, N. L. L. Louwen, G. Lund, J. Parra, B. Philmus, B. Pourmohsenin, L. J. U. Pronk, A. Rego, D. A. B. Rex, S. Robinson, L. R. Rosas-Becerra, E. T. Roxborough, M. A. Schorn, D. J. Scobie, K. S. Singh, N. Sokolova, X. Y. Tang, D. Udwary, A. Vigneshwari, K. Vind, S. Vromans, V. Waschulin, S. E. Williams, J. M. Winter, T. E. Witte, H. L. Xie, D. Yang, J. W. Yu, M. Zdouc, Z. Zhong, J. Collemare, R. G. Linington, T. Weber and M. H. Medema, MIBiG 3.0: a community-driven effort to annotate experimentally validated biosynthetic gene clusters, *Nucleic Acids Research*, 2023, **51**, D603-D610.
10. R. C. Edgar, MUSCLE: multiple sequence alignment with high accuracy and high throughput, *Nucleic Acids Research*, 2004, **32**, 1792-1797.
11. H. Onaka, M. Nakaho, K. Hayashi, Y. Igarashi and T. Furumai, Cloning and characterization of the goadsporin biosynthetic gene cluster from *Streptomyces* sp. TP-A0584, *Microbiology-Sgm*, 2005, **151**, 3923-3933.
12. M. Suzuki, H. Komaki, I. Kaweewan, H. Dohra, H. Hemmi, H. Nakagawa, H. Yamamura, M. Hayakawa and S. Kodani, Isolation and structure determination of new linear azole-containing peptides spongiicolazolicins A and B from *Streptomyces* sp. CWH03, *Applied Microbiology and Biotechnology*, 2021, **105**, 93-104.

13. D. Y. Travin, Z. L. Watson, M. Metelev, F. R. Ward, I. A. Osterman, I. M. Khven, N. F. Khabibullina, M. Serebryakova, P. Mergaert, Y. S. Polikanov, J. H. D. Cate and K. Severinov, Structure of ribosome-bound azole-modified peptide phazolicin rationalizes its species-specific mode of bacterial translation inhibition, *Nature Communications*, 2019, **10**.
14. N. Mahanta, G. A. Hudson and D. A. Mitchell, Radical *S*-Adenosylmethionine Enzymes Involved in RiPP Biosynthesis, *Biochemistry*, 2017, **56**, 5229-5244.
15. M. F. Freeman, M. J. Helf, A. Bhushan, B. I. Morinaka and J. Piel, Seven enzymes create extraordinary molecular complexity in an uncultivated bacterium, *Nature Chemistry*, 2017, **9**, 387-395.
16. Z. Q. Wang, N. Forelli, Y. Hernandez, M. Ternei and S. F. Brady, Lapcin, a potent dual topoisomerase I/II inhibitor discovered by soil metagenome guided total chemical synthesis, *Nature Communications*, 2022, **13**.
17. B. L. Spaller, J. M. Trieu and P. F. Almeida, Hemolytic Activity of Membrane-Active Peptides Correlates with the Thermodynamics of Binding to 1-Palmitoyl-2-Oleoyl-*sn*-Glycero-3-Phosphocholine Bilayers, *Journal of Membrane Biology*, 2013, **246**, 257-262.
18. A. Espinel-Ingroff, B. Arthington-Skaggs, N. Iqbal, D. Ellis, M. A. Pfaller, S. Messer, M. Rinaldi, A. Fothergill, D. L. Gibbs and A. Wang, Multicenter evaluation of a new disk agar diffusion method for susceptibility testing of filamentous fungi with voriconazole, posaconazole, itraconazole, amphotericin B, and caspofungin, *Journal of Clinical Microbiology*, 2007, **45**, 1811-1820.
19. G. Martano, N. Delmotte, P. Kiefer, P. Christen, D. Kentner, D. Bumann and J. A. Vorholt, Fast sampling method for mammalian cell metabolic analyses using liquid chromatography-mass spectrometry, *Nature Protocols*, 2015, **10**, 1-11.
20. M. Moos, J. Korbelova, T. Stetina, S. Opekar, P. Simek, R. Grgac and V. Kostal, Cryoprotective Metabolites Are Sourced from Both External Diet and Internal Macromolecular Reserves during Metabolic Reprogramming for Freeze Tolerance in Drosophilid Fly, *Chymomyza costata*, *Metabolites*, 2022, **12**.
21. Y. L. Chen, J. X. Wang, G. Q. Li, Y. P. Yang and W. Ding, Current Advancements in Sactipeptide Natural Products, *Frontiers in Chemistry*, 2021, **9**.
22. P. Simek, P. Husek and H. Zahradnícková, Gas chromatographic-mass spectrometric analysis of biomarkers related to folate and cobalamin status in human serum after dimercaptopropanesulfonate reduction and heptafluorobutyl chloroformate derivatization, *Analytical Chemistry*, 2008, **80**, 5776-5782.
23. H. Frank, W. Woiwode, G. Nicholson and E. Bayer, Determination of the rate of acidic catalyzed racemization of protein amino-acids, *Liebigs Annalen Der Chemie*, 1981, 354-365.
24. S. Kadlcík, T. Kucera, D. Chalupská, R. Gazák, M. Koberská, D. Ulanová, J. Kopecky, E. Kutejová, L. Najmanová and J. Janata, Adaptation of an L-Proline Adenylation Domain to Use 4-Propyl-L-Proline in the Evolution of Lincosamide Biosynthesis, *Plos One*, 2013, **8**.
